# Discovery of Marine-Inspired Guanidine-Based PDE4 Inhibitors for the Treatment of Chronic Obstructive Pulmonary Disease

**DOI:** 10.3390/md24030090

**Published:** 2026-02-25

**Authors:** Xinglong Dai, Jie Hao, Yan Zhang, Yaping Yang, Wanli Meng, Fang Lu, Jianchun Zhao, Guanhua Du, Shengbiao Wan, Jiejie Hao

**Affiliations:** 1 Laboratory for Marine Drugs and Bioproducts, Qingdao Marine Science and Technology Center, School of Medicine and Pharmacy, Ocean University of China, Qingdao 266003, China; dxl7912@stu.ouc.edu.cn (X.D.); hj13561151809@163.com (J.H.); zyola221199@163.com (Y.Z.); yaping0111@163.com (Y.Y.); mengwanli0323@163.com (W.M.); lfangouc@163.com (F.L.); biaowan@ouc.edu.cn (S.W.); 2 Marine Biomedical Research Institute of Qingdao, Qingdao 266003, China; zhaojianchun@ouc.edu.cn (J.Z.); dugh@imm.ac.cn (G.D.)

**Keywords:** anti-inflammatory, COPD, PDE4 inhibitor, cAMP-PKA-CREB

## Abstract

Chronic obstructive pulmonary disease (COPD) is a chronic inflammatory respiratory disorder with a globally increasing prevalence. Current therapeutic strategies are limited by drug resistance and safety concerns. Studies suggest that inhibiting the secretion of inflammatory cytokines represents a promising approach for COPD treatment. Phosphodiesterase-4 (PDE4) inhibitors have emerged as potent anti-inflammatory agents for respiratory diseases. In this study, we integrated a marine-derived natural product with computer-aided drug design to develop 32 novel PDE4 inhibitors. Compound **B7** exhibited potent PDE4 inhibitory activity and a favorable safety profile. In rat model of COPD, **B7** significantly reduced inflammatory cell infiltration and cytokine levels, ameliorated pathological changes in the lung, decreased the percentage of goblet cell positivity, and reduced expiratory resistance. Furthermore, in vitro mechanistic studies revealed that **B7** exerts its anti-inflammatory effects by activating the cAMP-PKA-CREB signaling pathway and suppressing the NF-κB pathway in RAW264.7 cells. In conclusion, **B7** demonstrates potential as a safe and effective PDE4-targeted candidate for the treatment of COPD.

## 1. Introduction

Chronic obstructive pulmonary disease (COPD) is a chronic respiratory condition characterized by persistent airflow limitation. Its typical clinical manifestations include progressively worsening dyspnea, chronic cough, and sputum production [1,2,3]. Currently, there are approximately 400 million COPD patients worldwide [4]. COPD remains a major global health challenge with persistently high morbidity and mortality rates, currently ranking as the third leading cause of death worldwide. Studies indicate that the pathogenesis of COPD is closely associated with chronic exposure to harmful environmental factors such as cigarette smoke (CS) and air pollution, which induce airway inflammatory responses [5,6]. Data demonstrate that the risk of developing COPD in smokers and secondhand smoke-exposed individuals is 1.87 and 1.57 times higher, respectively, than that in non-smokers [7,8]. CS damages airway epithelial cells, leading to airway inflammation and triggering the release of reactive oxygen species (ROS) by various inflammatory cells (e.g., macrophages and neutrophils). Excessive ROS activates transcription factors such as NF-κB, promoting the expression and substantial release of inflammatory cytokines (e.g., IL-6 and TNF-α), thereby exacerbating inflammatory responses and causing severe damage to alveolar cells [9,10,11]. Additionally, impaired cells facilitate the infiltration of inflammatory cells into the lungs, resulting in the excessive production of inflammatory triggers and cytokine storms that cause severe pulmonary damage [12,13]. Studies have shown that macrophages in the airways, bronchoalveolar lavage fluid (BALF), lung parenchyma, and the sputum of COPD patients are significantly increased compared with those in healthy samples, and macrophage numbers correlate with the severity of emphysema [14]. These findings suggest that inhibiting the secretion of inflammatory cytokines represents a promising therapeutic strategy for COPD and related pulmonary diseases.

**Figure 1:**
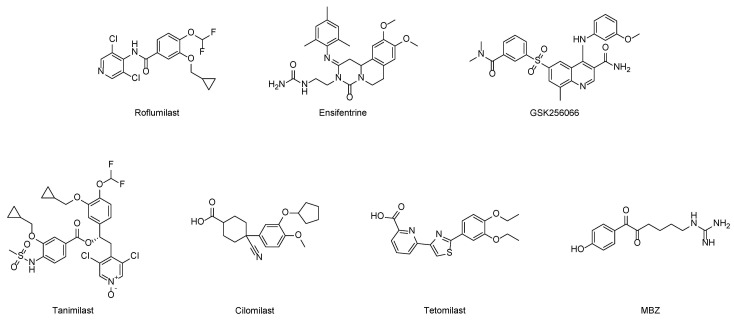
Structures of representative PDE4 inhibitors for pulmonary inflammation. MBZ, a lead compound discovered by our group, displays specific inhibitory activity toward the PDE4B isoform and marked anti-acute lung injury effects.

Current pharmacological therapies for COPD include corticosteroids, β_2_-adrenergic receptor agonists, anticholinergics, and theophylline [15,16]. While these agents alleviate COPD symptoms, they are also associated with adverse effects such as growth suppression, tachycardia, and gastrointestinal disturbances [17,18]. In recent years, PDE4 inhibitors have garnered significant research interest for the treatment of pulmonary and respiratory diseases [19]. PDE4 is a key enzyme responsible for hydrolyzing cyclic adenosine monophosphate (cAMP), which serves as a second messenger regulating numerous physiological processes and inflammatory diseases [20]. Increased cAMP levels attenuate the activity of critical inflammatory cells, such as eosinophils and neutrophils, thereby inhibiting cytokine release (e.g., TNF-α, IL-13), reactive oxygen species generation, and cell migration [21,22,23,24]. Consequently, PDE4 has emerged as a promising therapeutic target for inflammatory diseases [17,25,26]. Roflumilast, approved by the European Union in 2010 for COPD [27], has also been validated as an add-on therapy to inhaled corticosteroids for asthma [28]. Cilomilast is in phase III clinical trials for the treatment of COPD [29]. The PDE3/4 dual inhibitor Ensifentrine has received FDA approval for COPD treatment [30]. GSK256066 has been developed as an inhaled therapeutic for asthma, while Tanimilast is undergoing Phase II clinical trials for asthma [31]. Tetomilast (OPC-6535), currently in Phase III trials for Crohn’s disease and ulcerative colitis, is also being evaluated in Phase II trials for COPD [29,32] (Figure 1). However, the clinical utility of drugs such as Roflumilast is severely hindered by significant gastrointestinal adverse effects—particularly nausea and diarrhea—resulting from oral administration. These side effects are attributed to the non-selective inhibition across the (PDE4) family. The PDE4 family comprises four distinct isoforms (PDE4A–D), each possessing unique tissue distributions and physiological roles. Specifically, PDE4B is predominantly expressed in immune cells and serves as a pivotal target for inflammatory diseases by mediating the release of pro-inflammatory cytokines. In contrast, PDE4D is extensively involved in central nervous system processes, including cognition and neuroregeneration; its inhibition is widely recognized as the primary driver of dose-limiting toxicities such as nausea and emesis [33,34]. This clinical bottleneck highlights the pressing need for next-generation PDE4 inhibitors that exhibit a high isoform selectivity and an enhanced safety profile to widen the therapeutic window.

In this study, the natural alkaloid MBZ (Figure 1)—isolated from marine bryozoans and hydroids and exhibiting PDE4B inhibitory activity—was used as a lead compound. Through structure-guided optimization via molecular docking, five novel series comprising 32 PDE4 inhibitor derivatives were designed and synthesized. Compound **B7** demonstrates PDE4 inhibitory effects and potent anti-inflammatory efficacy in both cellular assays and in vivo models.

## 2. Results and Discussion

### 2.1. Structure-Based Molecular Design

**Figure 2:**
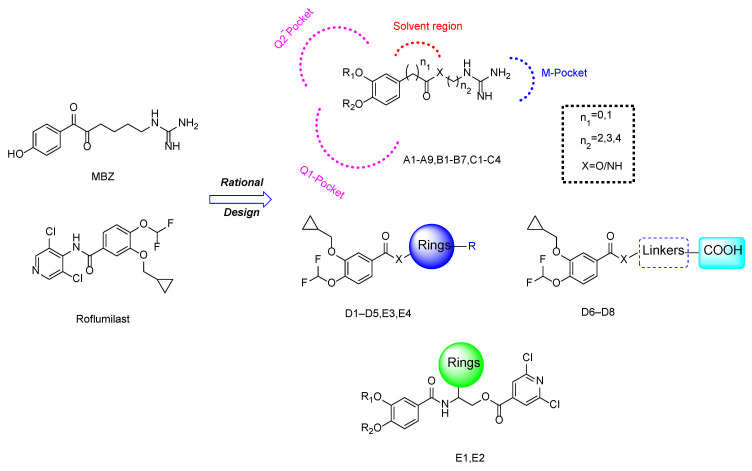
Design atrategy of PDE4 inhibitors based on natural products and Roflumilast.

Our group identified MBZ, a natural product exhibiting PDE4 inhibitory activity (IC_50_ = 91.93 ± 0.27 μM against PDE4B), through screening a marine natural product database. Based on the structural characteristics of MBZ and Roflumilast, in conjunction with structural analyses of catechol ether-based PDE4 inhibitors, it is observed that the Q2 pocket accommodates bulky hydrophobic cycloalkyl substituents, while the smaller Q1 pocket selectively binds methoxy or difluoromethoxy moieties. These groups facilitate key hydrogen-bonding interactions with the conserved Gln443 residue. Furthermore, the catechol scaffold is stabilized by pi-pi stacking interactions with the adjacent Phe446 residue within the catalytic domain. These interactions may represent critical determinants of the inhibitory activity [24,35]. Given the established association between guanidinium groups and diverse bioactivities [36,37], we designed and synthesized 20 derivatives (**A1**–**A9**, **B1**–**B7**, **C1**–**C4**) featuring diversified substitutions at positions 3 and 4 of the phenyl ring, along with variable-length linkers to the guanidinium moiety (Figure 2).

Inspired by the structural features of our previously identified lead compound MBZ and the clinical PDE4 inhibitor Roflumilast, a pharmacophore hybridization and scaffold morphing strategy were employed to develop two novel series of derivatives, designated as Series D and Series E. The core catechol scaffold of Roflumilast, characterized by its signature difluoromethoxy and cyclopropylmethoxy groups, was maintained as the primary binding motif to ensure essential interactions within the Q1 and Q2 pockets of the PDE4 catalytic site [35,36,37]. In an effort to further explore the chemical space of the M-pocket and the solvent-exposed region, the basic guanidine moiety of MBZ was strategically replaced. Specifically, various substituted ring systems were introduced as bioisosteres to yield compounds **D1**–**D5**, **E3** and **E4**. Furthermore, a terminal carboxylic acid group was incorporated via diverse linkers in compounds **D6**–**D8** to investigate potential ionic interactions and optimize the overall physicochemical profile. Notably, **E1** and **E2** were designed to simultaneously retain the critical pharmacophores of Roflumilast—the dichloropyridine ring and the catechol moiety—while utilizing bulky hydrophobic aromatic rings on the side chain to further extend into the hydrophobic S-subpocket, thereby maximizing binding affinity and structural diversity.

### 2.2. Chemistry

**Scheme 1:**
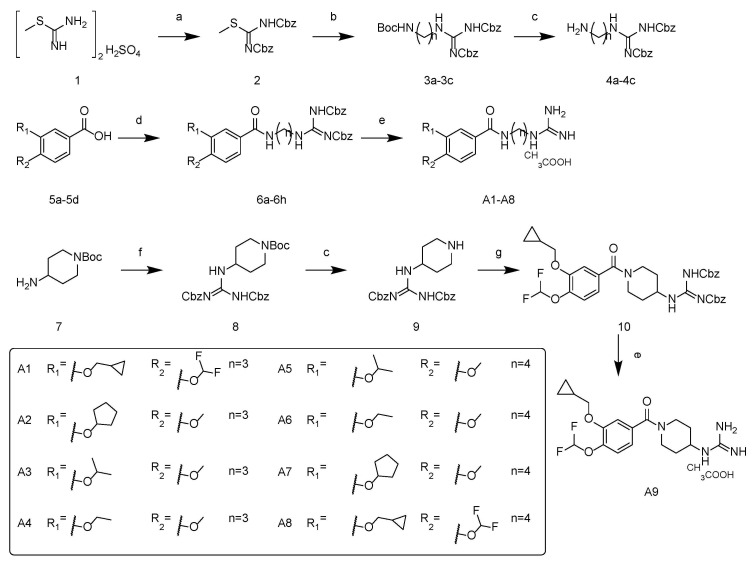
Synthesis of **A1**–**A9**. Reagents and conditions: (a) benzyl chloroformate, NaOH, NaHCO_3_, H_2_O, r.t., 8 h; (b) mono-Boc-protected alkyldiamines, DCM, Et_3_N, r.t., 5 h; (c) DCM, TFA, 0 °C, 2 h; (d) **4a**–**4c**, DCM, EDCI, HOBt, r.t., 8 h; (e) MeOH, H_2_, Pd/C, AcOH, r.t., 2 h; (f) 2, DCM, Et_3_N, r.t., 5 h; (g) 3-(cyclopropylmethoxy)-4-(difluoromethoxy)benzoic acid, DCM, EDCI, HOBt, r.t., 8 h.

The synthetic route for the target compounds **A1**–**A9** is outlined in Scheme 1. S-Methylisothiourea sulfate **1** was treated with benzyl chloroformate to afford the thiocarbamate intermediate **2**. Intermediate **2** was then reacted with mono-Boc-protected alkyldiamines of varying chain lengths to provide the key precursors **3a**–**3c**. The acid-mediated deprotection (Boc removal) of **3a**–**3c** yielded the amine intermediates **4a**–**4c**. These intermediates were subjected to amidation with substituted benzoic acid derivatives **5a**–**5d** to furnish intermediates **6a**–**6h**. Finally, the hydrogenolytic removal of the Cbz group catalyzed by Pd/C delivered the target compounds **A1**–**A8**. Compound **A9** was prepared starting from N-Boc-4-piperidinamine following the same synthetic sequence described above.

**Scheme 2:**
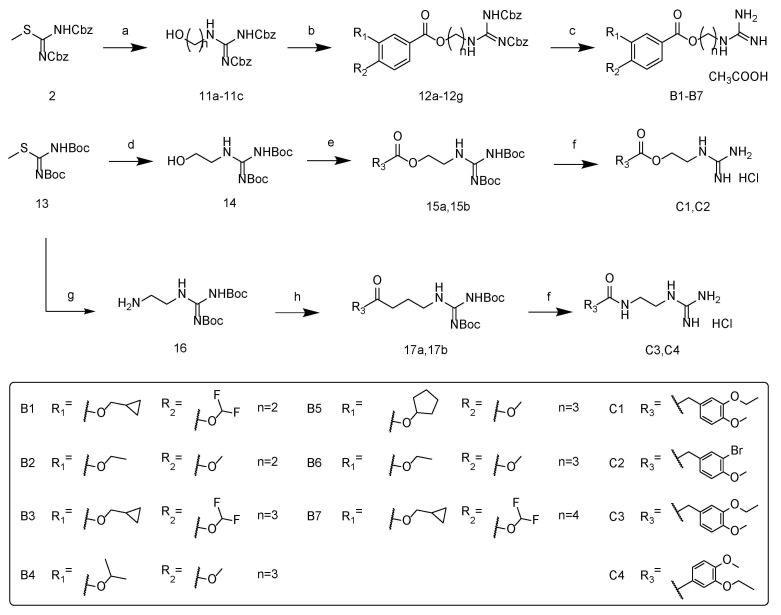
Synthesis of **B1**–**B7** and **C1**–**C4**. Reagents and conditions: (a) amino alcohols, DCM, Et_3_N, r.t., 5 h; (b) 5a–5d, DCM, EDCI, DMAP, r.t., 8 h; (c) MeOH, H_2_, Pd/C, AcOH, r.t., 2 h; (d) monoethanolamine, DCM, Et_3_N, r.t., 5 h; (e) aromatic carboxylic acids, MeOH, DCM, EDCI, DMAP, r.t., 8 h; (f) i. DCM, TFA, 0 °C, 2 h; ii. 0.5 N HCl r.t., 1 h; (g) ethylenediamine, DCM, Et_3_N, r.t., 5 h; (h) aromatic carboxylic acids, MeOH, DCM, EDCI, DMAP, r.t., 8 h.

Compounds **B1** to **B7** were synthesized starting from intermediate **2**, which was sequentially subjected to substitution with amino alcohols of varying chain lengths, followed by esterification and deprotection reactions. Compounds **C1**–**C4** were synthesized by reacting N, N′-di-Boc-S-methylisothiourea with monoethanolamine or ethylenediamine to afford intermediates **14** and **16**, respectively, followed by coupling with disubstituted benzoic or phenylacetic acids to generate precursors **15a**, **15b**, **17a**, and **17b**. Subsequent Boc deprotection yielded the target compounds. Notably, these guanidinium hydrochloride salts exhibited lower solidification tendencies, higher viscosity, and reduced Boc deprotection yields compared to acetate salts, prompting the selection of Cbz protection for guanidine groups (Scheme 2).

**Scheme 3:**
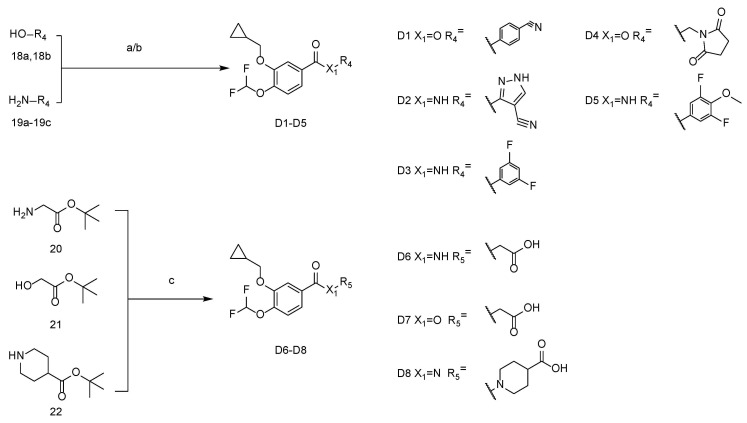
Synthesis of **D1**–**D8**. Reagents and conditions: (a) 3-(cyclopropylmethoxy)-4-(difluoromethoxy)benzoic acid, DCM, EDCI, HOBt, r.t., 8 h; (b) 3-(cyclopropylmethoxy)-4-(difluoromethoxy)benzoic acid, DCM, (COCl)_2_, r.t., 2 h; (c) HCOOH, r.t., 7 h.

**Scheme 4:**
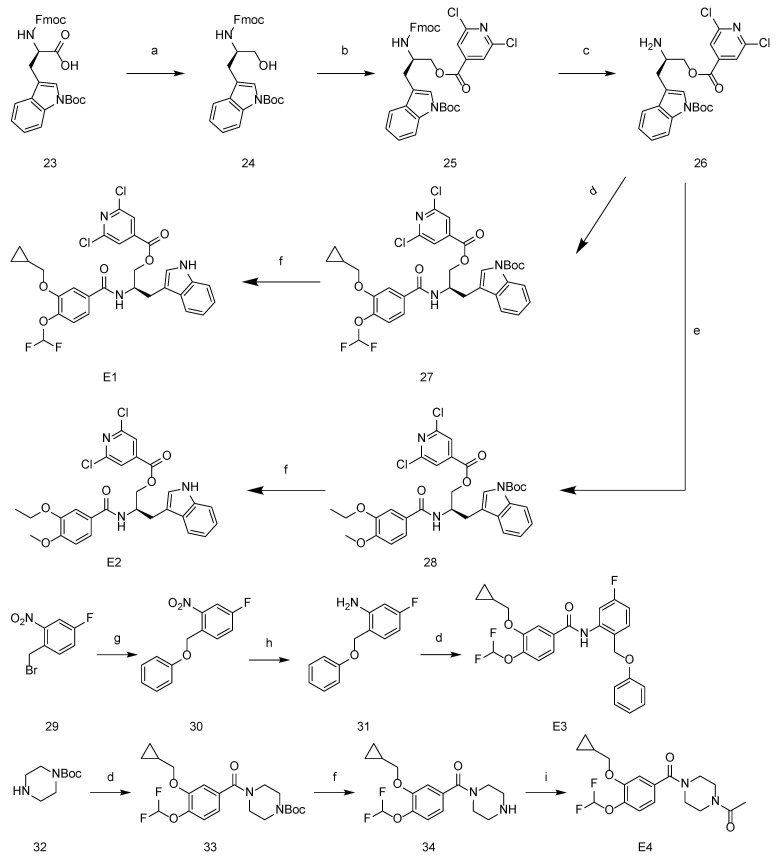
Synthesis of **E1**–**E4**. Reagents and conditions: (a) DME, NMM, IBCF, NaBH_4_, −15 °C, 1 h; (b) DCM, oxalyl chloride, DMF, 0 °C, 6 h; (c) DMF, piperidine, r.t., 0.5 h; (d) 3-(cyclopropylmethoxy)-4-(difluoromethoxy)benzoic acid, DCM, EDCI, DMAP, r.t., 10 h; (e) 3-ethoxy-4-methoxybenzoic acid, DCM, EDCI, DMAP, r.t., 10 h; (f) DCM, TFA, 0 °C, 1 h; (g) phenol, MeCN, K_2_CO_3_, KI, 85 °C, 3 h; (h) EtOH, Fe, HCl, 50 °C, 2 h; (i) acetyl chloride, DCM, Et_3_N,0 °C, 2 h.

The synthetic routes for compounds **D1**–**D8** and **E1**–**E4** are delineated in Scheme 3 and Scheme 4. Compounds **D1**–**D5** were synthesized through the condensation of 3-(cyclopropylmethoxy)-4-(difluoromethoxy)benzoic acid with substituted aromatic amines, phenols, or heterocyclic alcohols, while **D6**–**D8** were obtained through the condensation of t-Bu-protected hydroxy/amino carboxylic acids with 3-(cyclopropylmethoxy)-4-(difluoromethoxy)benzoic acid followed by deprotection. Compounds **E1** and **E2** were synthesized from Fmoc-Trp(Boc)-OH through carboxyl group reduction to hydroxymethyl, a subsequent reaction with acid chlorides affording intermediate **25**, Fmoc deprotection yielding intermediate **26**, and a final amidation with disubstituted benzoic acids. Compound **E3** was prepared from 4-fluoro-2-nitrobenzyl bromide via sequential nucleophilic substitution, reduction, and amidation. Compound **E4** was synthesized from Boc-piperazine through an initial amidation, Boc deprotection, and subsequent N-acylation.

### 2.3. Structure–Activity Relationship

We measured the inhibition of synthesized compounds **A1**–**A9**, **B1**–**B7**, **C1**–**C4**, **D1**–**D8** and **E1**–**E4** (Table 1) on recombinant full-length PDE4B activity in vitro. Among guanidine-containing compounds, **B7** exhibited optimal activity, with an inhibition rate of 93.49 ± 3.11%. The C series displayed generally weak activity, with only **C4** showing a marginal inhibition. This suggests that benzoic acid substituents critically influence PDE inhibition potency. Additionally, amide linkages demonstrated a superior activity to ester linkages with identical substituents. Linker length also played a pivotal role: increasing chain length enhanced activity in the B series, whereas amide-linked A series compounds showed an inverse trend. Overall, compounds bearing cyclopropyloxy and difluoromethoxy substituents on the phenyl ring exhibited a stronger inhibition at equivalent linker lengths.

The D series, which preserves this substitution pattern, demonstrated an enhanced potency. Notably, **D6** and **D7** (terminal-free carboxylates) showed exceptional activity, potentially due to carboxylate coordination with Zn^2+^/Mg^2+^ in the active-site M-region. However, six-membered nitrogen-containing heterocyclic linkers (e.g., **A9**, **D8**, **E4**) compromised activity. The low potency of the E series may be attributed to steric hindrance from bulky hydrophobic substituents impeding subpocket access.

### 2.4. The IC_50_ Values of Selected Compounds Against PDE4B

Based on preliminary screening results, several compounds exhibiting potent PDE4B inhibitory activity were selected for IC_50_ determination. As summarized in Table 2, the IC_50_ values of these compounds against PDE4B ranged from 0.9 to 5.09 μM. Notably, compound **B7** demonstrated the strongest inhibitory activity (IC_50_ = 0.9 μM), significantly lower than the lead compound MBZ (IC_50_ = 91.93 μM). The combined analysis of PDE4B inhibition rates and IC_50_ values established **B7** as a high-efficiency PDE4B inhibitor.

### 2.5. Molecular Docking and Molecular Dynamics of ***B7*** with PDE4B

**Figure 3:**
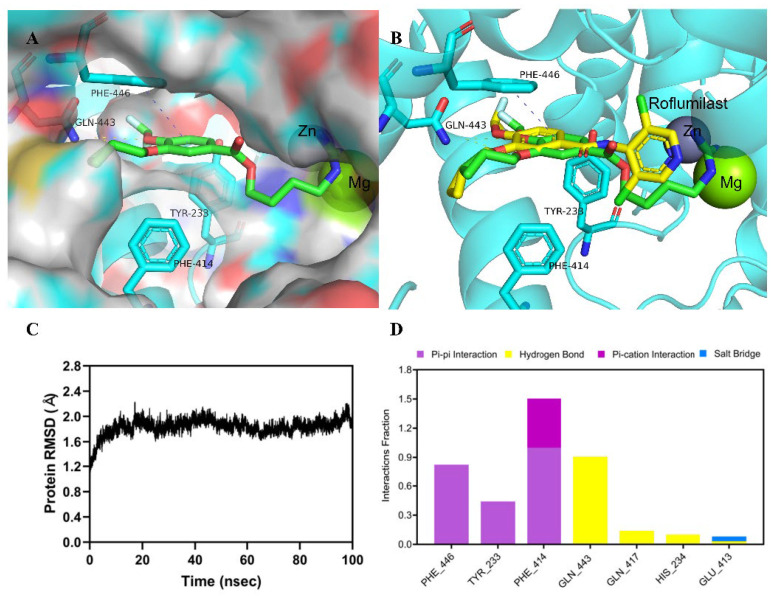
Molecular docking and dynamics simulations of **B7** to PDE4B (1XMU). (**A**) Surface model of **B7** binding to PDE4B. Yellow dotted lines represent the hydrogen bonds. Blue dotted lines represent the pi–pi interaction. (**B**) Ribbon presentation on the superposition of **B7** over Roflumilast that comes from the crystal structure of PDE4B–Roflumilast (1XMU). (**C**) The Root Mean Square Deviation (RMSD) of PDE4B’s backbone. (**D**) The main fraction of interactions between **B7** and PDE4B.

Molecular docking reveals that the difluoromethoxy and cyclopropoxy groups on **B7**’s phenyl ring occupy the Q1 and Q2 hydrophobic subpockets of the PDE4B active site, respectively, forming hydrogen bonds with Gln443. Additionally, pi–pi stacking interaction occurs between **B7**’s phenyl ring and Phe-446 in the catalytic region. This binding mode overlaps with Roflumilast interactions observed in the PDE4B–Roflumilast co-crystal structure (Figure 3A,B). Notably, **B7**’s terminal guanidinium moiety penetrates the metal-binding region, potentially forming salt bridges with metal-coordinating residues.

Molecular dynamics (MD) simulations were conducted to further investigate the binding stability of **B7** with PDE4B based on molecular docking results. Throughout the simulation, the backbone RMSD of PDE4B remained below 3.0 Å, indicating a stable protein conformation. The key residues identified in Figure 3D include Phe446, Tyr233, Phe414, Gln443, Gln417, His234, and Glu413. Remarkably, the hydrogen bond interaction fraction values between Gln443 and **B7**, along with the π-π interaction fraction values between Phe446 and **B7,** approached 1.0. These data confirm that both residues maintained specific interactions with **B7** throughout the simulation.

Additionally, the selectivity of **B7** was preliminarily evaluated through molecular docking and MD simulations, which revealed a superior binding stability within the PDE4B catalytic pocket compared to the other PDE isotypes: PDE1B, PDE3A, PDE3B, PDE4D, and PDE5A. Specifically, **B7** forms robust hydrogen bonds and pi–pi stacking interactions with key residues Gln443 and Phe446 in PDE4B—interactions that are either absent or significantly weaker in the other PDE family members (Figure S1). Correspondingly, docking scores (Table S1) confirmed that **B7** possesses the highest binding affinity for PDE4B (dG = −9.803, VS Score = −10.909).

### 2.6. The Binding of the PDE4B Protein with ***B7***

Surface plasmon resonance (SPR) analysis was employed to validate the interaction between **B7** and PDE4B (Figure S2). The value of the binding affinity (K_D_), directly reflecting the binding affinity, was determined to be 2.151 μM. This result demonstrates potent binding between **B7** and PDE4B.

### 2.7. Anti-Inflammation Activity In Vitro

In COPD, elevated levels of pro-inflammatory cytokines, including IL-6 and TNF-α, exacerbate pulmonary cellular damage and amplify inflammatory responses [11,12,38,39]. Based on these findings, compound **B7** was selected to evaluate its ability to suppress IL-6 and TNF-α secretion in lipopolysaccharide (LPS)-stimulated RAW 264.7 cells, and a preliminary investigation of its mechanism was conducted.

**Figure 4:**
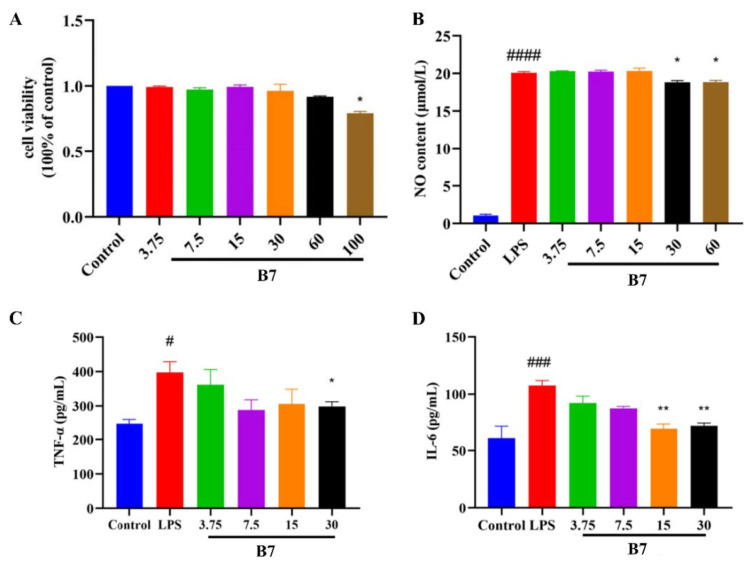
Anti-inflammation activity of **B7** in LPS-induced inflammation RAW264.7 cells. (**A**) Cell viability after treatment with various concentrations of **B7** for 24 h. Data represent the mean ± SEM (*n* = 6). * *p* < 0.05 vs. control group. (**B**) Nitrite content after LPS and **B7** treatment. Data represent the mean ± SEM (*n* = 6). #### *p* < 0.0001 vs. control group; * *p* < 0.05 vs. LPS group. (**C**) TNF-α levels after LPS and **B7** treatment. (**D**) IL-6 levels after LPS and **B7** treatment. Data represent the mean ± SEM (*n* = 6). # *p* < 0.05, ### *p* < 0.001 vs. control group; * *p* < 0.05, ** *p* < 0.01 vs. LPS group.

The cell viability of RAW 264.7 cells was assessed using the MTT assay, and nitric oxide (NO) levels in the supernatant were measured using the Griess method. As shown in Figure 4A,B, 30 and 60 μM **B7** exhibited a comparable inhibition of NO production. However, a decline in cell viability was observed at concentrations exceeding 60 μM. Therefore, subsequent experiments utilized concentrations up to 30 μM. As demonstrated in Figure 4C,D, LPS stimulation significantly increased intracellular TNF-α and IL-6 levels. **B7** treatment markedly reduced the levels of these inflammatory cytokines, indicating its potential to attenuate LPS-induced inflammatory responses. Notably, **B7** exhibited a more moderate inhibitory effect on NO production in contrast to its potent suppression of cytokine release. This disparity likely stems from the complex cAMP-mediated regulatory mechanisms of the *Nos2* gene, which remain to be further elucidated [40,41,42,43]. Such a balanced profile may offer clinical advantages by mitigating cytokine-associated inflammatory responses while preserving the basal NO levels essential for maintaining physiological homeostasis.

### 2.8. Toxicity Study of Compound ***B7*** in Rats

To evaluate the in vivo safety profile of **B7**, an acute toxicity study was conducted in SD rats divided by body weight into control (saline), low-dose (8 mg/kg), medium-dose (16 mg/kg), and high-dose (32 mg/kg) groups. All **B7** formulations were administered via tail vein injection, with the control group receiving equivalent saline volumes. During the 14-day observation period, **B7**-treated rats exhibited normal weight gain (Figure S3A).

A separate long-term toxicity assessment employed SD rats grouped as control (saline), low-dose (1.2 mg/kg), medium-dose (2.5 mg/kg), and high-dose (7.5 mg/kg) **B7**, administered identically. Over the 4-week study, all groups showed physiological weight increases (Figure S3B). Furthermore, organ-to-body weight ratios remained unchanged (Figure S4A), while H&E staining revealed no pathological alterations in heart, liver, spleen, lung, or kidney tissues (Figure S4B), collectively indicating a favorable safety profile for **B7**.

To evaluate the systemic toxicity of **B7**, serum biochemical parameters in rats were monitored. As illustrated in Figure S5, no significant alterations were observed in liver function markers (ALT, AST, ALP, and TBIL; Figure S5A–C,G), protein profiles (TP, ALB, and GLOB; Figure S5D–F), or renal function indicators (Urea and Creatinine; Figure S5H,I) compared to the control group. All parameters remained within normal physiological ranges, indicating that **B7** treatment does not induce hepatic or renal impairment at the tested dosages. These results substantiate the excellent in vivo safety and biocompatibility of **B7**, supporting its further clinical development.

### 2.9. Pharmacokinetic Profile and In Vivo Tissue Distribution of ***B7***

We evaluated the pharmacokinetic properties of **B7** administered via tail vein injection at three doses (0.2, 1, and 5 mg/kg) in Wistar rats. The terminal elimination half-life (t_1/2_) of **B7** was short (0.43–0.93 h) across the tested dose range (0.2–5 mg/kg), indicating a rapid elimination from the systemic circulation. The compound exhibits dose-proportional pharmacokinetics over the evaluated dose range, demonstrating predictable linear pharmacokinetic characteristics (Table 3).

PDE4 inhibitors induce emetic side effects, potentially due to central nervous system (CNS) distribution. The tissue distribution results indicated that **B7** primarily accumulates in the lungs, while its penetration into the brain is negligible. This phenomenon may be attributed, on the one hand, to the terminal guanidine group, which reduces blood–brain barrier permeability and thereby mitigates CNS-related adverse effects. On the other hand, the presence of aromatic hydrophobic groups and the guanidine moiety imparts a cationic amphiphilic character to the structure of **B7**, resulting in a degree of lung-targeting effect. For PDE4 inhibitors—which are primarily used to treat respiratory diseases—this profile represents a potential therapeutic advantage, as it helps enhance local drug concentrations while minimizing systemic toxicity (Figure S6).

### 2.10. ***B7*** Ameliorated Pulmonary Injury in COPD Rats

COPD is a progressive pulmonary disorder primarily classified into emphysema and chronic bronchitis, characterized by chronic inflammation and excessive proinflammatory cytokine production [44]. In this work, a COPD model was established in SD rats using LPS combined with smoke exposure. LPS challenge was performed on days 1 and 14. From days 2 to 13 and days 15 to 66, rats were placed in a sealed smoke chamber for 30 min of cigarette smoke exposure daily. On day 50, the animals were randomized into several groups. Subsequently, compound **B7** was administered via intravenous (i.v.) injection at three dose levels (low, medium, and high) for 14 consecutive days. The positive control group was treated with Roflumilast (20 mg/kg) via oral gavage (p.o.) over the same period.

**Figure 5:**
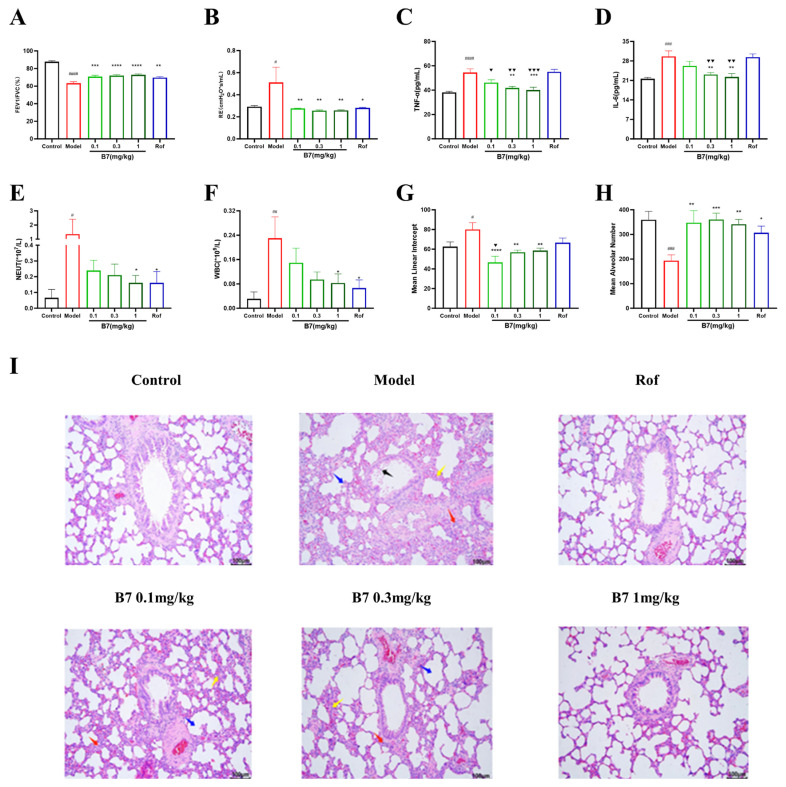
Effects of compound **B7** on COPD rats. Pulmonary function parameters in rats: FEV_1_/FVC (**A**) and RE (**B**). (**C**) The amount of TNF-α in BALF. (**D**) The amount of IL-6 in BALF. (**E**) Neutrophils in BALF. (**F**) Total inflammatory cells in BALF. (**G**) Quantification of the MAN. (**H**) Quantification of the MLI. (**I**) HE staining (200×, scale bar = 100 μm); data represent the mean ± SEM. # *p* < 0.05, ## *p* < 0.01, ### *p* < 0.001, #### *p* < 0.0001 vs. control. * *p* < 0.05, ** *p* < 0.01, *** *p* < 0.001, **** *p* < 0.0001 vs. model. ▼ *p* < 0.05, ▼▼ *p* < 0.01, ▼▼▼ *p* < 0.001, vs. Rof.

FEV_1_/FVC is a pivotal diagnostic criterion for COPD [45]. As shown in Figure 5A, a significant reduction in the FEV_1_/FVC ratio was observed in the model group relative to the control group, validating the successful induction of the COPD rat model. **B7** administration from 0.1 mg/kg to 1 mg/kg resulted in dose-dependent increases in both FEV_1_/FVC and FEV_2_/FVC ratios, showing statistically significant differences compared to the model group. Notably, **B7** at 1 mg/kg demonstrated a significantly superior efficacy to the positive control drug Roflumilast (21.42% improvement in FEV_1_/FVC), with a statistically significant difference. Additionally, **B7** significantly reduced and restored expiratory resistance in COPD rats (Figure 5B).

As shown in Figure 5C–F, the COPD model group exhibited significantly increased inflammatory cell counts and elevated levels of the inflammatory cytokines TNF-α and IL-6 in BALF. Both **B7** and Roflumilast significantly reduced total leukocyte and neutrophil counts in BALF. Furthermore, **B7** decreased TNF-α and IL-6 levels in a dose-dependent manner, demonstrating a superior efficacy to the positive control drug Roflumilast.

COPD destroys alveolar architecture, leading to the extensive peribronchial infiltration of inflammatory cells such as monocytes and neutrophils [13]. H&E staining (Figure 5I) revealed bronchial lumen narrowing, wall thickening (blue arrows), substantial inflammatory cell infiltration (red arrows), airway epithelial cell detachment (black arrows), and increased interalveolar septal thickness (yellow arrows) in the model group. The 1 mg/kg **B7** group exhibited an intact bronchial morphology with well-aligned ciliated columnar epithelial cells and no significant pathological changes. **B7** administration produced a dose-dependent amelioration of abnormal pathological alterations in COPD rats.

Furthermore, we evaluated the effects of **B7** on the mean linear intercept (MLI) and mean alveolar number (MAN) in rats. MLI reflects alveolar density, with smaller intervals indicating denser alveoli and more efficient gas exchange. The measurement procedures were performed as described previously [46,47]. Conversely, enlarged intervals suggest alveolar enlargement and impaired gas exchange. The COPD group showed a significantly increased MLI and decreased MAN. Both **B7** and Roflumilast significantly reduced MLI and increased MAN. **B7** improved gas exchange in COPD rats by increasing alveolar numbers (Figure 5G,H).

### 2.11. ***B7*** Ameliorates Airway Mucus Secretion

**Figure 6:**
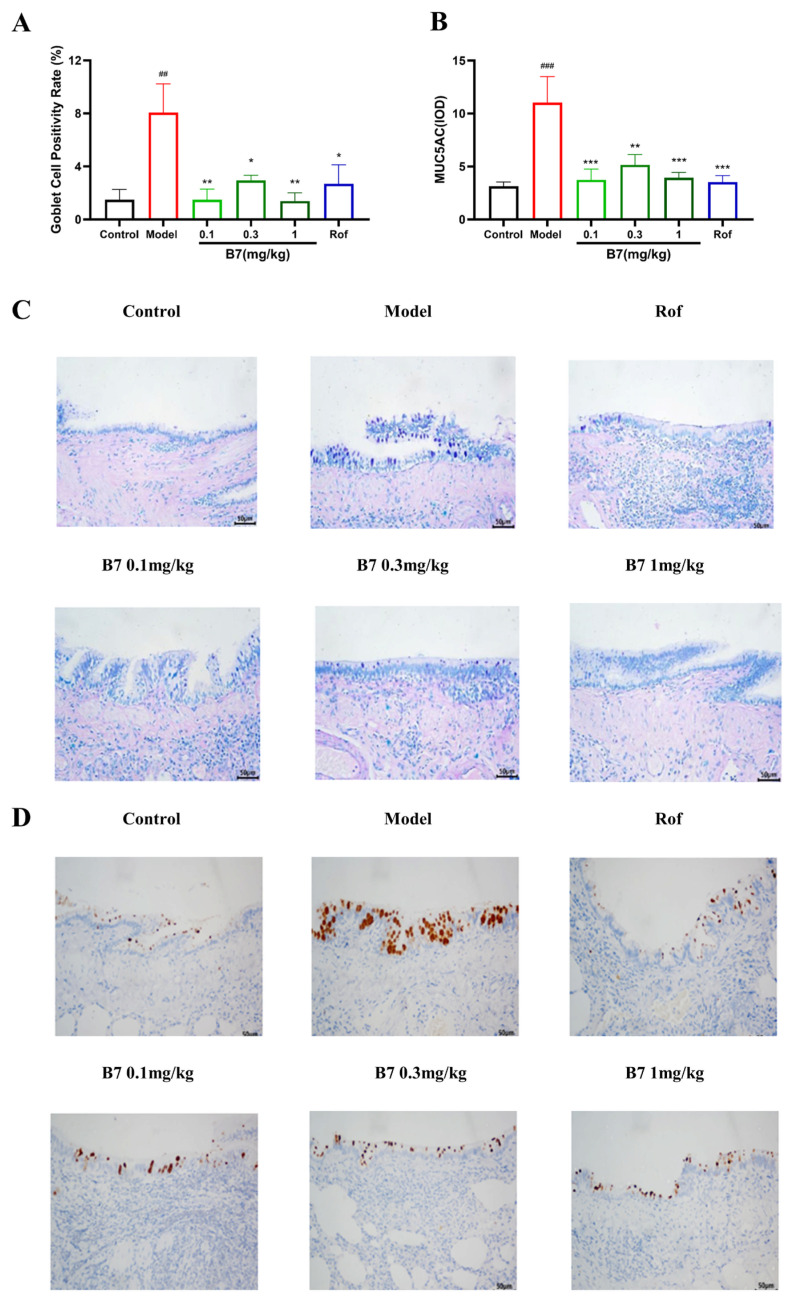
Effects of compound **B7** on goblet cell positivity and MUC5AC mucin expression in COPD rats. (**A**) Goblet cell hyperplasia in COPD rat airways. Goblet cell positivity rate was calculated as the percentage of AB-PAS-positive goblet cells relative to the total number of columnar epithelial cells (%). (**B**) MUC5AC mucin expression levels in airways of COPD rats. Semi-quantitative analysis of goblet cell hyperplasia through AB-PAS staining using integrated optical density (IOD). Data represent the mean ± SEM; ## *p* < 0.01, ### *p* < 0.001, vs. control. * *p* < 0.05, ** *p* < 0.01, *** *p* < 0.001 vs. model. (**C**) AB-PAS staining for goblet cell hyperplasia in COPD rat airways. (**D**) Inhibitory effects of **B7** on MUC5AC mucin expression in COPD rats assessed using immunohistochemical staining.

Research indicates that goblet cell hyperplasia and mucus hypersecretion play significant roles in the progression of COPD [48,49], with MUC5AC overexpression closely associated with airway mucus hypersecretion [50]. The impact of **B7** on airway mucus secretion was assessed via AB-PAS staining. The goblet cell positivity rate was significantly higher in the COPD group compared to the normal control group. Both **B7** and Roflumilast markedly reduced the percentage of goblet cell positivity in COPD rats (Figure 6A,C). Furthermore, the immunohistochemical staining of MUC5AC mucin expression in rat lung tissue revealed significantly elevated MUC5AC levels in the model group relative to the control group. Both **B7** and Roflumilast reduced MUC5AC mucin content in COPD rats (Figure 6B,D). These results demonstrate that **B7** ameliorates airway mucus secretion in COPD by reducing goblet cell positivity and decreasing MUC5AC mucin expression.

### 2.12. ***B7*** Regulated the cAMP-Medicated PKA CREB and NF-κB Pathway

In this study, we found that compound **B7** ameliorated COPD-associated pulmonary inflammation; however, its molecular mechanisms of action require further investigation. Increased intracellular cAMP levels activate protein kinase A (PKA), which suppresses inflammation in various cell types including monocytes, macrophages, and neutrophils [51,52]. Furthermore, studies indicate that PKA may inhibit NF-κB activation via the C-terminal transactivation domain of p65 [53]. NF-κB activation promotes its nuclear translocation and binding to homologous DNA sites, leading to the enhanced expression of inflammatory factors and exacerbated inflammatory responses [54]. Thus, the inhibition of NF-κB activation may alleviate inflammation-related symptoms.

**Figure 7:**
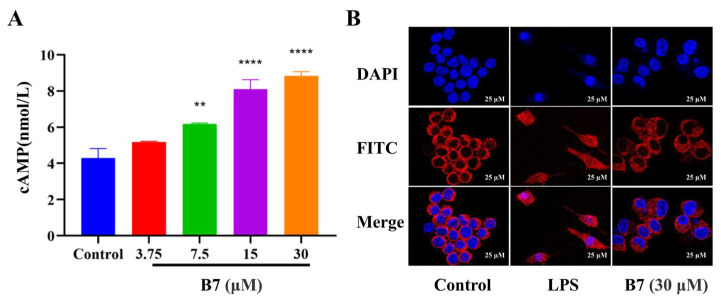
**B7** activates cAMP signaling and inhibits NF-κB nuclear translocation in RAW264.7 cells. (**A**) Intracellular cAMP levels in RAW264.7 cells after treatment with **B7** (3.75–30 μM) for 24 h. Data represent the mean ± SEM (*n* = 6). ** *p* < 0.01, **** *p* < 0.0001 vs. control group. (**B**) Immunofluorescence analysis of NF-κB nuclear translocation. Cells were stained with DAPI (blue, nuclei) and FITC-labeled anti-NF-κB antibody (red). Merge images show co-localization. Scale bar: 25 μm.

Intracellular cAMP levels were measured using ELISA. **B7** treatment dose-dependently increased cAMP levels, peaking at 30 μM (Figure 7A). The nuclear translocation of NF-κB was assessed through immunofluorescence staining (Figure 7B). For better visualization, FITC signals (normally green) were pseudo-colored red. In control cells, NF-κB was predominantly localized in the cytoplasm (red signal surrounding nuclei). LPS stimulation promoted NF-κB phosphorylation and nuclear translocation, increasing nuclear NF-κB levels (evidenced by red signal overlapping with blue nuclear staining). Compared with the LPS group, the **B7** treatment reduced nuclear NF-κB levels.

**Figure 8:**
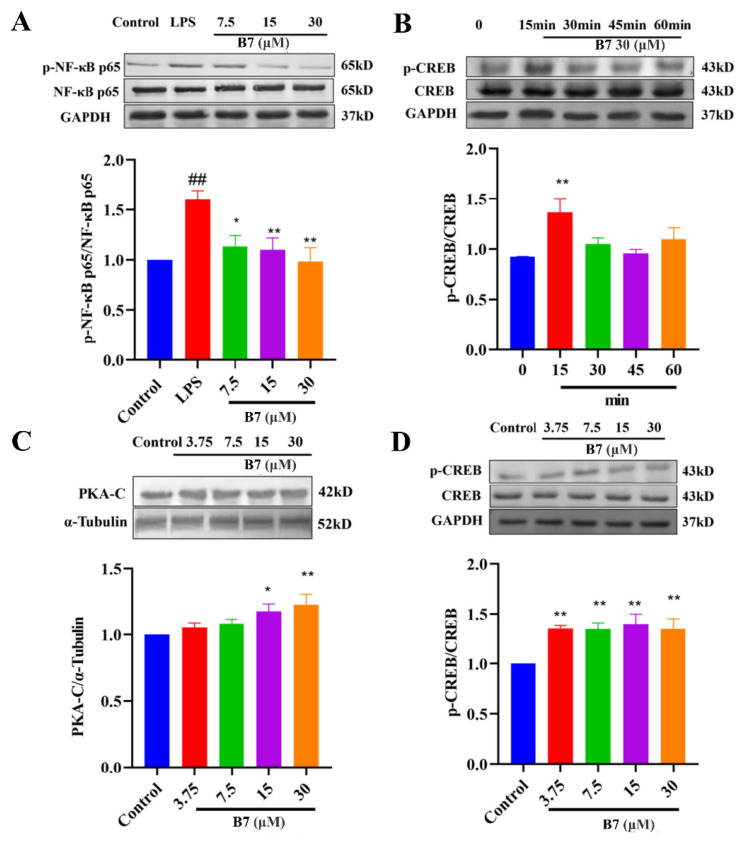
Western blot analysis was performed to evaluate the effects of **B7** on protein expression in RAW264.7 cells. (**A**) Expression levels of phospho-NF-κB p65 and total NF-κB p65 proteins. (**B**) Expression of phospho-CREB and total CREB proteins at different time points after treatment with 30 μM **B7**. (**C**) PKA-C protein expression after 15 min incubation with **B7**. (**D**) Expression of phospho-CREB and total CREB proteins after 15 min incubation with **B7**. Data represent the mean ± SEM (*n* = 6). ## *p* < 0.01 vs. control group; * *p* < 0.05, ** *p* < 0.01 vs. LPS group (**A**). * *p* < 0.05, ** *p* < 0.01 vs. control group (**B**,**D**).

To investigate the effects of **B7** on the cAMP-PKA-CREB and NF-κB signaling pathways in RAW264.7 cells, Western blotting was performed to detect the levels of relevant proteins. As shown in Figure 8A, **B7** treatment reduced the LPS-induced increase in p-NF-κB levels compared with the LPS group, consistent with the NF-κB nuclear translocation results obtained using immunofluorescence (IF). In Figure 8B, p-CREB levels peaked after 15 min of incubation with **B7**; therefore, this time point was selected for subsequent experiments. RAW264.7 cells were treated with different concentrations of **B7** (3.75, 7.5, 15, and 30 μM) for 15 min. **B7** significantly increased PKA-C expression and elevated p-CREB protein levels (Figure 8C,D). Combined with the cAMP measurement results, these data indicate that **B7** activates the cAMP-PKA-CREB signaling pathway. In conclusion, **B7** exerts its anti-inflammatory effects by activating the cAMP-PKA-CREB pathway and inhibiting the NF-κB pathway in RAW264.7 cells.

## 3. Materials and Methods

### 3.1. General Information on Chemistry

All commercial reagents and solvents utilized in the chemical synthesis were procured from the supplier without undergoing any processing, unless explicitly specified. The reaction progress was monitored via thin-layer chromatography (TLC) and visualized under ultraviolet light (254 or 365 nm). The melting point of the target compounds was determined using the WRS-2 melting point instrument. ^1^H and ^13^C nuclear magnetic resonance (NMR) spectra were obtained using JEOL ECS-400 spectrometers (Agilent Technologies, Santa Clara, CA, USA), and the high-resolution mass spectra of all the target compounds were obtained on a SYNAPT G2-Si spectrometer (Agilent Technologies, Santa Clara, CA, USA).

#### 3.1.1. 3-(Cyclopropylmethoxy)-4-(difluoromethoxy)-N-(3-guanidinopropyl)benzamide (**A1**)

To a stirred mixture of **1** (S-methylisothiourea hemisulfate, 2.78 g, 10 mmol) in water (35 mL), 1 N NaOH solution (100 mL) and saturated NaHCO_3_ solution (25 mL) were added, successively. After stirring for 5 min, benzyl chloroformate (4.65 mL, 30 mmol) was added over 10 min. The reaction mixture was stirred overnight at room temperature. The resulting mixture was diluted with saturated NaHCO_3_ solution and extracted with ethyl acetate. The organic phase was dried over anhydrous Na_2_SO_4_, filtered, and concentrated in vacuo to obtain compound **2** as a colorless oil. The crude product was used directly in the subsequent reaction without further purification.

To a solution of **2** (1.0 equiv) in dichloromethane, a solution of N-Boc-1,3-propanediamine (1.5 equiv) in DCM was added dropwise, followed by an appropriate amount of triethylamine. The reaction mixture was stirred for 5 h. The organic layer was collected, and the aqueous phase was extracted with DCM. The combined organic fractions were washed with brine and concentrated under reduced pressure to afford intermediate **3a** as a colorless oil, which was used directly in the next step without further purification.

Compound **3a** was dissolved in DCM and treated with TFA at 0 °C. The mixture was stirred for 2 h. After concentration in vacuo, the residue was neutralized with saturated aqueous NaHCO_3_, extracted with DCM, dried (Na_2_SO_4_), and concentrated to afford **4a**, used directly in the next step.

Compound **5a** (3-cyclopropylmethoxy-4-difluoromethoxy-benzoic acid, 1.0 equiv) was dissolved in anhydrous DCM. EDCI (1.5 equiv) and HOBt (1.5 equiv) were added successively. After stirring at room temperature for 10 min, a solution of **4a** (1.1 equiv) in DCM was added dropwise. The reaction mixture was stirred overnight, worked up (brine/DCM), and purified by column chromatography.

Compound **6a** (1.0 equiv) was dissolved in MeOH/THF (3:1). The solution was treated with Pd/C and AcOH (3.0 equiv) under H_2_ atmosphere for 2 h. After filtration, the filtrate was concentrated in vacuo and washed with Et_2_O to afford **A1** (86.2% yield). ^1^H NMR (400 MHz, Methanol-d_4_) δ 7.55 (d, *J* = 2.4 Hz, 1H), 7.44 (d, *J* = 7.6 Hz, 1H), 7.17 (d, *J* = 8.4 Hz, 1H), 6.83 (t, ^2^*J*_HF_ = 75.0 Hz, 1H), 3.95 (d, *J* = 6.8 Hz, 2H), 3.43 (t, *J* = 6.8 Hz, 2H), 3.24 (t, *J* = 6.8 Hz, 2H), 1.88 (s, 3H), 1.35–1.22 (m, 1H), 0.61 (d, *J* = 7.6 Hz, 2H), 0.37 (t, *J* = 5.0 Hz, 2H). ^13^C NMR (100 MHz, Methanol-d_4_) δ 167.92, 157.49, 150.30, 143.12, 132.27, 121.11, 119.94, 119.04, 116.46, 113.89, 113.33, 73.77, 38.73, 36.85, 28.57, 22.87, 9.69, 2.24. HRMS (ESI) calcd for C_16_H_22_F_2_N_4_O_3_ [M+H]^+^ 356.1660, found 356.1658.

#### 3.1.2. 3-(Cyclopentyloxy)-N-(3-guanidinopropyl)-4-methoxybenzamide (**A2**)

Compound **A2** was prepared as a white solid at 88.2% yield from N-Boc-1,3-propanediamine and 3-(cyclopentyloxy)-4-methoxybenzoic acid in a similar manner to that described for compound **A1**. ^1^H NMR (400 MHz, Methanol-d_4_) δ 7.48–7.39 (m, 2H), 6.97 (d, *J* = 8.4 Hz, 1H), 3.84 (s, 3H), 3.42 (t, *J* = 6.8 Hz, 2H), 3.23 (t, *J* = 6.8 Hz, 2H), 1.90 (d, *J* = 7.8 Hz, 2H), 1.88 (s, 3H), 1.87–1.78 (m, 6H), 1.66–1.57 (m, 2H). ^13^C NMR (100 MHz, Methanol-d_4_) δ 168.72, 157.50, 153.29, 147.26, 126.36, 120.47, 114.08, 111.11, 80.67, 55.15, 38.73, 36.74, 32.31, 28.74, 23.54, 22.91. HRMS (ESI) calcd for C_17_H_26_N_4_O_3_ [M+H]^+^ 334.2005, found 334.2001.

#### 3.1.3. N-(3-Guanidinopropyl)-3-isopropoxy-4-methoxybenzamide (**A3**)

Compound **A3** was prepared as a white solid at 85.2% yield from N-Boc-1,3-propanediamine and 3-isopropoxy-4-methoxybenzoic acid in a similar manner to that described for compound **A1**. ^1^H NMR (400 MHz, Methanol-d_4_) δ 7.51–7.40 (m, 2H), 6.99 (dd, *J* = 8.4, 1.4 Hz, 1H), 4.57 (dd, *J* = 6.0, 1.4 Hz, 1H), 3.85 (s, 3H), 3.42 (t, *J* = 6.8 Hz, 2H), 3.22 (t, *J* = 7.2 Hz, 2H), 1.88 (s, 3H), 1.84 (q, *J* = 6.8 Hz, 2H), 1.29 (dd, *J* = 6.2, 1.6 Hz, 6H). ^13^C NMR (100 MHz, Methanol-d_4_) δ 168.64, 157.53, 153.74, 146.89, 126.42, 121.10, 115.43, 111.27, 71.82, 55.11, 38.71, 36.75, 28.74, 22.95, 21.05. HRMS (ESI) calcd for C_15_H_24_N_4_O_3_ [M+H]^+^ 308.1848, found 308.1846.

#### 3.1.4. 3-Ethoxy-N-(3-guanidinopropyl)-4-methoxybenzamide (**A4**)

Compound **A4** was prepared as a white solid at 84.6% yield from N-Boc-1,3-propanediamine and 3-ethoxy-4-methoxybenzoic acid in a similar manner to that described for compound **A1**. ^1^H NMR (400 MHz, Methanol-d_4_) δ 7.47 (dd, *J* = 8.4, 2.0 Hz, 1H), 7.44 (d, *J* = 2.0 Hz, 1H), 6.98 (d, *J* = 8.0 Hz, 1H), 4.09 (q, *J* = 7.0 Hz, 2H), 3.86 (s, 3H), 3.43 (t, *J* = 6.8 Hz, 2H), 3.24 (t, *J* = 6.8 Hz, 2H), 1.88 (s, 3H), 1.86 (d, *J* = 6.8 Hz, 2H), 1.39 (t, *J* = 7.2 Hz, 3H). ^13^C NMR (100 MHz, Methanol-d_4_) δ 168.64, 157.47, 152.47, 148.13, 126.44, 120.67, 112.02, 110.87, 64.49, 55.16, 38.76, 36.73, 28.70, 22.89, 13.78. HRMS (ESI) calcd for C_14_H_22_N_4_O_3_ [M+H]^+^ 294.1692, found 294.1690.

#### 3.1.5. 3-(Cyclopropylmethoxy)-4-(difluoromethoxy)-N-(4-guanidinobutyl)benzamide (**A5**)

Compound **A5** was prepared as a white solid at 87.3% yield from *tert*-Butyl N-(4-aminobutyl)carbamate and 3-cyclopropylmethoxy-4-difluoromethoxy-benzoic acid in a similar manner to that described for compound **A1** [55]. ^1^H NMR (400 MHz, Methanol-d_4_) δ 7.51 (d, *J* = 2.0 Hz, 1H), 7.41 (dd, *J* = 8.4, 1.8 Hz, 1H), 7.18 (d, *J* = 8.0 Hz, 1H), 6.83 (t, ^2^*J*_HF_ = 75.0 Hz, 1H), 3.94 (d, *J* = 7.2 Hz, 2H), 3.39 (t, *J* = 6.0 Hz, 2H), 3.20 (t, *J* = 6.8 Hz, 2H), 1.88 (s, 3H), 1.69–1.60 (m, 4H), 1.33–1.29 (m, 1H), 0.65–0.59 (m, 2H), 0.37 (t, *J* = 5.0 Hz, 2H). ^13^C NMR (100 MHz, Methanol-d_4_) δ 167.82, 157.41, 150.33, 143.09, 132.45, 121.14, 119.78, 119.02, 116.45, 113.88, 113.26, 73.73, 40.75, 39.00, 26.42, 25.91, 22.69, 9.67, 2.23. HRMS (ESI) calcd for C_17_H_24_F_2_N_4_O_3_ [M+H]^+^ 370.1816, found 370.1813.

#### 3.1.6. 3-(Cyclopentyloxy)-N-(4-guanidinobutyl)-4-methoxybenzamide (**A6**)

Compound **A6** was prepared as a white solid at 85.0% yield from *tert*-Butyl N-(4-aminobutyl)carbamate and 3-(cyclopentyloxy)-4-methoxybenzoic acid in a similar manner to that described for compound **A1** [55]. ^1^H NMR (400 MHz, Methanol-d_4_) δ 7.47–7.36 (m, 2H), 6.97 (d, *J* = 8.4 Hz, 1H), 4.86–4.82 (m, 1H), 3.84 (s, 3H), 3.37 (t, *J* = 6.4Hz, 2H), 3.24–3.16 (m, 2H), 1.88 (s, 3H), 1.84–1.75 (m, 4H), 1.67–1.57 (m, 6H), 1.15 (td, *J* = 7.0, 1.6 Hz, 2H). ^13^C NMR (100 MHz, Methanol-d_4_) δ 178.67, 168.56, 157.43, 153.22, 147.25, 126.54, 120.39, 114.06, 111.11, 80.66, 65.57, 55.15, 40.77, 38.90, 32.32, 26.54, 25.94, 23.55, 22.62, 14.13. HRMS (ESI) calcd for C_18_H_28_N_4_O_3_ [M+H]^+^ 348.2161, found 348.2159.

#### 3.1.7. N-(4-Guanidinobutyl)-3-isopropoxy-4-methoxybenzamide (**A7**)

Compound **A7** was prepared as a white solid at 86.2% yield from *tert*-Butyl N-(4-aminobutyl)carbamate and 3-isopropoxy-4-methoxybenzoic acid in a similar manner to that described for compound **A1**. ^1^H NMR (400 MHz, Methanol-d_4_) δ 7.50–7.41 (m, 2H), 6.98 (d, *J* = 8.4 Hz, 1H), 4.62–4.52 (m, 1H), 3.84 (s, 3H), 3.43–3.34 (m, 2H), 3.20 (t, *J* = 6.8 Hz, 2H), 1.89 (s, 3H), 1.69–1.55 (m, 4H), 1.29 (d, *J* = 6.0, 6H). ^13^C NMR (100 MHz, Methanol-d_4_) δ 178.29, 168.44, 157.46, 153.65, 146.87, 126.59, 121.07, 115.38, 111.29, 71.80, 55.13, 40.77, 38.92, 26.53, 25.96, 22.39, 21.08. HRMS (ESI) calcd for C_16_H_26_N_4_O_3_ [M+H]^+^ 322.2005, found 322.2002.

#### 3.1.8. 3-Ethoxy-N-(4-guanidinobutyl)-4-methoxybenzamide (**A8**)

Compound **A8** was prepared as a white solid at 87.0% yield from *tert*-Butyl N-(4-aminobutyl)carbamate and 3-ethoxy-4-methoxybenzoic acid in a similar manner to that described for compound **A1**. ^1^H NMR (400 MHz, Methanol-d_4_) δ 7.47–7.39 (m, 2H), 6.97 (dd, *J* = 8.4, 1.8 Hz, 1H), 4.07 (qd, *J* = 7.0, 1.8 Hz, 2H), 3.85 (d, *J* = 1.8 Hz, 3H), 3.42–3.35 (m, 2H), 3.20 (dt, *J* = 6.8, 3.2 Hz, 2H), 1.91 (s, 3H), 1.69–1.60 (m, 4H), 1.39 (td, *J* = 7.0, 1.6 Hz, 3H). ^13^C NMR (100 MHz, Methanol-d_4_) δ 177.36, 168.54, 157.45, 152.45, 148.17, 126.62, 120.54, 112.01, 110.87, 64.45, 55.13, 40.76, 38.91, 26.53, 25.94, 21.75, 13.80. HRMS (ESI) calcd for C_15_H_24_N_4_O_3_ [M+H]^+^ 308.1848, found 308.1845.

#### 3.1.9. 1-(1-(3-(Cyclopropylmethoxy)-4-(difluoromethoxy)benzoyl)piperidin-4-yl)guanidine (**A9**)

A solution of compound **2** (650 mg, 1.81 mmol) in DCM was treated with a solution of **7** (4-amino-1-(tert-butoxycarbonyl)piperidine, 3.0 equiv) in DCM and triethylamine. After stirring for 5 h, the mixture was washed with brine, extracted with DCM, dried and concentrated to afford **8** as a colorless oil. Compound **8** was dissolved in DCM and treated with an equal volume of TFA in an ice-water bath. After completion of Boc deprotection, the mixture was neutralized with saturated aqueous NaHCO_3_, extracted with DCM and concentrated to afford intermediate **9**.

3-(Cyclopropylmethoxy)-4-(difluoromethoxy)benzoic acid (258 mg, 1.0 mmol) was dissolved in anhydrous DCM and cooled in an ice-water bath. Oxalyl chloride (1.5 equiv) and DMF (3–5 drops) were added. The mixture was stirred for 30 min in an ice-water bath, then 5 h at room temperature. A solution of **9** (1.0 mmol) in DCM was added dropwise, followed by triethylamine. After 2 h, the mixture was purified through column chromatography to afford **10**.

Compound **10** was dissolved in MeOH/THF and treated with Pd/C and AcOH (3.0 equiv) under H_2_ atmosphere for Cbz deprotection. After completion, the mixture was filtered, concentrated in vacuo, and purified to afford **A9** as a white solid (86.9% yield). ^1^H NMR (400 MHz, Methanol-d_4_) δ 7.19 (d, *J* = 8.0 Hz, 1H), 7.11 (s, 1H), 6.99 (d, *J* = 3.2 Hz, 1H), 6.81 (t, ^2^*J*_HF_ = 76.0 Hz, 1H), 4.49 (s, 1H), 3.92 (d, *J* = 6.8 Hz, 2H), 3.79–3.65 (m, 2H), 3.14 (d, *J* = 60.4 Hz, 2H), 2.02 (s, 2H), 1.91 (s, 6H), 1.52 (s, 2H), 1.28 (q, *J* = 6.6 Hz, 1H), 0.61 (d, *J* = 7.6 Hz, 2H), 0.36 (d, *J* = 5.2 Hz, 2H). ^13^C NMR (100 MHz, Methanol-d_4_) δ 170.17, 156.54, 150.70, 141.66, 133.78, 121.75, 119.17, 116.54, 113.97, 113.04, 73.73, 31.08, 22.65, 9.65, 2.24. HRMS (ESI) calcd for C_18_H_24_F_2_N_4_O_3_ [M+H]^+^ 382.1816, found 382.1813.

#### 3.1.10. 2-Guanidinoethyl 3-(cyclopropylmethoxy)-4-(difluoromethoxy)benzoate (**B1**)

To a solution of **2** (1.0 equiv) in DCM, a solution of monoethanolamine (3.0 equiv) in DCM and an appropriate amount of triethylamine were added. After 5 h, the mixture was washed with brine, extracted with DCM, dried and concentrated to give **11a**.

Compound **5a**, 3-cyclopropylmethoxy-4-difluoromethoxy-benzoic acid (300 mg, 1.61 mmol), was dissolved in anhydrous DCM. To this solution were added successively EDCI (1.5 equiv) and DMAP (0.5 equiv). After 10 min of stirring, a solution of **11a** (1.1 equiv) in DCM was added dropwise. The resulting mixture was stirred overnight, concentrated, and purified through column chromatography to afford **12a**.

A solution of **12a** (1.0 equiv) in MeOH/THF (3:1) was treated with Pd/C and AcOH (3 equiv) under H_2_ atmosphere. After 2 h, the mixture was filtered, concentrated, and washed with Et_2_O to give **B1** (82.7% yield). ^1^H NMR (400 MHz, Methanol-d_4_) δ 7.70–7.60 (m, 2H), 7.21 (d, *J* = 8.4 Hz, 1H), 6.88 (t, ^2^*J*_HF_ = 76.0 Hz, 1H), 4.41 (t, *J* = 5.2 Hz, 2H), 3.93 (dd, *J* = 7.2, 1.6 Hz, 2H), 3.59 (t, *J* = 5.2 Hz, 2H), 1.85 (s, 3H), 1.33–1.22 (m, 1H), 0.62 (d, *J* = 4.2 Hz, 2H), 0.37 (d, *J* = 5.0 Hz, 2H). ^13^C NMR (100 MHz, Methanol-d_4_) δ 179.26, 165.52, 157.90, 150.21, 144.61, 127.63, 122.67, 120.88, 118.91, 116.33, 115.03, 113.76, 73.72, 63.02, 60.11, 43.71, 40.05, 22.95, 9.65, 2.27. HRMS (ESI) calcd for C_15_H_19_F_2_N_3_O_4_ [M+H]^+^ 343.1344, found 343.1340.

#### 3.1.11. 2-Guanidinoethyl-3-ethoxy-4-methoxybenzoate (**B2**)

Compound **B2** was prepared as a white solid at 85.4% yield from monoethanolamine and 3-ethoxy-4-methoxybenzoic acid in a similar manner to that described for compound **B1**. ^1^H NMR (400 MHz, Methanol-d_4_) δ 7.70–7.64 (m, 1H), 7.53 (d, *J* = 2.0 Hz, 1H), 7.00 (dd, *J* = 8.5, 1.6 Hz, 1H), 4.38 (t, *J* = 5.2 Hz, 2H), 4.06 (dd, *J* = 6.8, 1.6 Hz, 2H), 3.87 (s, 3H), 3.58 (t, *J* = 5.2 Hz, 2H), 1.93–1.90 (m, 6H), 1.39 (t, *J* = 7.2 Hz, 3H). ^13^C NMR (100 MHz, Methanol-d_4_) δ 176.52, 166.32, 157.90, 154.04, 148.14, 123.77, 121.87, 113.58, 110.75, 64.45, 62.61, 55.15, 40.16, 21.16, 13.74. HRMS (ESI) calcd for C_13_H_19_N_3_O_4_ [M+H]^+^ 281.1376, found 281.1373.

#### 3.1.12. 3-Guanidinopropyl-3-(cyclopropylmethoxy)-4-(difluoromethoxy)benzoate (**B3**)

Compound **B3** was prepared as a white solid at 85.9% yield from 3-aminopropanol and 3-cyclopropylmethoxy-4-difluoromethoxy-benzoic acid in a similar manner to that described for compound **B1**. ^1^H NMR (400 MHz, Methanol-d_4_) δ 7.66–7.59 (m, 2H), 7.20 (d, *J* = 8.6 Hz, 1H), 6.87 (t, ^2^*J*_HF_ = 74.8 Hz, 1H), 4.37 (t, *J* = 6.2 Hz, 2H), 3.93 (d, *J* = 6.8 Hz, 2H), 3.33 (t, *J* = 6.8 Hz, 2H), 2.04 (p, *J* = 6.6 Hz, 2H), 1.87 (s, 3H), 1.27 (dt, *J* = 12.2, 7.2 Hz, 1H), 0.66–0.59 (m, 2H), 0.38 (t, *J* = 5.0 Hz, 2H). ^13^C NMR (100 MHz, Methanol-d_4_) δ 165.72, 157.59, 150.20, 144.56, 127.96, 122.54, 122.43, 120.89, 118.93, 116.36, 114.99, 114.90, 113.78, 73.74, 62.14, 58.35, 38.14, 38.05, 31.15, 27.83, 22.94, 9.65, 2.25. HRMS (ESI) calcd for C_16_H_21_F_2_N_3_O_4_ [M+H]^+^ 357.1500, found 357.1498.

#### 3.1.13. 3-Guanidinopropyl-3-isopropoxy-4-methoxybenzoate (**B4**)

Compound **B4** was prepared as a white solid at 86.2% yield from 3-aminopropanol and 3-isopropoxy-4-methoxybenzoic acid in a similar manner to that described for compound **B1**. ^1^H NMR (400 MHz, Methanol-d_4_) δ 7.66 (dt, *J* = 8.6, 1.8 Hz, 1H), 7.52 (t, *J* = 1.8 Hz, 1H), 7.01 (dd, *J* = 8.6, 1.4 Hz, 1H), 4.55 (m, 1H), 4.35 (td, *J* = 6.2, 1.4 Hz, 2H), 3.87 (d, *J* = 1.4 Hz, 3H), 3.33 (td, *J* = 6.8, 1.4 Hz, 2H), 2.03 (m, 2H), 1.87 (s, 3H), 1.29 (dd, *J* = 6.0, 1.5 Hz, 6H). ^13^C NMR (100 MHz, Methanol-d_4_) δ 179.07, 166.50, 157.58, 155.14, 146.79, 124.07, 122.20, 116.99, 111.19, 71.81, 61.67, 55.14, 38.09, 27.92, 22.85, 20.96. HRMS (ESI) calcd for C_15_H_23_N_3_O_4_ [M+H]^+^ 309.1689, found 309.1686.

#### 3.1.14. 3-Guanidinopropyl-3-(cyclopentyloxy)-4-methoxybenzoate (**B5**)

Compound **B5** was prepared as a white solid at 87.7% yield from 3-aminopropanol and 3-(cyclopentyloxy)-4-methoxybenzoic acid in a similar manner to that described for compound **B1**. ^1^H NMR (400 MHz, Methanol-d_4_) δ 7.66–7.61 (m, 1H), 7.49 (d, *J* = 2.0 Hz, 1H), 6.98 (dd, *J* = 8.4, 1.6 Hz, 1H), 4.81 (q, *J* = 3.0 Hz, 1H), 4.34 (t, *J* = 6.4 Hz, 2H), 3.85 (s, 3H), 3.32 (t, *J* = 7.2, 2H), 2.08–1.99 (m, 2H), 1.94–1.88 (m, 2H), 1.87 (s, 3H), 1.85–1.76 (m, 4H), 1.68–1.58 (m, 2H). ^13^C NMR (100 MHz, Methanol-d_4_) δ 166.57, 157.59, 154.65, 147.16, 123.47, 122.11, 115.44, 110.99, 80.63, 61.65, 55.16, 38.09, 32.31, 27.94, 23.58, 22.92. HRMS (ESI) calcd for C_17_H_25_N_3_O_4_ [M+H]^+^ 335.1845, found 335.1842.

#### 3.1.15. 3-Guanidinopropyl-3-ethoxy-4-methoxybenzoate (**B6**)

Compound **B6** was prepared as a white solid at 88.1% yield from 3-aminopropanol and 3-ethoxy-4-methoxybenzoic acid in a similar manner to that described for compound **B1**. ^1^H NMR (400 MHz, Methanol-d_4_) δ 7.69–7.61 (m, 1H), 7.50 (d, *J* = 2.0 Hz, 1H), 6.99 (dd, *J* = 8.6, 1.6 Hz, 1H), 4.34 (td, *J* = 6.2, 1.8 Hz, 2H), 4.10–4.02 (m, 2H), 3.87 (s, 3H), 3.32 (td, *J* = 6.8 Hz, 2H), 2.08–1.97 (m, 2H), 1.87 (s, 3H), 1.39 (td, *J* = 7.0, 1.8 Hz, 3H). ^13^C NMR (100 MHz, Methanol-d_4_) δ 179.20, 166.53, 157.59, 153.93, 148.12, 123.60, 122.21, 113.52, 110.76, 64.45, 61.68, 55.15, 38.09, 27.92, 22.92, 13.74. HRMS (ESI) calcd for C_14_H_21_N_3_O_4_ [M+H]^+^ 295.1532, found 295.1530.

#### 3.1.16. 4-Guanidinobutyl 3-(cyclopropylmethoxy)-4-(difluoromethoxy)benzoate (**B7**)

Compound **B7** was prepared as a white solid at 86.1% yield from 4-amino-1-butanol and 3-ethoxy-4-methoxybenzoic acid in a similar manner to that described for compound B1. ^1^H NMR (400 MHz, Methanol-d_4_) δ 7.64–7.58 (m, 2H), 7.20 (d, *J* = 8.2 Hz, 1H), 6.87 (t, ^2^*J*_HF_ = 74.8 Hz, 1H), 4.33 (t, *J* = 6.4 Hz, 2H), 3.92 (d, *J* = 7.2 Hz, 2H), 3.22 (t, *J* = 7.0 Hz, 2H), 1.87 (s, 3H), 1.86–1.76 (m, 2H), 1.76–1.65 (m, 2H), 1.31–1.21 (m, 1H), 0.67–0.59 (m, 2H), 0.40–0.33 (m, 2H). ^13^C NMR (100 MHz, Methanol-d_4_) δ 165.75, 157.49, 150.19, 144.48, 128.13, 122.40, 120.89, 116.37, 114.88, 73.70, 64.43, 48.31, 48.10, 47.89, 47.68, 47.46, 47.25, 47.04, 40.67, 25.69, 25.28, 22.87, 9.65, 2.26. HRMS (ESI) Calcd. for C_19_H_27_F_2_N_3_O_6_^+^ [M+H]^+^: 372.1725, found: 372.1729.

#### 3.1.17. 2-Guanidinoethyl 2-(3-ethoxy-4-methoxyphenyl)acetate (**C1**)

To a solution of **13** (1,3-bis(*tert*-butoxycarbonyl)-2-methyl-2-thiopseudourea, 1.0 equiv) in DCM, a solution of ethanolamine (1.2 equiv) in DCM was added. After stirring for 6–8 h, the mixture was washed with brine, extracted with DCM, dried (Na_2_SO_4_), and concentrated to afford **14** as a white solid.

2-(3-Ethoxy-4-methoxyphenyl)acetic acid (210 mg, 1.0 mmol) was dissolved in DCM. To this solution were added successively EDCI (1.5 equiv) and DMAP (0.5 equiv). After 10 min, a solution of **14** (1.1 equiv) in DCM was added dropwise. The mixture was stirred overnight, washed with brine, extracted with DCM, dried (Na_2_SO_4_), and concentrated. The residue was purified through column chromatography to afford **15a**.

A solution of **15a** was treated with TFA. After completion, the mixture was concentrated. The residue was dissolved in 0.5 N HCl (3–5 equiv HCl), sonicated, stirred, and lyophilized to afford **C1** as a white solid powder (83.7% yield). ^1^H NMR (400 MHz, DMSO-d_6_) δ 12.24 (s, 1H), 7.68 (t, *J* = 5.8 Hz, 1H), 6.88–6.84 (m, 2H), 6.74 (dd, *J* = 8.2, 2.0 Hz, 1H), 5.08 (t, *J* = 5.2 Hz, 1H), 3.97 (q, *J* = 7.0 Hz, 2H), 3.73 (s, 3H), 3.51–3.45 (m, 4H), 3.19 (q, *J* = 5.4 Hz, 2H), 1.31 (t, *J* = 6.8 Hz, 3H). ^13^C NMR (100 MHz, DMSO-d_6_) δ 173.40, 158.05, 149.19, 147.27, 127.94, 121.90, 113.87, 113.44, 64.24, 59.91, 55.95, 43.94, 15.32. HRMS (ESI) calcd for C_14_H_21_N_3_O_4_ [M+H]^+^ 295.1532, found 295.1533.

#### 3.1.18. 2-Guanidinoethyl-2-(3-bromo-4-methoxyphenyl)acetate (**C2**)

Compound **C2** was prepared as a white solid at 86.1% yield from 3-bromo-4-methoxyphenylacetic acid in a similar manner described for compound **C1**. ^1^H NMR (400 MHz, DMSO-d_6_) δ 7.58 (t, *J* = 5.6 Hz, 1H), 7.43 (d, *J* = 2.2 Hz, 1H), 7.19 (dd, *J* = 8.4, 2.2 Hz, 1H), 7.01 (d, *J* = 8.4 Hz, 1H), 5.04 (t, *J* = 5.2 Hz, 1H), 3.78 (s, 3H), 3.49 (s, 2H), 3.44 (t, *J* = 5.0 Hz, 2H), 3.14 (q, *J* = 5.4 Hz, 2H). ^13^C NMR (100 MHz, DMSO-d_6_) δ 173.15, 158.02, 154.67, 134.25, 130.41, 129.31, 112.97, 110.65, 59.90, 56.72, 43.94. HRMS (ESI) calcd for C_12_H_16_BrN_3_O_3_ [M+H]^+^ 329.0375, found 329.0372.

#### 3.1.19. 2-(3-Ethoxy-4-methoxyphenyl)-N-(2-guanidinoethyl)acetamide (**C3**)

Compound **C3** was prepared as a white solid at 81.5% yield from 4-ethoxy-3-methoxyphenylacetic acid and ethylenediamine in a similar manner to that described for compound **C1**. ^1^H NMR (400 MHz, DMSO-d_6_) δ 8.24–8.18 (m, 1H), 7.73 (d, *J* = 5.6 Hz, 1H), 6.84 (d, *J* = 2.0 Hz, 1H), 6.81 (d, *J* = 8.2 Hz, 1H), 6.71 (dd, *J* = 8.2, 2.0 Hz, 1H), 3.92 (q, *J* = 7.0 Hz, 2H), 3.69 (s, 3H), 3.32 (s, 2H), 3.18–3.10 (m, 4H), 1.26 (t, *J* = 7.0 Hz, 3H). ^13^C NMR (100 MHz, DMSO-d_6_) δ 171.63, 157.88, 149.19, 147.14, 129.09, 121.61, 113.68, 113.52, 64.25, 56.02, 42.44, 38.62, 15.35. HRMS (ESI) calcd for C_14_H_22_N_4_O_3_ [M+H]^+^ 294.1692, found 294.1690.

#### 3.1.20. 3-Ethoxy-N-(2-guanidinoethyl)-4-methoxybenzamide (**C4**)

Compound **C4** was prepared as a white solid at 67.4% yield from 3-ethoxy-4-methoxybenzoic acid and ethylenediamine in a similar manner to that described for compound **C1**. ^1^H NMR (400 MHz, DMSO-d_6_) δ 8.67 (t, *J* = 5.4 Hz, 1H), 7.87 (t, *J* = 5.6 Hz, 1H), 7.49 (d, *J* = 8.4 Hz, 2H), 6.97 (d, *J* = 8.2 Hz, 1H), 4.03 (q, *J* = 6.8 Hz, 2H), 3.76 (s, 3H), 3.39–3.24 (m, 4H), 1.30 (t, *J* = 6.8 Hz, 3H). ^13^C NMR (100 MHz, DMSO-d_6_) δ 166.74, 157.90, 151.95, 147.92, 126.79, 121.11, 112.23, 111.55, 64.41, 56.09, 48.37, 15.26. HRMS (ESI) calcd for C_13_H_20_N_4_O_3_ [M+H]^+^ 280.1535, found 280.1533.

##### Genereal Procedure for Compounds **D1**–**D5**

A solution of 3-(cyclopropylmethoxy)-4-(difluoromethoxy) benzoic acid (1.0 equiv) in anhydrous dichloromethane was treated with EDCI (1.5 equiv) and DMAP (0.5 equiv). The mixture was stirred at room temperature for 20 min. A solution of the appropriate alcohol (**18a** or **18b**) or amine (**19a** or **19b** or **19c**) (1.0 equiv) in dichloromethane was then added, and stirring was continued at room temperature overnight. The reaction was monitored through TLC and found to be essentially complete. The mixture was washed with saturated aqueous NaCl solution and extracted with dichloromethane. The combined organic layers were dried (over anhydrous Na_2_SO_4_). Purification through column chromatography afforded the desired products **D1**–**D5**.

#### 3.1.21. 4-Cyanophenyl-3-(cyclopropylmethoxy)-4-(difluoromethoxy)benzoate (**D1**)

Yield: 84.5%; ^1^H NMR (400 MHz, Chloroform-d) δ 7.81 (dd, *J* = 8.4, 2.0 Hz, 1H), 7.76 (d, *J* = 2.0 Hz, 1H), 7.74 (d, *J* = 2.0 Hz, 1H), 7.72 (d, *J* = 2.0 Hz, 1H), 7.36 (d, *J* = 2.0 Hz, 1H), 7.35 (d, *J* = 2.0 Hz, 1H), 7.29 (d, *J* = 8.4 Hz, 1H), 6.77 (t, ^2^*J*_HF_ = 74.8 Hz, 1H), 3.97 (d, *J* = 7.2 Hz, 2H), 1.36–1.29 (m, 1H), 0.73–0.65 (m, 2H), 0.43–0.35 (m, 2H). ^13^C NMR (100 MHz, Chloroform-d) δ 163.58, 154.18, 150.50, 145.13, 145.09, 145.06, 133.87, 126.65, 123.79, 122.99, 122.05, 118.28, 115.77, 113.11, 110.11, 74.33, 10.09, 3.37. HRMS (ESI) calcd for C_19_H_15_F_2_NO_4_ [M+H]^+^ 359.0969, found 359.0965.

#### 3.1.22. N-(4-Cyano-1H-pyrazol-3-yl)-3-(cyclopropylmethoxy)-4-(difluoromethoxy)benzamide (**D2**)

Yield: 86.1%; ^1^H NMR (400 MHz, Chloroform-d) δ 7.73 (dd, *J* = 8.4, 2.0 Hz, 1H), 7.67 (d, *J* = 2.0 Hz, 1H), 7.59 (s, 1H), 6.73 (t, ^2^*J*_HF_ = 76.0 Hz, 1H), 6.61 (s, 1H), 3.92 (d, *J* = 7.2 Hz, 2H), 1.34–1.27 (m, 1H), 0.71–0.60 (m, 2H), 0.41–0.31 (m, 2H). ^13^C NMR (100 MHz, Chloroform-d) δ 168.97, 156.12, 149.85, 144.59, 143.31, 129.14, 125.26, 121.39, 118.37, 117.28, 115.76, 113.16, 113.02, 75.17, 74.29, 10.08, 3.37. HRMS (ESI) calcd for C_16_H_14_F_2_N_4_O_3_ [M+H]^+^ 348.1034, found 348.1031.

#### 3.1.23. 3-(Cyclopropylmethoxy)-4-(difluoromethoxy)-N-(3,5-difluorophenyl)benzamide (**D3**)

Yield: 81.7%; ^1^H NMR (400 MHz, Methanol-d_4_) δ 7.59 (d, *J* = 2.0 Hz, 1H), 7.54–7.47 (m, 1H), 7.44–7.35 (m, 2H), 7.22 (d, *J* = 8.3 Hz, 1H), 7.07–6.63 (m, 2H), 3.96 (d, *J* = 7.2 Hz, 2H), 1.35–1.26 (m, 1H), 0.68–0.59 (m, 2H), 0.42–0.33 (m, 2H). ^13^C NMR (100 MHz, Methanol-d_4_) δ 166.31, 164.50, 164.34, 162.06, 161.92, 150.40, 143.53, 141.34, 132.47, 121.09, 120.26, 118.99, 116.42, 113.62, 113.62, 103.28, 102.99, 98.95, 98.69, 98.43, 73.79, 9.66, 2.23. HRMS (ESI) calcd for C_18_H_15_F_4_NO_3_ [M+H]^+^ 369.0988, found 369.0982.

#### 3.1.24. (2,5-Dioxopyrrolidin-1-yl)methyl-3-(cyclopropylmethoxy)-4-(difluoromethoxy)benzoate (**D4**)

Yield: 89.3%; ^1^H NMR (400 MHz, Chloroform-d) δ 7.60–7.54 (m, 2H), 7.16 (d, *J* = 8.2 Hz, 1H), 6.69 (t, ^2^*J*_HF_ = 74.8 Hz, 1H), 5.74 (s, 2H), 3.90 (d, *J* = 7.2 Hz, 2H), 2.81 (s, 4H), 1.32–1.26 (m, 1H), 0.69–0.61 (m, 2H), 0.38–0.32 (m, 2H). ^13^C NMR (100 MHz, Chloroform-d) δ 175.65, 164.49, 150.25, 144.62, 144.58, 127.06, 123.33, 121.81, 118.38, 115.79, 115.54, 113.19, 74.24, 61.73, 28.24, 10.08, 3.31. HRMS (ESI) calcd for C_17_H_17_F_2_NO_6_ [M+H]^+^ 369.1024, found 369.1022.

#### 3.1.25. 3-(Cyclopropylmethoxy)-N-(3,5-difluoro-4-methoxyphenyl)-4-(difluoromethoxy)benzamide (**D5**)

Yield: 79.7%; ^1^H NMR (400 MHz, Chloroform-d) δ 7.83 (s, 1H), 7.47 (d, *J* = 2.4 Hz, 1H), 7.29–7.25 (m, 2H), 7.25 (d, *J* = 3.0 Hz, 1H), 7.19 (d, *J* = 8.0 Hz, 1H), 6.70 (t, ^2^*J*_HF_ = 74.8 Hz, 1H), 3.96 (s, 3H), 3.91 (d, *J* = 7.0 Hz, 2H), 1.33–1.23 (m, 1H), 0.69–0.61 (m, 2H), 0.35 (dt, *J* = 6.1, 4.7 Hz, 2H). ^13^C NMR (100 MHz, Chloroform-d) δ 165.05, 157.03, 156.96, 154.57, 154.50, 150.88, 143.31, 132.84, 132.51, 122.16, 118.95, 118.40, 115.80, 113.75, 113.20, 104.89, 104.61, 74.20, 62.13, 10.04, 3.31. HRMS (ESI) calcd for C_19_H_17_F_4_NO_4_ [M+H]^+^ 399.1094, found 399.1090.

##### Genereal Procedure for Compounds **D6**–**D8**

A solution of 4-(difluoromethoxy)-3-(cyclopropylmethoxy)benzoic acid (1.0 equiv) in anhydrous dichloromethane was treated sequentially with EDCI (1.5 equiv) and DMAP (0.5 equiv). The mixture was stirred at room temperature for 15 min. A solution of **20** (**21** or **22**) (1.0 equiv) in dichloromethane was then added, and stirring was continued at room temperature overnight. The mixture was washed with saturated aqueous NaCl solution and extracted with dichloromethane. The combined organic layers were dried (over anhydrous Na_2_SO_4_). Purification through column chromatography afforded an intermediate as a colorless oil.

This intermediate oil was dissolved in formic acid and stirred at room temperature for 7 h. The formic acid was removed in vacuo. The residue was co-evaporated with dichloromethane (3–5 times) to ensure the complete removal of formic acid. Petroleum ether was then added to the residue, and the mixture was stirred for 2 h. The resulting solid was collected through filtration, yielding the desired products **D6**–**D8**.

#### 3.1.26. (3-(Cyclopropylmethoxy)-4-(difluoromethoxy)benzoyl)glycine (**D6**)

Yield: 87.9%; ^1^H NMR (400 MHz, Methanol-d_4_) δ 7.54 (d, *J* = 2.0 Hz, 1H), 7.46–7.40 (m, 1H), 7.18 (d, *J* = 8.0 Hz, 1H), 6.83 (t, ^2^*J*_HF_ = 75.2 Hz, 1H), 4.05 (d, *J* = 1.6 Hz, 2H), 3.93 (dd, *J* = 7.0, 1.6 Hz, 2H), 1.32–1.26 (m, 1H), 0.65–0.58 (m, 2H), 0.40–0.32 (m, 2H). ^13^C NMR (100 MHz, Methanol-d_4_) δ 172.01, 168.01, 150.30, 143.27, 131.92, 121.09, 119.93, 119.04, 116.47, 113.90, 113.34, 73.70, 41.10, 9.65, 2.22. HRMS (ESI) calcd for C_14_H_15_F_2_NO_5_ [M+H]^+^ 315.0918, found 315.0916.

#### 3.1.27. 2-((3-(Cyclopropylmethoxy)-4-(difluoromethoxy)benzoyl)oxy)acetic Acid (**D7**)

Yield: 84.7%; ^1^H NMR (400 MHz, Methanol-d_4_) δ 7.72–7.61 (m, 2H), 7.20 (dd, *J* = 8.0, 2.0 Hz, 1H), 6.88 (td, ^2^*J*_HF_ = 75.2, 2.0 Hz 1H), 4.81 (d, *J* = 2.0 Hz, 2H), 3.93 (dd, *J* = 6.8, 2.0 Hz, 2H), 1.31–1.24 (m, 1H), 0.67–0.56 (m, 2H), 0.42–0.31 (m, 2H). ^13^C NMR (100 MHz, Methanol-d_4_) δ 170.01, 165.28, 150.16, 144.73, 127.39, 122.79, 120.82, 118.95, 116.37, 115.12, 113.80, 73.75, 60.97, 9.63, 2.25. HRMS (ESI) calcd for C_14_H_14_F_2_O_6_ [M+H]^+^ 316.0758, found 316.0755.

#### 3.1.28. 1-(3-(Cyclopropylmethoxy)-4-(difluoromethoxy)benzoyl)piperidine-4-carboxylic Acid (**D8**)

Yield: 81.7%; ^1^H NMR (400 MHz, Methanol-d_4_) δ 7.19 (d, *J* = 8.4 Hz, 1H), 7.09 (d, *J* = 1.6 Hz, 1H), 6.99–6.61 (td, ^2^*J*_HF_ = 76.0 Hz, 1H), 6.94 (d, *J* = 12 Hz, 1H), 4.41 (s, 1H), 3.91 (dd, *J* = 6.8, 1.2 Hz, 2H), 3.79–3.59 (m, 1H), 3.11 (d, *J* = 43.0 Hz, 2H), 2.61 (td, *J* = 10.6, 5.2 Hz, 1H), 1.93 (d, *J* = 54.5 Hz, 2H), 1.65 (s, 2H), 1.34–1.20 (m, 1H), 0.62 (t, *J* = 6.8 Hz, 2H), 0.36 (d, *J* = 5.0 Hz, 2H). ^13^C NMR (100 MHz, Methanol-d_4_) δ 176.50, 170.16, 150.70, 141.59, 134.02, 121.75, 119.13, 119.10, 116.56, 113.99, 112.96, 73.70, 41.47, 40.46, 28.41, 27.68, 9.64, 2.22. HRMS (ESI) calcd for C_18_H_21_F_2_NO_5_ [M+H]^+^ 369.1388, found 369.1384.

#### 3.1.29. *tert*-Butyl (R)-3-(2-Amino-3-((2,6-dichloroisonicotinoyl)oxy)propyl)-1H-indole-1-carboxylate (**26**)

To a solution of **23** (Fmoc-L-Trp(Boc)-OH, 527 mg, 1.0 mmol) in DME (20 mL) at −15 °C was added N-methylmorpholine (NMM, 1.1 equiv), followed by isobutyl chloroformate (IBCF, 1.1 equiv). After stirring for 40 min at −15 °C, aqueous NaBH_4_ (5.0 equiv) was added dropwise. The reaction was stirred for 30 min, quenched with saturated NaHCO_3_(aq), and extracted with EtOAc (3 × 20 mL). The combined organic phases were concentrated in vacuo to afford **24** as a white solid (505 mg, 98.5%), used directly in the next step.

To a solution of 2,6-dichloroisonicotinic acid (97.92 mg, 0.51 mmol) in anhydrous DCM at 0 °C, oxalyl chloride (1.5 equiv) and catalytic DMF (3 drops) were added. After stirring at 0 °C for 30 min, the mixture was warmed to rt and stirred for 6 h. Volatiles were removed in vacuo, and the residue was dissolved in anhydrous DCM. To this solution, a solution of **24** (263 mg, 1.0 equiv) in anhydrous DCM was added dropwise, followed by triethylamine. After 30 min, the reaction was washed with brine, extracted with DCM, dried (Na_2_SO_4_), and concentrated. Purification through column chromatography (PE/EA 2:1) afforded 25 as a white solid (302 mg, 86.3%).

To a solution of **25** (258 mg, 1.0 equiv) in DMF at rt, piperidine (4.0 equiv) was added dropwise. After 20 min, the mixture was diluted with brine and extracted with DCM (3 × 15 mL). The combined organic layers were dried (Na_2_SO_4_), concentrated, and purified through column chromatography (PE/EA 2:1) to afford **26** as a white solid (130 mg, 74.6%). ^1^H NMR (400 MHz, DMSO-d_6_) δ 8.67 (d, *J* = 8.0 Hz, 1H), 7.99 (d, *J* = 8.2 Hz, 1H), 7.78 (s, 2H), 7.66 (dt, *J* = 7.6, 1.0 Hz, 1H), 7.44 (s, 1H), 7.30–7.16 (m, 2H), 4.90 (t, *J* = 5.8 Hz, 1H), 4.20 (td, *J* = 8.0, 5.4 Hz, 1H), 3.50 (m, 2H), 3.05–2.78 (m, 2H), 1.54 (s, 9H). ^13^C NMR (100 MHz, DMSO-d_6_) δ 162.57, 150.29, 149.52, 148.44, 135.29, 131.02, 124.82, 124.10, 122.96, 122.06, 119.79, 117.98, 115.21, 83.89, 63.08, 52.82, 28.17, 26.18.

#### 3.1.30. (R)-2-(3-(Cyclopropylmethoxy)-4-(difluoromethoxy)benzamido)-3-(1H-indol-3-yl)propyl 2,6-dichloroisonicotinate (**E1**)

To a solution of 4-(difluoromethoxy)-3-(cyclopropylmethoxy)benzoic acid (130 mg, 0.5 mmol) in DCM, EDCI (1.5 equiv) and DMAP (0.5 equiv) were added sequentially. A solution of intermediate **26** in DCM was then added. After stirring at rt for 10 h (TLC completion), the mixture was washed with brine, extracted with DCM, dried (Na_2_SO_4_), and concentrated. Purification through column chromatography (PE/EA 2:1) afforded **27** as a white solid (257 mg, 83.3%).

To a solution of **27** in DCM at 0 °C, an equal volume of TFA was added dropwise. After 35 min, volatiles were removed in vacuo. The residue was neutralized with saturated NaHCO_3_ (aq), extracted with DCM (3 × 10 mL), dried (Na_2_SO_4_), and concentrated to afford E1 as a white solid (162 mg, 87.7%). ^1^H NMR (400 MHz, Chloroform-d) δ 8.19 (s, 1H), 7.69 (d, *J* = 8.0 Hz, 1H), 7.64–7.54 (m, 2H), 7.41 (d, *J* = 8.0 Hz, 1H), 7.38 (s, 2H), 7.23–7.15 (m, 2H), 7.12 (d, *J* = 2.4 Hz, 1H), 6.64 (d, *J* = 8.0 Hz, 1H), 6.71 (t, ^2^*J*_HF_ = 76.0 Hz, 1H), 4.87–4.73 (m, 1H), 4.56–4.38 (m, 2H), 3.90 (d, *J* = 6.8 Hz, 2H), 3.32–3.07 (m, 2H), 1.33–1.26 (m, 1H), 0.71–0.58 (m, 2H), 0.42–0.29 (m, 2H). ^13^C NMR (100 MHz, DMSO-d_6_) δ 165.38, 162.72, 150.40, 149.95, 148.20, 144.27, 136.74, 127.87, 127.82, 124.02, 122.94, 122.01, 121.62, 120.89, 118.72, 115.23, 112.13, 110.54, 73.69, 66.58, 50.47, 26.68, 10.36, 3.56. HRMS (ESI) calcd for C_29_H_25_Cl_2_F_2_N_3_O_5_ [M+H]^+^ 603.1139, found 603.1137.

#### 3.1.31. (R)-2-(3-Ethoxy-4-methoxybenzamido)-3-(1H-indol-3-yl)propyl2,6-dichloroisonicotinate (**E2**)

Compound **E2** was prepared as a white solid at 84.5% yield from 3-ethoxy-4-methoxybenzoic acid in a similar manner to that described for compound **E1**. ^1^H NMR (400 MHz, Chloroform-d) δ 8.14 (s, 1H), 7.72 (d, *J* = 7.8 Hz, 1H), 7.66 (dd, *J* = 8.4, 2.0 Hz, 1H), 7.51 (d, *J* = 2.0 Hz, 1H), 7.42 (s, 2H), 7.39 (d, *J* = 2.4 Hz, 1H), 7.18 (d, *J* = 7.6 Hz, 1H), 7.13 (d, *J* = 2.4 Hz, 1H), 6.89 (d, *J* = 8.6 Hz, 1H), 6.79 (d, *J* = 8.0 Hz, 1H), 4.77 (m, 1H), 4.56–4.37 (m, 2H), 4.13 (q, *J* = 7.0 Hz, 2H), 3.92 (s, 3H), 3.27 (dd, *J* = 14.8, 5.2 Hz, 1H), 3.17–3.09 (m, 1H), 1.47 (t, *J* = 7.0 Hz, 3H). ^13^C NMR (100 MHz, DMSO-d_6_) δ 165.92, 162.57, 153.65, 150.36, 148.14, 148.03, 136.73, 127.84, 124.08, 123.79, 122.25, 122.14, 121.46, 118.83, 118.74, 113.30, 112.03, 111.77, 110.69, 66.12, 64.25, 56.20, 50.61, 26.75, 15.10. HRMS (ESI) calcd for C_27_H_25_Cl_2_N_3_O_5_ [M+H]^+^ 541.1171, found 541.1169.

#### 3.1.32. 5-Fluoro-2-[(phenyloxy)methyl]aniline (**31**)

To a solution of phenol (188 mg, 2.0 mmol) in MeCN, anhydrous K_2_CO_3_ (1.5 equiv) and KI (0.2 equiv) were added. After stirring for 20 min, 4-fluoro-2-nitrobenzyl bromide (2.0 mmol) was added. The mixture was heated to 85 °C and refluxed for 3 h. Purification through column chromatography (PE/EA 5:1) afforded intermediate 30.

To a solution of **30** in EtOH was added conc. HCl until pH 2–3, followed by iron powder (5.0 equiv). The mixture was heated to 50 °C and stirred for 1.5 h (reaction complete). Purification through column chromatography (PE/EA 3:1) afforded **31** (75.9% yield). ^1^H NMR (400 MHz, DMSO-d_6_) δ 7.29–7.23 (m, 2H), 7.15 (dd, *J* = 8.4, 6.8 Hz, 1H), 7.01–6.96 (m, 2H), 6.90 (d, *J* = 7.2 Hz, 1H), 6.43 (dd, *J* = 11.8, 2.6 Hz, 1H), 6.28 (td, *J* = 8.4, 2.6 Hz, 1H), 5.36 (s, 2H), 4.90 (s, 2H). ^13^C NMR (100 MHz, DMSO-d_6_) δ 164.75, 162.37, 158.89, 149.50, 131.66, 129.93, 121.16, 116.63, 115.41, 102.58, 102.37, 101.44, 101.20, 66.84.

#### 3.1.33. 3-(Cyclopropylmethoxy)-4-(difluoromethoxy)-N-(5-fluoro-2-(phenoxymethyl)phenyl)benzamide (**E3**)

To a solution of 4-(difluoromethoxy)-3-(cyclopropylmethoxy)benzoic acid (258 mg, 1.0 mmol) in DCM at 0 °C were added oxalyl chloride (1.5 equiv) and catalytic DMF (3–5 drops). After stirring for 30 min at 0 °C, the mixture was warmed to rt and stirred for 5 h. Volatiles were removed in vacuo to afford the crude acid chloride.

To a solution of intermediate **31** (1.0 mmol) in anhydrous DCM, triethylamine was added as an acid scavenger. A solution of the crude acid chloride in anhydrous DCM was added dropwise. After 30 min, the mixture was worked up with brine/DCM, dried (Na_2_SO_4_), and concentrated. Purification through column chromatography (PE/EA 4:1) afforded E3 as a white solid (398 mg, 86.8%). ^1^H NMR (400 MHz, Chloroform-d) δ 9.25 (s, 1H), 8.19 (dd, *J* = 11.2, 2.8 Hz, 1H), 7.50 (d, *J* = 2.0 Hz, 1H), 7.37–7.31 (m, 2H), 7.31–7.25 (m, 2H), 7.15–6.97 (m, 4H), 6.84 (td, *J* = 8.4, 2.6 Hz, 1H), 6.87 (t, ^2^*J*_HF_ = 76.0 Hz, 1H), 5.14 (s, 2H), 3.79 (d, *J* = 6.8 Hz, 2H), 1.28–1.20 (m, 1H), 0.67–0.57 (m, 2H), 0.35–0.26 (m, 2H). ^13^C NMR (100 MHz, Chloroform-d) δ 164.61, 164.18, 162.16, 157.48, 150.83, 143.35, 139.49, 139.38, 132.62, 130.85, 130.75, 130.05, 122.55, 122.31, 121.24, 121.20, 119.12, 118.45, 115.85, 115.06, 113.54, 113.26, 111.10, 110.88, 109.87, 109.60, 74.02, 69.52, 10.07, 3.33. HRMS (ESI) calcd for C_25_H_22_F_3_NO_4_ [M+H]^+^ 457.1501, found 457.1500.

#### 3.1.34. 1-(4-(3-(Cyclopropylmethoxy)-4-(difluoromethoxy)benzoyl)piperazin-1-yl)ethan-1-one (**E4**)

To a solution of 4-(difluoromethoxy)-3-(cyclopropylmethoxy)benzoic acid (258 mg, 1.0 mmol) in DCM at 0 °C were added oxalyl chloride (1.5 equiv) and catalytic DMF (3–5 drops). After stirring for 30 min at 0 °C, the mixture was warmed to rt and stirred for 5 h. Volatiles were removed in vacuo to afford the crude acid chloride.

To a solution of **32** (Boc-piperazine, 204 mg, 1.1 mmol) in anhydrous DCM, triethylamine was added as an acid scavenger. The crude acid chloride in anhydrous DCM was added dropwise. After 2 h (reaction complete), purification afforded **33**. Compound **33** was subjected to Boc deprotection with TFA to afford intermediate 34.

To a solution of **34** in DCM, triethylamine was added as an acid scavenger, followed by acetyl chloride (1.1 equiv). Upon completion, the mixture was worked up with brine/DCM, dried (Na_2_SO_4_), and concentrated. Purification through column chromatography (PE/EA 3:1) afforded E4 as a white solid powder (150 mg, 78.3%). ^1^H NMR (400 MHz, Methanol-d_4_) δ 7.20 (dd, *J* = 8.2, 1.6 Hz, 1H), 7.13 (t, *J* = 2.0 Hz, 1H), 7.00 (d, *J* = 7.2 Hz, 1H), 6.72 (t, ^2^*J*_HF_ = 75.2 Hz, 1H), 3.91 (dd, *J* = 8.0, 1.6 Hz, 2H), 3.52 (t, *J* = 51.8 Hz, 8H), 2.10 (s, 3H), 1.30–1.24 (m, 1H), 0.65–0.58 (m, 2H), 0.39–0.32 (m, 2H). ^13^C NMR (100 MHz, Methanol-d_4_) δ 170.65, 170.41, 150.73, 141.78, 133.44, 121.82, 119.39, 119.10, 116.60, 113.31, 113.16, 73.72, 19.85, 9.64, 2.22. HRMS (ESI) calcd for C_18_H_22_F_2_N_2_O_4_ [M+H]^+^ 368.1548, found 368.1545.

### 3.2. General Information on Biological Assays

Anti Rabbit IgG H+L was purchased from Proteintech (Wuhan, China); antibodies against p-NF-κB (#3033), NF-κB (#8242), p-CREB (#9198), CREB (#9197) and PKA-C (#5842) were purchased from Cell Signaling Technology (Danvers, MA, USA). The antibodies against α-Tubulin (GB11200) and GAPDH (GB15004) were purchased from Sewell Biotechnology Co. Ltd. (Wuhan, China); recombinant His-tagged PDE4B was procured from Bioesn Biotechnology (Beijing, China). The antibody against MUC5AC (ab3659) was procured from Abcam (Cambridge, UK). HBS-EP buffer and NTA sensor chips were obtained from Cytiva (Shanghai, China). PDE4B2 enzyme was sourced from BPS Bioscience Inc (Shanghai, China). The LANCE Ultra cAMP Detection Kit was purchased from PerkinElmer Inc (Waltham, MA, USA). TNF-α, IL-6 and cAMP ELISA kits were supplied by Fankewei Biotechnology Co., Ltd. (Shanghai, China).

#### 3.2.1. Cell Culture

RAW 264.7 cells were obtained from the Cell Bank of the Chinese Academy of Sciences and cultured in Dulbecco’s Modified Eagle Medium (DMEM) high-glucose medium supplemented with 10% fetal bovine serum (FBS) at 37 °C in a humidified incubator with 5% CO_2_. All experiments were conducted using cells between passages 3 and 6.

#### 3.2.2. MTT Assay

RAW 264.7 cells were seeded in 96-well plates at a density of 3 × 10^4^ cells/well and cultured for 12 h. Cells were then treated with various concentrations of **B7** (3.75, 7.5, 15, 30, 60, and 100 μM) for 24 h, followed by incubation with 20 μL of 5 mg/mL MTT solution for 4 h to form purple formazan crystals. The culture medium was replaced with 150 μL DMSO to dissolve the crystals. Absorbance was measured at 570 nm using a microplate reader after complete dissolution.

#### 3.2.3. Enzymatic Activity Assay

Blank, control and compound groups were assayed in 96-well plates. Each well received sequential additions: 83.3 μL Tris buffer, 16.2 μL of 0.5 mM cAMP, 12 μL calf intestinal alkaline phosphatase (CIAP), and 13.5 μL of varying concentrations of compounds (prepared in Tris buffer). CIAP hydrolyzes the 5′-phosphate group of cAMP. Blank and control groups received equivalent volumes of Tris buffer. Finally, 10 μL PDE4B protein was added to all groups except the blank group, which received an equal volume of Tris buffer. After incubation at 37 °C for 20–60 min, reactions were terminated by adding 25 μL 40% HClO_4_, followed by 30 μL 280 mM ammonium molybdate and 30 μL 1 mM malachite green. Absorbance was measured at 630 nm after 15 min incubation at 37 °C. Inhibition rates were calculated as follows:

$$\mathrm{Inhibition}\;\mathrm{rate}\;(\%)\;=\;\frac{\left(A_{1}-A_{0}\right)-\left(A_{2}-A_{0}\right)}{\left(A_{1}-A_{0}\right)}\times 100\%$$

where *A*_0_, *A*_1_, and *A*_2_ represent absorbance values of the blank, control, and compound groups, respectively.

#### 3.2.4. SPR

The sensor chip surface was conditioned with a 350 mM EDTA injection pulse, followed by flowing 0.5 mM NiCl_2_ in running buffer. His-tagged PDE4B was immobilized onto NTA sensor chips. Serial concentrations of **B7** (0, 3.125, 6.25, 12.5, 25, 50, 100 μM) prepared in HBS-EP buffer were injected over the captured protein surface to facilitate ligand binding. Sensorgram data were analyzed and fitted using Biacore T200 Evaluation Software v3.2 to determine the equilibrium dissociation constant (KD).

#### 3.2.5. Molecular Docking

Molecular docking was performed using LeDock software (version 1.0), selected for its balance of docking accuracy and computational efficiency. The PDE4B crystal structure (PDB ID: 1XMU) was retrieved from the RCSB Protein Data Bank and preprocessed with LePro, including hydrogen atom addition, charge assignment, and binding pocket definition. Flexible ligand conformational sampling was conducted using default parameters, with the lowest binding energy conformation selected for the visualization of key intermolecular interactions in PyMOL (version 2.5.2).

#### 3.2.6. Molecular Dynamics

Molecular dynamics (MD) simulations were performed using the Dynamics module in Flare Suite 2022. The protein–ligand complex was solvated in explicit TIP4Pew water molecules within an orthorhombic periodic boundary box (10 Å buffer). After removing overlapping waters and adjusting ionic strength to 0.150 M, the system underwent: (1) 100 ps Brownian dynamics equilibration, (2) 5 ns NVT ensemble equilibration at 300 K, (3) 5 ns NPT ensemble equilibration at 300 K/1.0 bar, and (4) 100 ns production MD. Energy minimization employed steepest descent followed by conjugate gradient algorithms (max 2000 iterations). Simulations used a 4 fs timestep with energy/structure sampling every 20 ps.

#### 3.2.7. Measurement of NO, TNF-α, and IL-6

Cells in 96-well plates were divided into control, LPS, and LPS plus various concentrations of **B7** (3.75, 7.5, 15, 30, 60 μM) groups. RAW 264.7 cells were seeded at 3 × 10^4^ cells/well for 12 h, then treated with 1 μg/mL LPS and different concentrations of **B7** for 24 h. Subsequently, 50 μL of cell supernatant was mixed sequentially with 50 μL of Griess reagent I and II, and absorbance was measured at 540 nm. Levels of TNF-α and IL-6 in the cell culture supernatant were quantified using a double-antibody sandwich ELISA according to the manufacturer’s instructions.

#### 3.2.8. Animal Experiments

Male Sprague Dawley (SD) rats (7–8 weeks old) were purchased from Zhejiang Vital River Laboratory Animal Technology Co., Ltd. (License No.: SCXK (Zhe) 2024-0001, Jiaxing, China). Prior to experiments, animals were acclimatized for 7 days under specific pathogen-free (SPF) conditions with a 12/12 h light/dark cycle. All animal experimental procedures were reviewed and approved by the Institutional Animal Care and Use Committee of Jiangxi Zhonghong Boyuan Biotechnology Co., Ltd. (Approval No.: LL-202404180004, Nanchang, China).

#### 3.2.9. Rat Model for Toxicity

For acute toxicity assessment, 40 Sprague-Dawley rats (6–9 weeks old) were acclimatized for 7 days and randomly assigned to four groups (n = 10, 5 males/5 females) by body weight: control, **B7** low-dose (8 mg/kg), medium-dose (16 mg/kg), and high-dose (32 mg/kg). **B7** was administered via tail vein injection at a rate of 10 min/animal, while controls received equivalent saline volumes. Animals were monitored daily for 14 days post-administration.

For chronic toxicity assessment, 80 Sprague Dawley rats (6–9 weeks old) were acclimatized for 7 days and randomized into four groups (n = 20, 10 males/10 females) by body weight: control, **B7** low-dose (1.2 mg/kg), medium-dose (2.5 mg/kg), and high-dose (7.5 mg/kg). Daily tail vein injections of **B7** were administered at 2 mL/kg/min for 4 weeks, with controls receiving equivalent saline volumes. Animals were euthanized after 4 weeks for toxicological evaluation.

#### 3.2.10. Pharmacokinetics and Tissue Distribution Study in Wistar Rats

The pharmacokinetics of **B7** in Wistar rats were evaluated following tail vein injection. Animals received a single dose of **B7** at 0.2 mg/kg (n = 12, half male and half female), 1 mg/kg (n = 12, half male and half female), or 5 mg/kg (n = 8, half male and half female). Blood samples (0.3 mL) were collected at 0, 0.25, 0.5, 1, 2, 4, 8, and 24 h post-dose. Plasma concentrations of **B7** were determined by LC-MS/MS, and pharmacokinetic parameters are presented as mean ± SD.

The tissue distribution of **B7** was evaluated in Wistar rats following intravenous administration. The animal study was approved by the Qingdao Marine Biomedical Research Institute (Approval No.: SYXK20210009, Qingdao, China). At 0.5, 1, and 3 h post-dose, tissues (including muscle, fat, spleen, stomach, heart, liver, lung, esophagus, large intestine, small intestine, and brain) were immediately harvested. Blood samples were centrifuged at 4000 rpm for 10 min at 4 °C to obtain plasma. Tissue samples were weighed, homogenized in saline, and processed. The concentrations of **B7** in plasma and tissue homogenates were determined using a validated LC-MS/MS method.

#### 3.2.11. Establishment of a Rat Model of COPD Induced by Cigarette Smoke and LPS

A total of 98 male Sprague Dawley (SD) rats (SPF grade) were randomly assigned to seven groups (n = 14 per group): control, model, Roflumilast (20 mg/kg), and **B7** at three dose levels (0.1, 0.3, and 1 mg/kg). To induce chronic airway inflammation, rats in all but the control group were subjected to a 66-day modeling protocol. This involved the intratracheal instillation of lipopolysaccharide (LPS; 200 μg/rat) on days 1 and 14, combined with daily exposure to cigarette smoke (CS) from day 2 to 66 (excluding LPS challenge days).

Starting on day 50, a 14-day treatment was initiated. Animals in the **B7** group were administered the target compound via intravenous (i.v.) injection, while the Roflumilast group received Roflumilast through oral gavage (p.o.). The control and model groups were treated with the vehicle (0.5% DMSO) in an identical manner. Twenty-four hours after the final administration, rats were anesthetized with sodium pentobarbital, and tissues were harvested for further analysis.

#### 3.2.12. Invasive Assessment of Pulmonary Function in Rats

Twenty-four hours after the final administration, pulmonary function was measured in eight randomly selected rats from each group. Following anesthesia with (1% pentobarbital sodium, 10 mL/kg, i.p), a tracheostomy was performed, and a cannula was inserted into the trachea. The rats were connected to a small animal ventilator (AniRes2005 system; Beilanbo, Beijing, China) and ventilated. After a stabilization period, the baseline values for key respiratory parameters, including forced vital capacity (FVC) and forced expiratory volume in 100 milliseconds and 300 milliseconds, were continuously recorded for 3 min. The average values were used for subsequent statistical analysis.

#### 3.2.13. ELISA Analysis of Serum and Bronchoalveolar Lavage Fluid (BALF)

Following treatment, the remaining six rats per group were anesthetized. After thoracotomy, the trachea and lungs were exposed. Whole blood was collected via cardiac puncture and centrifuged at 3500× *g* for 10 min at 4 °C. The serum was aliquoted and stored at −20 °C. The right main bronchus was ligated, and the left lung was lavaged three times through a cannula inserted at the carina with 2 mL of sterile saline per wash (approximately 1.5 mL recovered per lavage). The pooled BALF was centrifuged at 1000× *g* for 10 min at 4 °C, and the supernatant was stored at −20 °C. Levels of TNF-α and IL-6 in both serum and BALF supernatant were quantified using ELISA kits.

#### 3.2.14. Quantification of Inflammatory Cells in Bronchoalveolar Lavage Fluid (BALF)

Following BALF collection, the cell pellet was washed three times with PBS and resuspended in 50 μL of PBS. Total cell counts were determined using a hemocytometer. For differential cell counting, 30 μL of the cell suspension was cytocentrifuged onto slides, stained with Wright–Giemsa stain, and examined under an optical microscope to quantify inflammatory cell populations.

#### 3.2.15. Histopathological Evaluation

Rat organ tissues were fixed in 4% paraformaldehyde, dehydrated, embedded in paraffin, and sectioned for hematoxylin and eosin (H&E) staining and Alcian blue–periodic acid–Schiff (AB-PAS) staining. Histopathological alterations and mucus content were examined under an optical microscope. The mean linear intercept (MLI) and mean alveolar number (MAN) were quantified using Image-Pro Plus 6.0 image analysis software. The percentage of AB-PAS-positive cells was statistically analyzed to evaluate airway goblet cells and mucus secretion. The percentage of positive goblet cells was calculated as follows: (number of AB-PAS-positive goblet cells/total number of columnar epithelial cells) × 100%.

#### 3.2.16. Immunohistochemical Staining

MUC5AC mucin expression in lung tissues was evaluated through immunohistochemical (IHC) staining. Tissue sections underwent microwave-mediated antigen retrieval (80–90 °C, 600–700 W, 10 min) for enzyme inactivation, followed by blocking and sequential incubation with primary and secondary antibodies. Stained sections were counterstained, dehydrated, cleared, and mounted for scanning using a digital slide scanner.

For quantitative analysis, IHC-stained sections were imaged with an Olympus BX-UCB image acquisition system. The integrated optical density (IOD) of immunohistochemical staining was semi-quantitatively analyzed using Image-Pro Plus 7.0 software.

#### 3.2.17. Western Blot

As previously described [56], cellular samples were lysed in RIPA buffer. Lysates were centrifuged at 12,000 rpm for 10 min, and supernatants were denatured at 100 °C for 10 min. Proteins were separated on 10% SDS-PAGE gels with 5% stacking gels, then transferred to nitrocellulose membranes. Membranes were blocked with 5% bovine serum albumin (BSA) for 2 h, incubated with primary antibodies at 4 °C overnight, and washed thrice with TBST (5 min/wash). Secondary antibody incubation proceeded at room temperature for 60 min, followed by three TBST washes. Protein bands were visualized using alkaline phosphatase chromogenic substrate. Band intensities were quantified with ImageJ v1.53a (NIH, Bethesda, MD, USA).

#### 3.2.18. Immunofluorescence Analysis

RAW 264.7 cells were seeded at a density of 1 × 10^5^ cells/well and cultured for 12 h. Cells were treated with 1 μg/mL lipopolysaccharide (LPS) and 30 μM **B7**, then fixed with 4% paraformaldehyde for 20 min at 4 °C. After blocking with 5% bovine serum BSA for 1 h, cells were incubated overnight with an anti-NF-κB antibody. Subsequently, cells were incubated with a fluorescein isothiocyanate (FITC)-conjugated secondary antibody for 1 h at 37 °C in the dark, followed by counterstaining with DAPI for 15 min at 37 °C in darkness. Fluorescence was visualized using a fluorescence microscope (Nikon, Tokyo, Japan). For each sample, three random fields were imaged under a confocal laser scanning microscope. Images were analyzed using ImageJ software (v1.53a; NIH, Bethesda, MD, USA).

#### 3.2.19. Measurement of cAMP Levels

Cells were seeded at a density of 8 × 10^5^ cells/well and cultured for 12 h, followed by treatment with **B7** at concentrations of 3.75, 7.5, 15, and 30 μM. After treatment, cells were collected, lysed, and centrifuged. The cAMP content was quantified using ELISA.

#### 3.2.20. Statistical Analysis

All data are presented as mean ± SEM. Statistical analyses were performed using GraphPad Prism 8.0.2 software, employing one-way ANOVA followed by Tukey’s multiple comparisons. Differences were considered statistically significant at *p* < 0.05.

## 4. Conclusions

In summary, this study designed and synthesized 32 novel PDE4 inhibitors based on marine-derived natural products and PDE4 structural insights. The structure–activity relationship (SAR) analysis revealed that guanidine-containing compounds with cyclopropoxy and difluoromethoxy substituents on the phenyl ring exhibited potent inhibition. Linker length significantly impacted activity: cyclic linkers showed a weaker PDE4B inhibition than linear analogs, likely due to steric incompatibility with the PDE4B binding pocket. For non-guanidine compounds, terminal carboxyl groups enhanced activity, while bulky hydrophobic substituents in the S-region subpocket reduced inhibition. Compound **B7** was selected as the lead candidate through in vitro screening.

Molecular dynamics simulations and SPR experiments revealed key binding residues between **B7** and PDE4B, providing a theoretical basis for future inhibitor design. The binding affinity (KD) was determined to be 2.151 μM. In an LPS-induced RAW264.7 cell inflammation model, **B7** significantly reduced intracellular levels of TNF-α and IL-6. In a rat COPD model induced by LPS combined with smoke exposure, high-dose **B7** markedly reduced inflammatory cell infiltration and cytokine production, alleviated pulmonary inflammation, decreased expiratory resistance, and inhibited airway mucus secretion, thereby improving lung function. In vitro mechanistic studies revealed that the marine-inspired derivative **B7** exerts anti-inflammatory effects through the dual modulation of the cAMP-PKA-CREB and NF-κB pathways. Tissue distribution studies showed predominant drug accumulation in the lungs with minimal brain penetration, suggesting a low risk of central nervous system side effects and indicating the potential for pulmonary disease treatment. Coupled with its excellent safety profile observed in rat toxicity evaluations, **B7** emerges as a potent and promising strategy for the treatment of COPD.

## Figures and Tables

**Table 1:** Inhibition of target compounds on PDE4B.

Compounds	PDE4B Inhibition Rate (10 μM) ^1^
Roflumilast	89.7 ± 0.84%
MBZ	45.41 ± 0.95%
**A1**	81.50 ± 1.33%
**A2**	52.66 ± 1.11%
**A3**	34.69 ± 1.03%
**A4**	35.38 ± 1.88%
**A5**	73.60 ± 1.88%
**A6**	54.31 ± 0.99%
**A7**	39.29 ± 2.06%
**A8**	40.40 ± 0.88%
**A9**	13.49 ± 2.81%
**B1**	27.10 ± 1.16%
**B2**	15.96 ± 2.16%
**B3**	38.29 ± 1.25%
**B4**	15.87 ± 1.68%
**B5**	24.58 ± 1.98%
**B6**	15.34 ± 2.01%
**B7**	93.49 ± 3.11%
**C1**	4.76 ± 3.81%
**C2**	1.08 ± 1.85%
**C3**	2.13 ± 0.61%
**C4**	30.99 ± 2.03%
**D1**	56.57 ± 0.68%
**D2**	78.36 ± 2.31%
**D3**	84.59 ± 0.88%
**D4**	9.46 ± 1.55%
**D5**	71.83 ± 2.81%
**D6**	95.64 ± 0.51%
**D7**	95.17 ± 0.63%
**D8**	37.46 ± 0.91%
**E1**	0%
**E2**	0%
**E3**	73.23 ± 0.56%
**E4**	18.92 ± 1.66%

^1^ Data are expressed as mean ± SD from three independent experiments.

**Table 2:** The IC_50_ values of selected compounds against PDE4B.

Compounds	IC_50_ (μM) ^1^
MBZ	91.93 ± 0.27
**A1**	3.93 ± 0.26
**B7**	0.9 ± 0.35
**D6**	2.59 ± 0.25
**D7**	2.35 ± 0.09
**E3**	5.09 ± 0.38

^1^ IC_50_: 50% inhibitory concentration and the IC_50_ value expressed as the mean ± SD for three determinations.

**Table 3:** Pharmacokinetic data of **B7** at various doses (i.v.).

Parameter	0.2 mg/kg	1 mg/kg	5 mg/kg
AUC_0-t_	17.38 ± 7.61	101.86 ± 63.74	570.78 ± 188.56
AUC_0-∞_	18.85	124.48 ± 53.51	594.96 ± 195.65
t_1/2_	/	0.43 ± 0.48	0.93 ± 0.60
Vz	/	4.65 ± 4.22	11.18 ± 4.72
CLz	10.61	9.05 ± 2.79	9.56 ± 4.53
MRT_0-t_	0.28	0.22 ± 0.28	0.58 ± 0.43
Cmax	160.26 ± 102.83	533.44 ± 344.69	2629.63 ± 1220.89

## Data Availability

The data presented in this study are available on request from the corresponding author.

## Supplementary Materials

The following supporting information can be downloaded at: https://www.mdpi.com/article/10.3390/md24030090/s1, Figure S1. Molecular docking and molecular dynamics of **B7** with PDE4B; Table S1. Molecular docking scores of **B7** with PDE family proteins; Figure S2: Kinetic binding diagram of **B7** and PDE4B; Figure S3. Acute toxicity study and long term toxicity of compound **B7** in rats; Figure S4. Long term toxicity of compound **B7** in rats; Figure S5. Effect of **B7** on serum biochemical parameters in male and female rats; Figure S6. Tissue distribution profile of **B7** in rats at different time points post-administration; The details of ^1^H NMR and ^13^C NMR for all compounds.

## Abbreviations

The following abbreviations are used in this manuscript:

AB-PAS	Alcian blue–periodic acid–Schiff
BALF	Bronchoalveolar lavage fluid
cAMP	Cyclic adenosine monophosphate
CNS	Central nervous system
COPD	Chronic obstructive pulmonary disease
CREB	cAMP-response element binding protein
CS	Cigarette smoke
Rof	Roflumilast
FEV1	Forced expiratory volume in 1 s
FITC	Fluorescein 5-isothiocyanate
FVC	Forced vital capacity
IL-6	Interleukin-6
LPS	Lipopolysaccharides
MAN	Mean alveolar number
MLI	Mean linear intercept
NMR	Nuclear magnetic resonance
NO	Nitric oxide
PDE4	Phosphodiesterase-4
PKA	Protein kinase A
ROS	Reactive oxygen species
RMSD	Root Mean Square Deviation
SAR	Structure-activity relationship
SPR	Surface plasmon resonance
TLC	Thin-layer chromatography
TNF-α	Tumor necrosis factor-α
