# Supplementary material for: Discovery of Marine-Inspired Guanidine-Based PDE4 Inhibitors for the Treatment of Chronic Obstructive Pulmonary Disease
Source: Mar Drugs. 2026 Feb 25;24(3):90. doi: 10.3390/md24030090 (PMC13028064; doi:10.3390/md24030090)
Supplement: Supplementary file 1 [file marinedrugs-24-00090-s001.zip › marinedrugs-4155499-supplementary.pdf]

Supporting Information

Discovery of Marine-Inspired Guanidine-Based PDE4 Inhibitors for the Treatment of Chronic Obstructive Pulmonary Disease

Xinglong Dai[1, †], Jie Hao[1, †], Yan Zhang [1, †], Yaping Yang[1], Wanli Meng[1], Fang Lu[1], Jianchun Zhao[2], Guanhua Du [2], Shengbiao Wan [1], Jiejie Hao [1,2 *]

[1] *Laboratory for Marine Drugs and Bioproducts, Qingdao Marine Science and Technology Center, School of Medicine and Pharmacy, Ocean University of China, Qingdao, 266071, China*

[2] *Marine Biomedical Research Institute of Qingdao, Qingdao 266071, China*

[†]These authors made equal contributions to this work.

[*]Corresponding authors

Email address: 2009haojie@ouc.edu.cn (Jiejie Hao)

Contents:

- **Molecular Docking and Molecular Dynamics of B7 with PDEs**
- **Toxicity Study**
- ***In Vivo* Tissue Distribution**
- **Spectra of Target Compounds**

1 Molecular Docking and Molecular Dynamics of B7 with PDEs

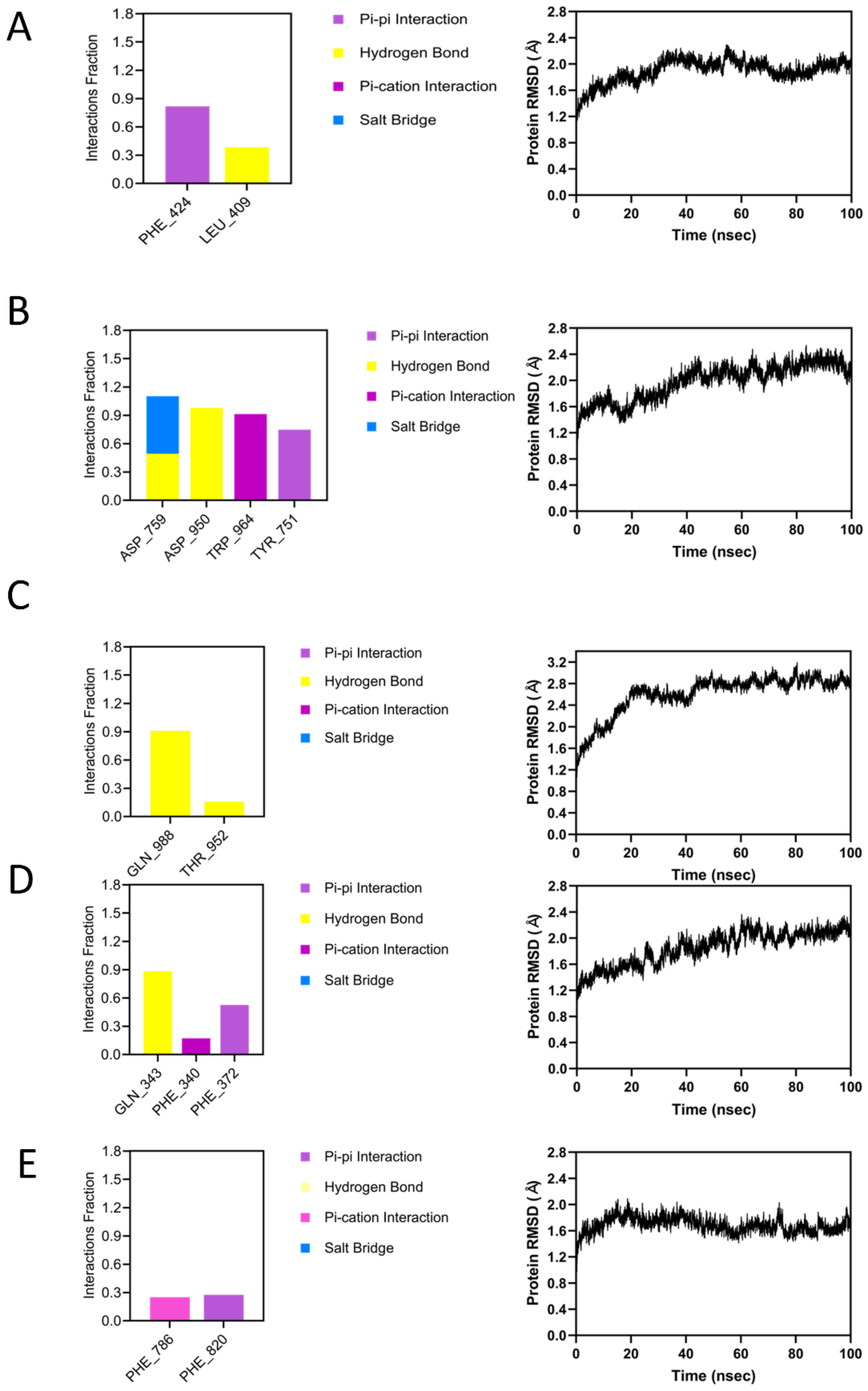

Figure S1 Molecular Docking and Molecular Dynamics of B7 with PDEs (A) Interaction frequencies of amino acid residues and root-mean-square deviation (RMSD) profiles from molecular dynamics (MD) simulations for B7 within the binding pocket

of PDE1B (PDB ID: 5UOY). (B) Interaction frequencies of amino acid residues and RMSD profiles from MD simulations for B7 within the binding pocket of PDE3A (PDB ID: 5UOY). (C) Interaction frequencies of amino acid residues and RMSD profiles from MD simulations for B7 within the binding pocket of PDE3B (PDB ID: 1SO2). (D) Interaction frequencies of amino acid residues and RMSD profiles from MD simulations for B7 within the binding pocket of PDE4D (PDB ID: 7W4Y). (E) Interaction frequencies of amino acid residues and RMSD profiles from MD simulations for B7 within the binding pocket of PDE5A (PDB ID: 1XP0).

Table S1 Molecular docking scores of B7 with PDE family proteins

**Target (PDB** ID**)**	**RankScore**	**dG**	**VS Score**
PDE4B(1XMU)	-7.668	-9.803	-10.909
PDE1B(5UOY)	-3.052	-9.551	-8.615
PDE3A(7L28)	-5.847	-9.124	-9.506
PDE3B(1SO2)	-4.079	-8.437	-8.602
PDE4D(7W4Y)	-4.832	-9.28	-9.637
PDE5A(1XP0)	-4.371	-8.418	-8.835

2. The Binding of the PDE4B Protein with B7

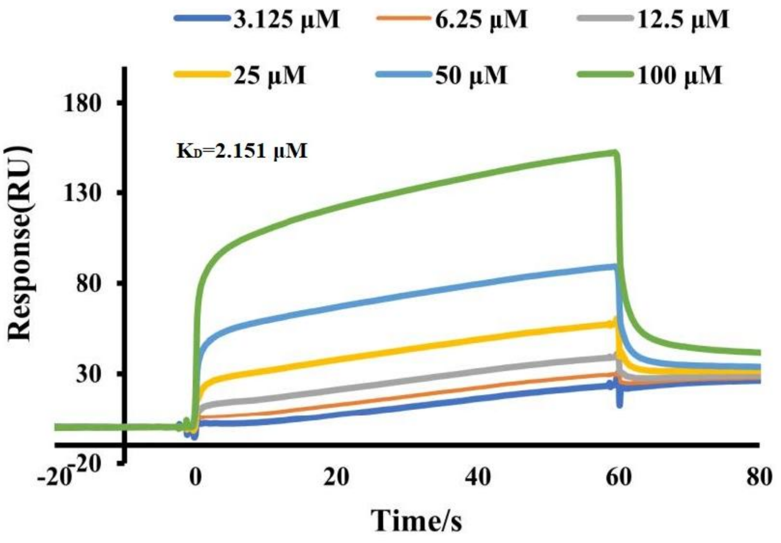

Figure S2. Kinetic binding diagram of B7 and PDE4B.

3.Toxicity Study

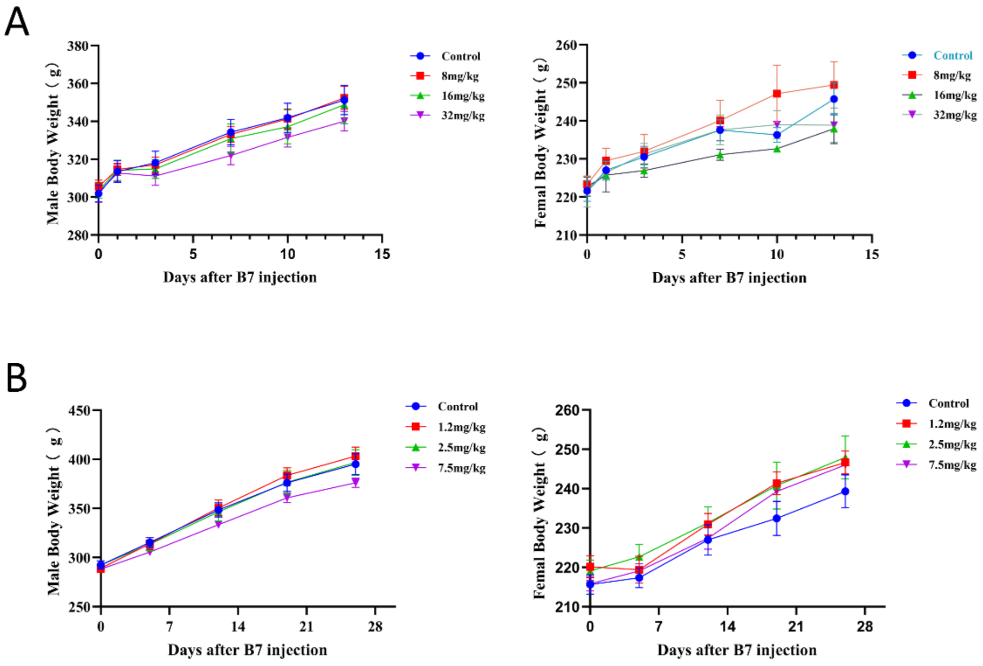

Figure S3. Acute toxicity study and long term toxicity of compound B7 in rats. (A) The body weight of rats in the acute toxicity group (n=40). (B) The body weight of rats in the long term toxicity group (n=80).

3.Toxicity Study

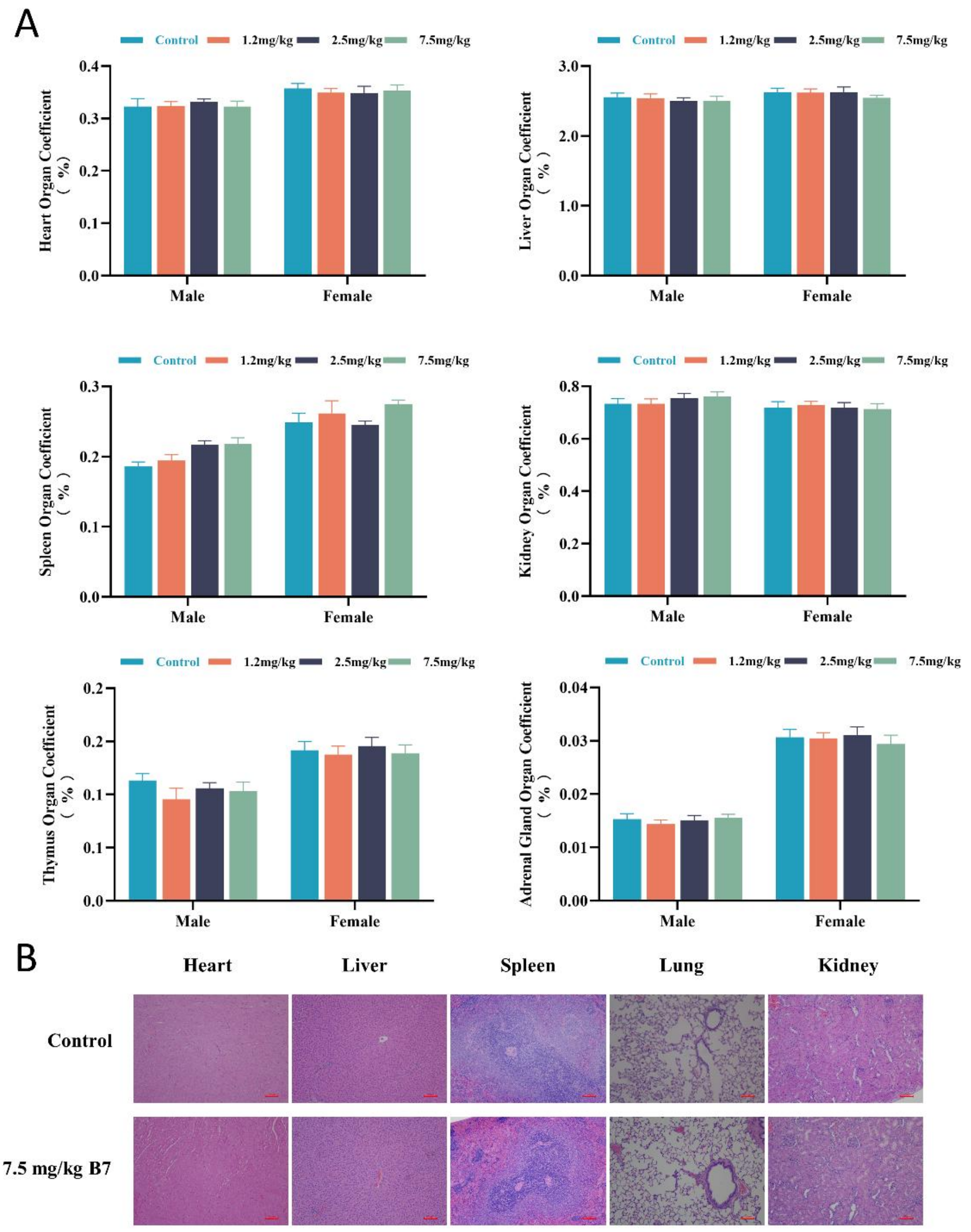

Figure S4. Long term toxicity of compound B7 in rats. (A) Effects of chronic B7 administration on organ-to-body weight ratios in rats. Data represent the means ± SEM (n=10). (B) HE staining for pathological changes of rat tissues (Scale bar = 100 μm).

3. Toxicity Study

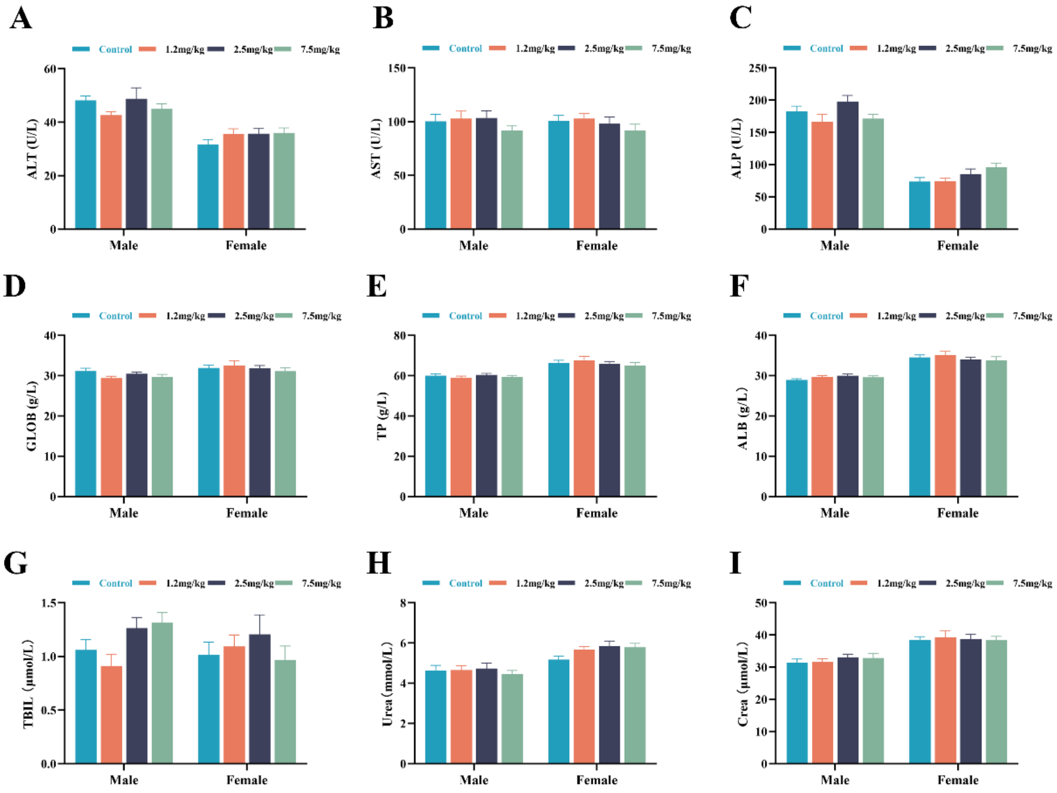

Figure S5. Effect of **B7** on serum biochemical parameters in male and female rats. (A) Alanine aminotransferase (ALT); (B) Aspartate aminotransferase (AST); (C) Alkaline phosphatase (ALP); (D) Globulin (GLOB); (E) Total protein (TP); (F) Albumin (ALB); (G) Total bilirubin (TBIL); (H) Urea; (I) Creatinine (Crea).

4. *In Vivo* Tissue Distribution

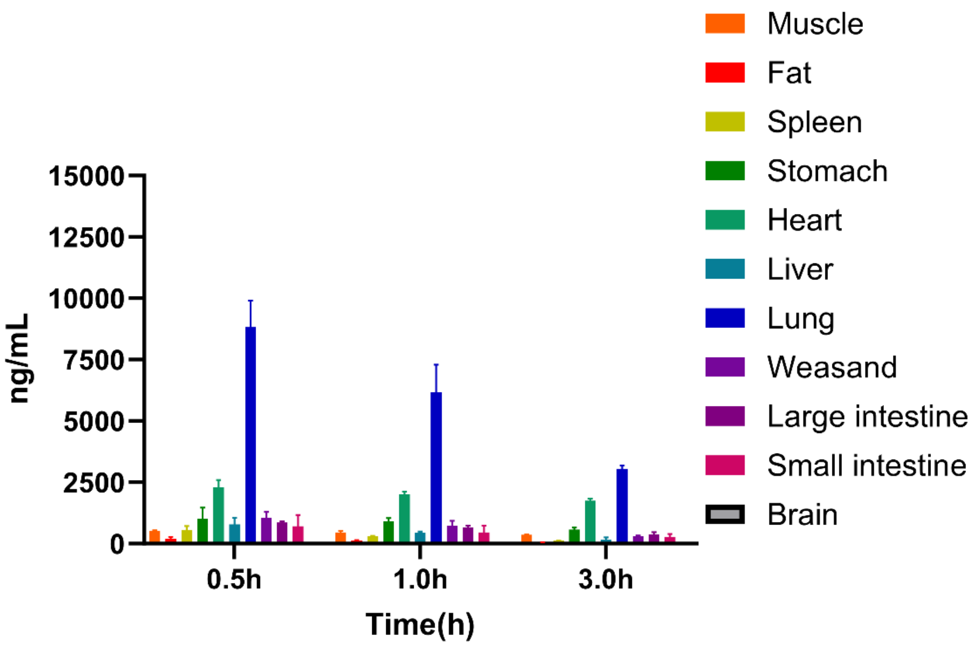

Figure S6. Tissue distribution profile of B7 in rats at different time points post-administration. The histogram illustrates the concentration of B7 (ng/mL) in various tissues at 0.5, 1.0, and 3.0 hours after administration. Data are presented as mean ± SEM. Key: color-coded bars represent different tissues (orange: muscle, red: adipose, etc.).

Spectra of Target Compounds

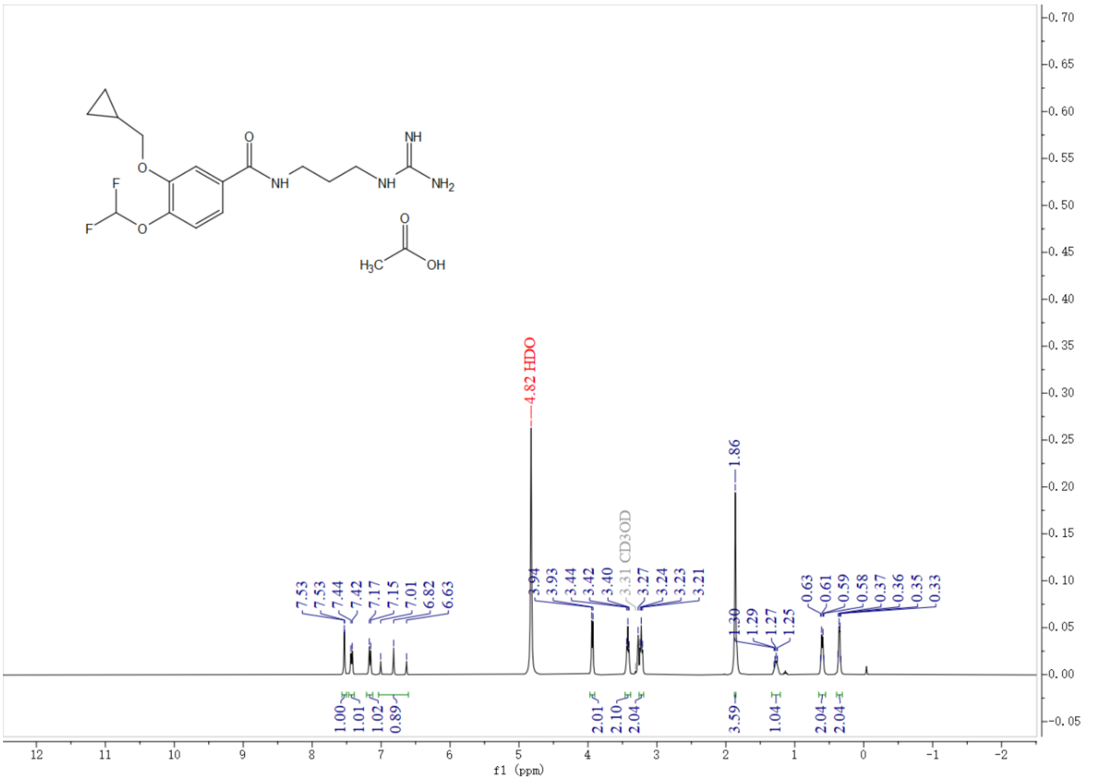

^{1}H NMR of compound A1(400MHz CD_3OD)

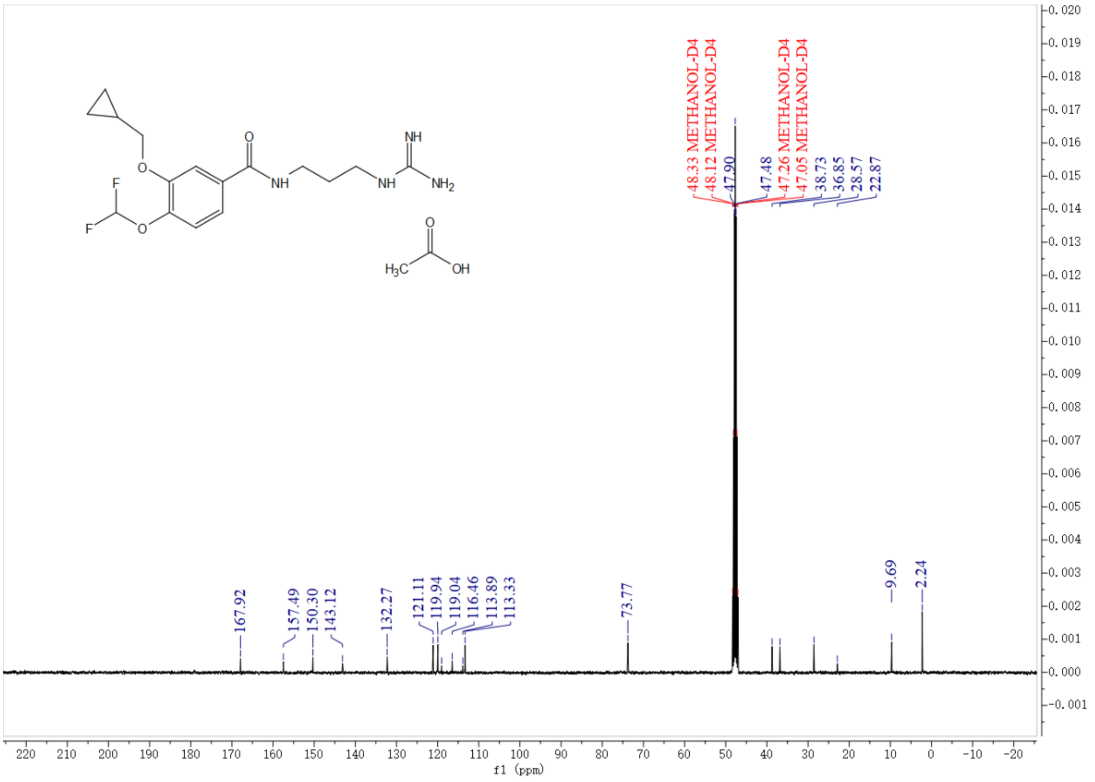

^{13}C NMR of compound A1(400MHz CD_3OD)

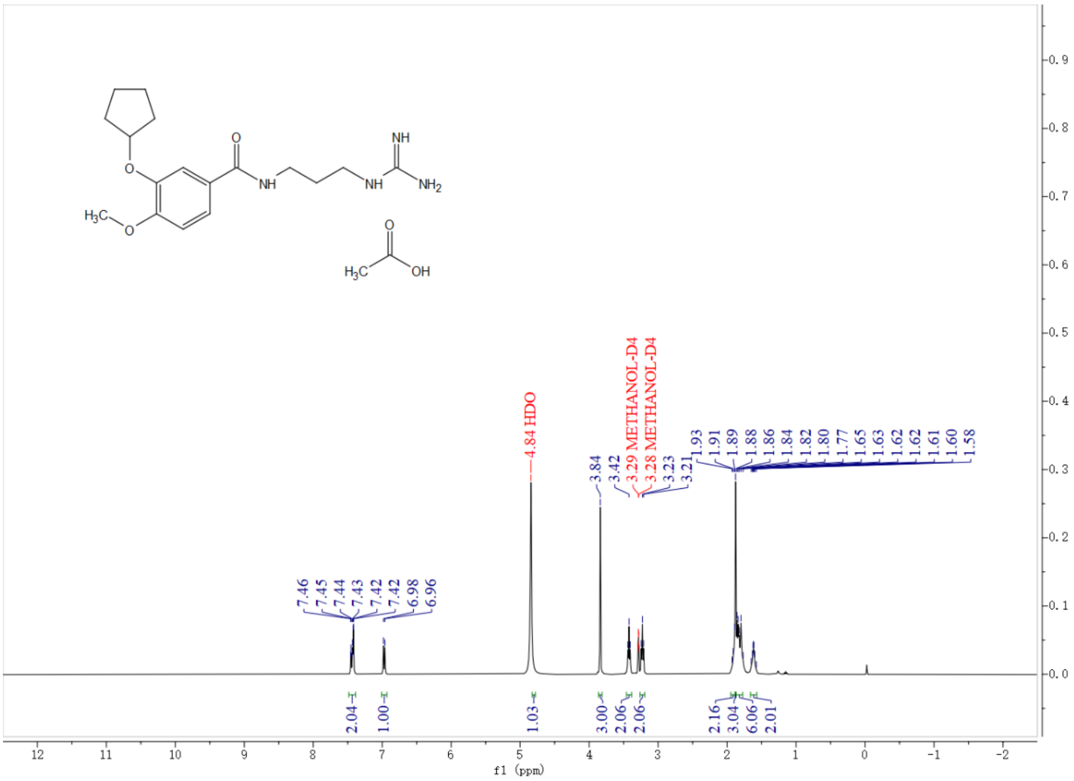

^{1}H NMR of compound A2(400MHz CD_3OD)

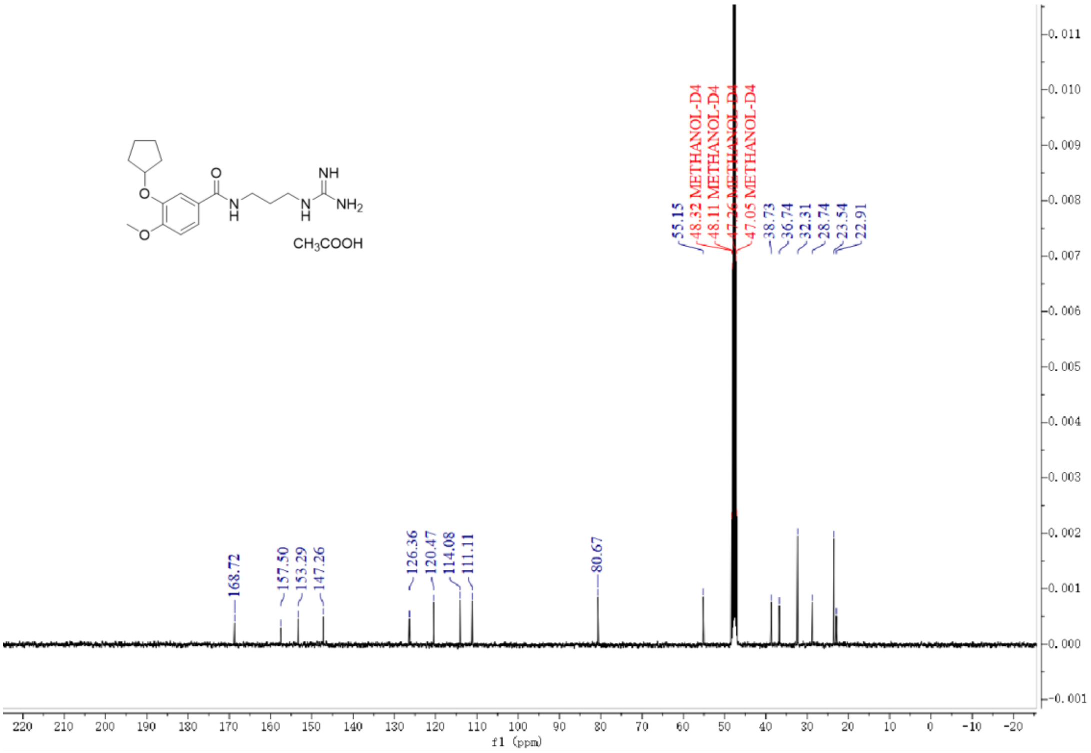

^{13}C NMR of compound A2(400MHz CD_3OD)

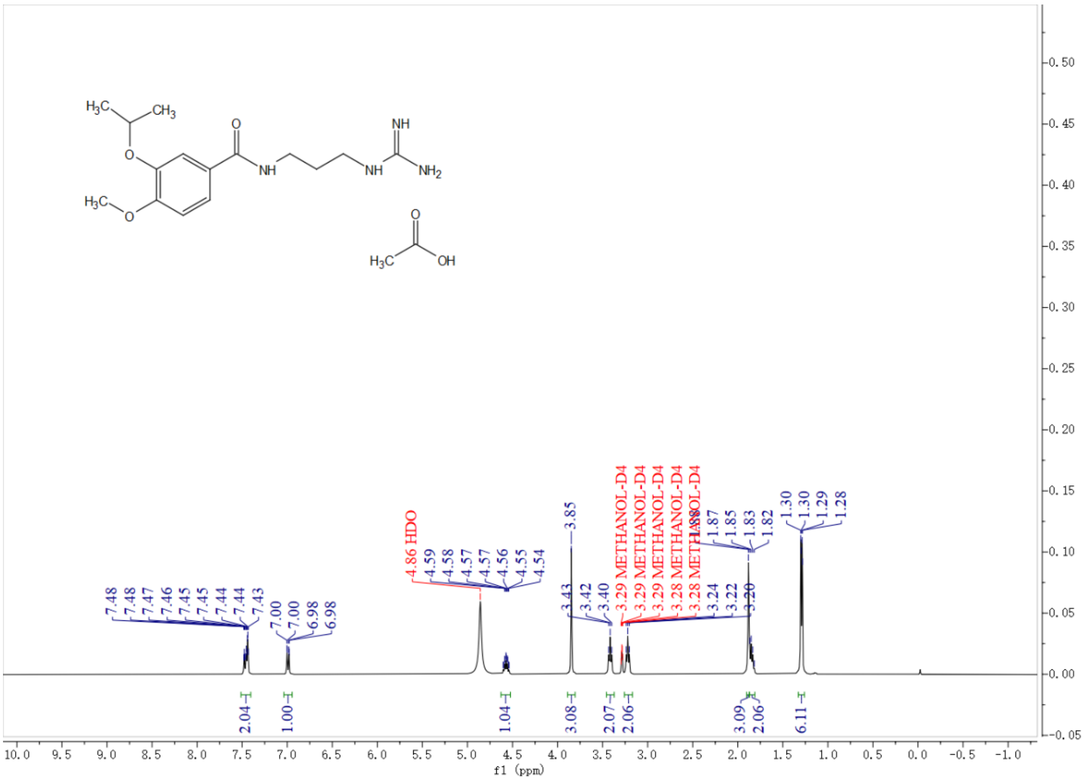

^{1}H NMR of compound A3(400MHz CD_3OD)

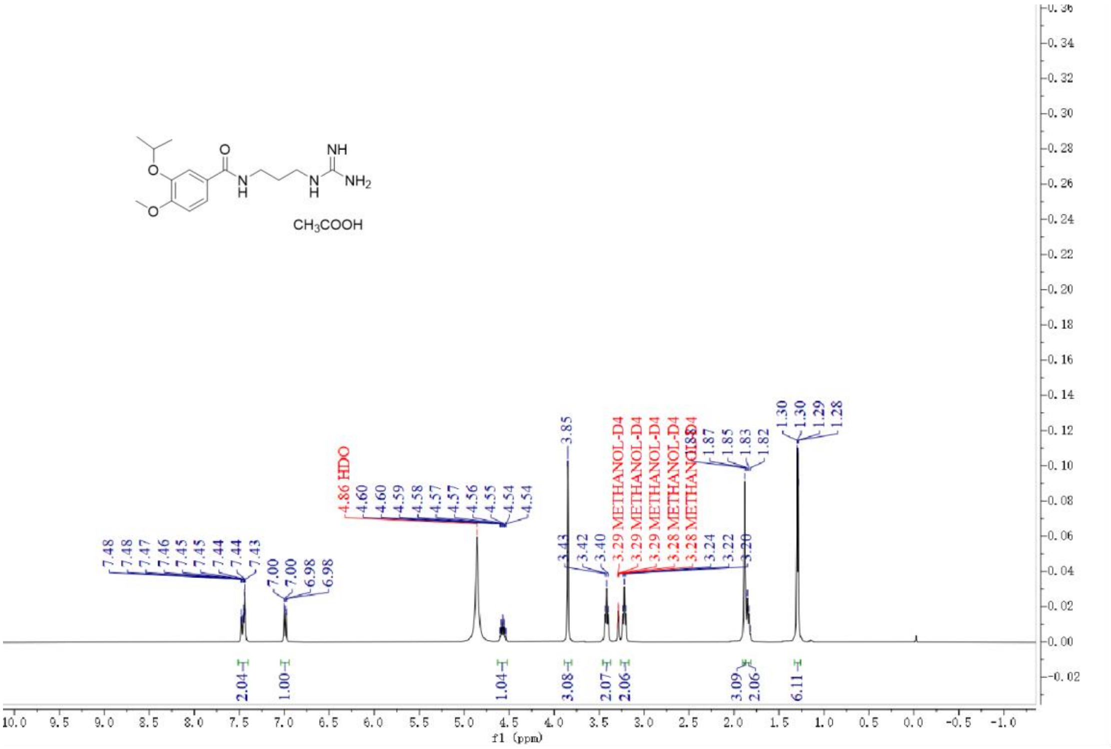

^{13}C NMR of compound A3(400MHz CD_3OD)

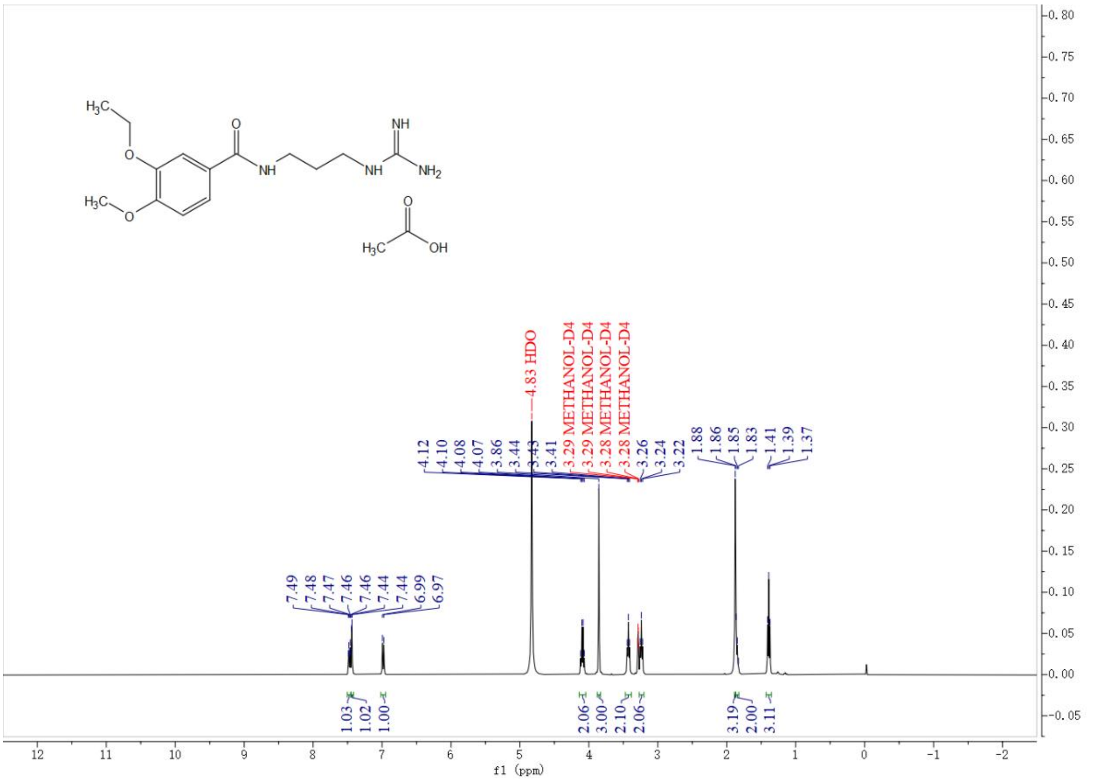

^1H NMR of compound A4(400MHz CD_3OD)

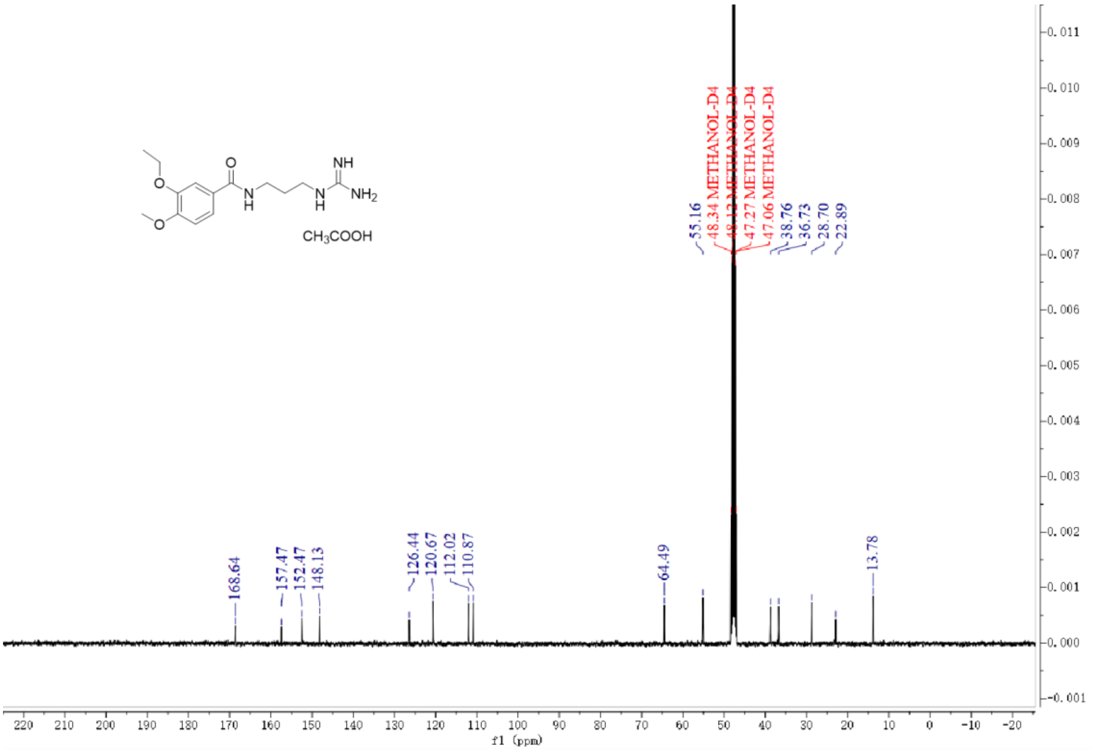

^{13}C NMR of compound A4(400MHz CD_3OD)

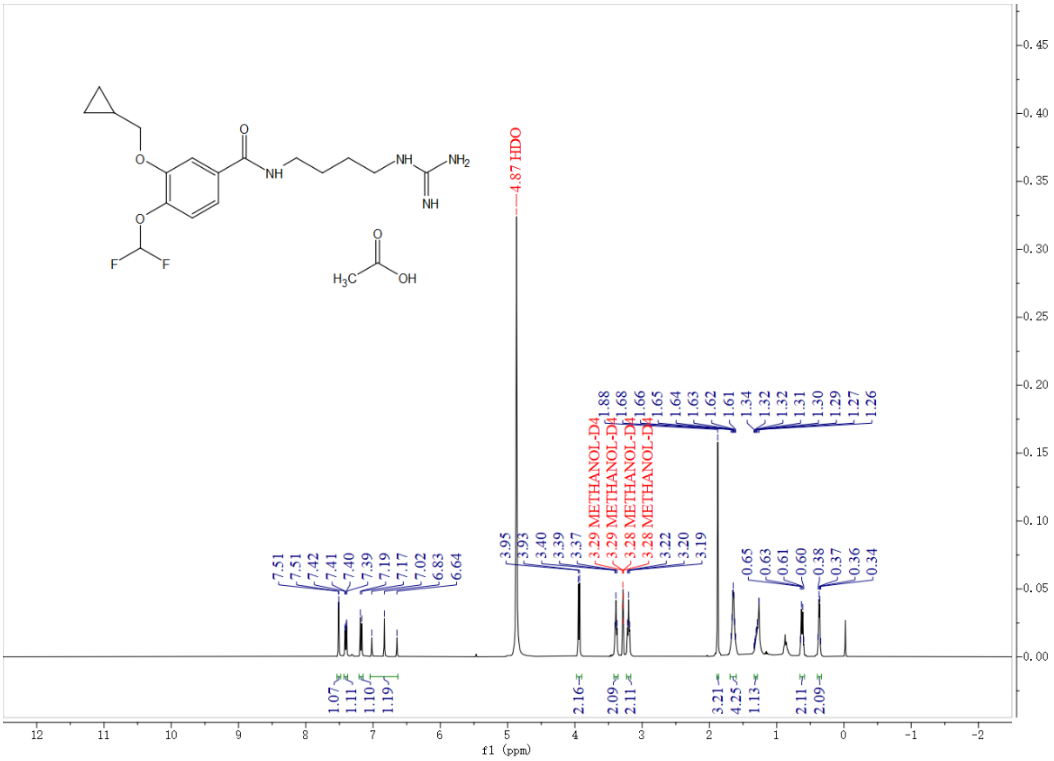

^{1}H NMR of compound A5(400MHz CD_3OD)

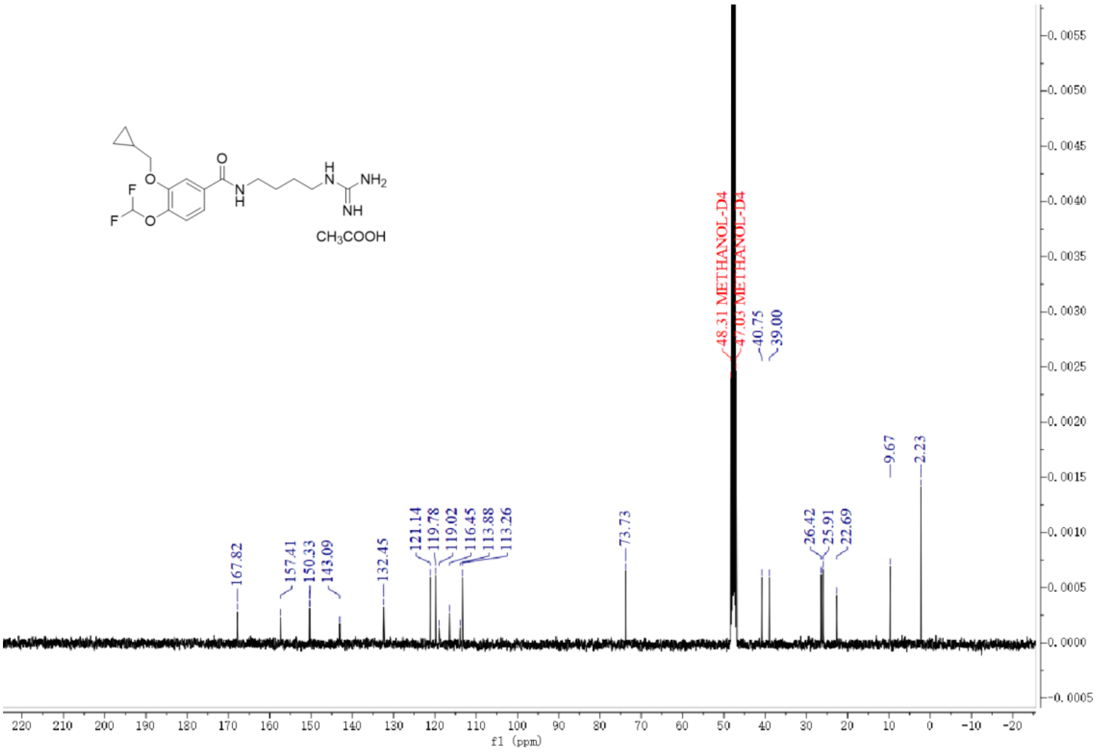

^{13}C NMR of compound A5(400MHz CD_3OD)

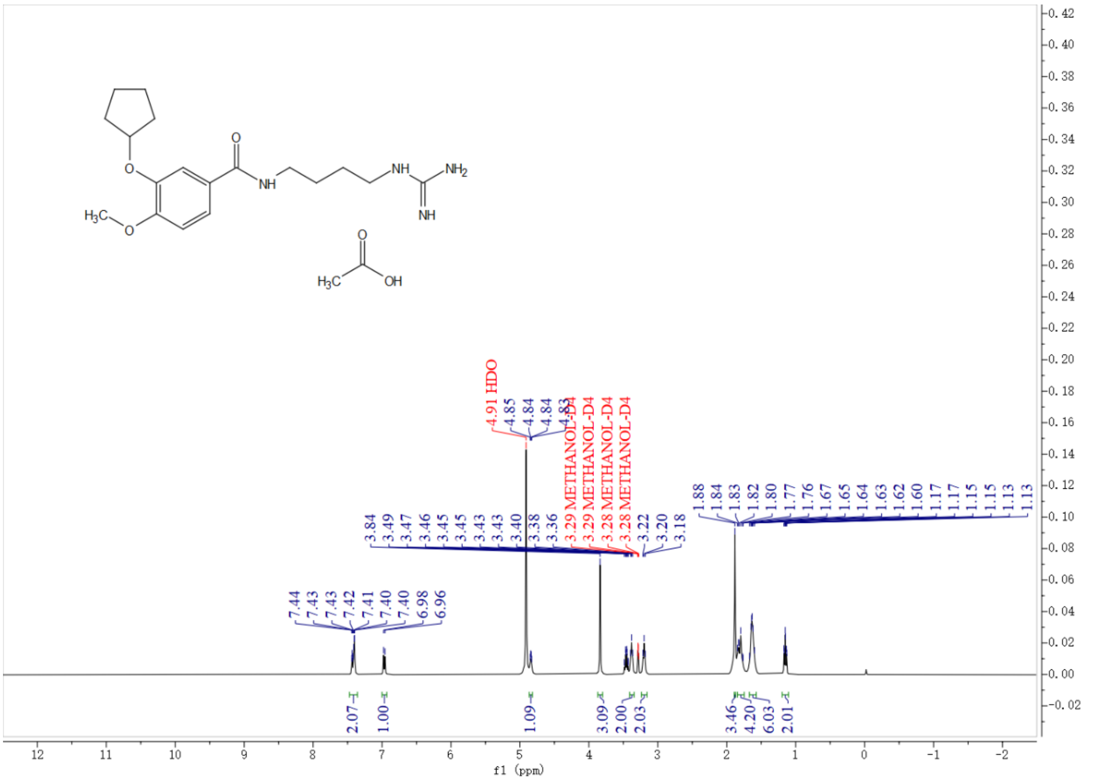

^{1}H NMR of compound A6(400MHz CD_3OD)

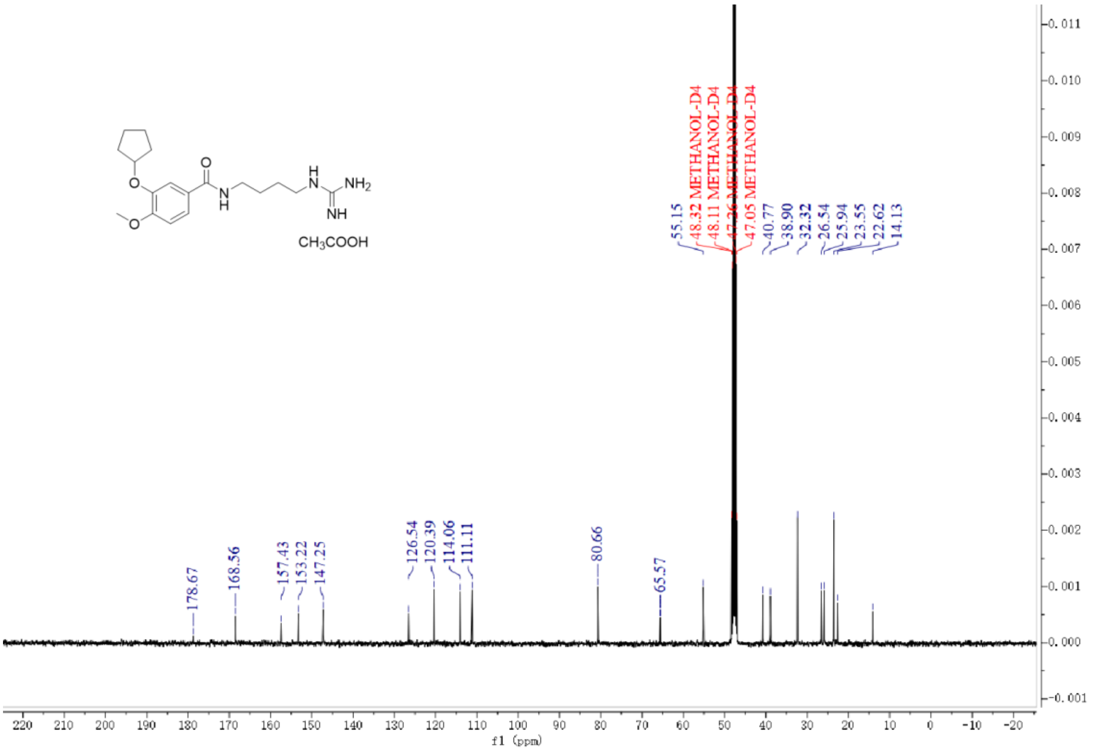

^{13}C NMR of compound A6(400MHz CD_3OD)

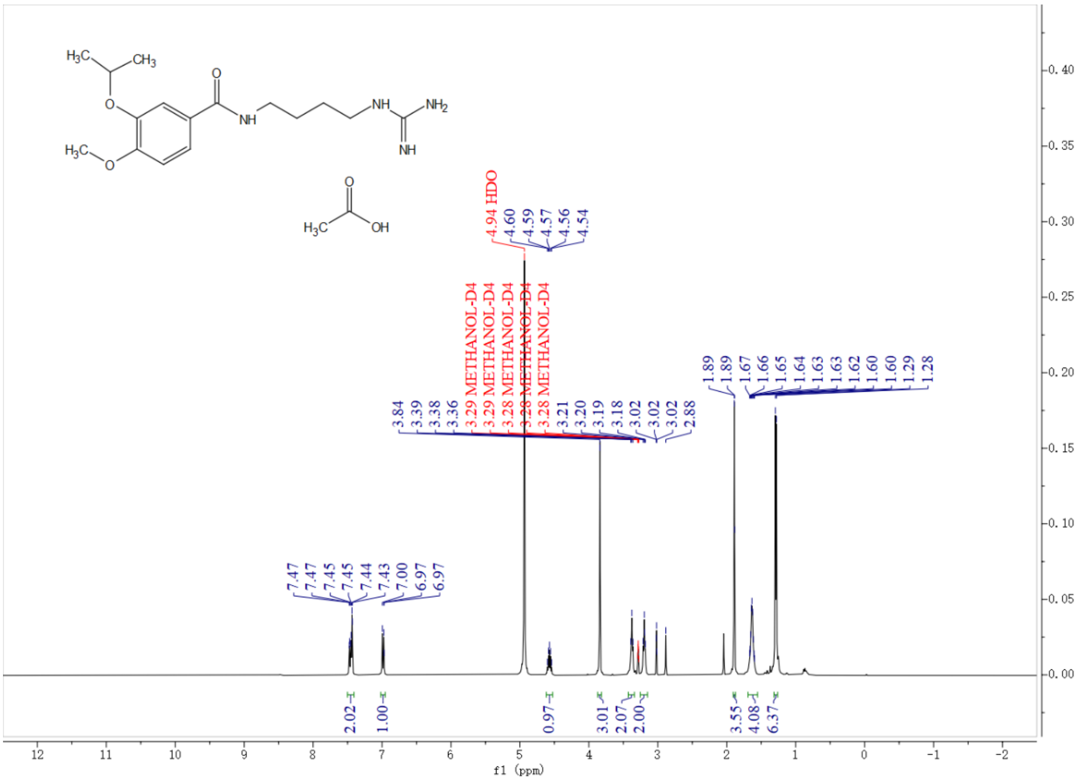

^{1}H NMR of compound A7(400MHz CD_3OD)

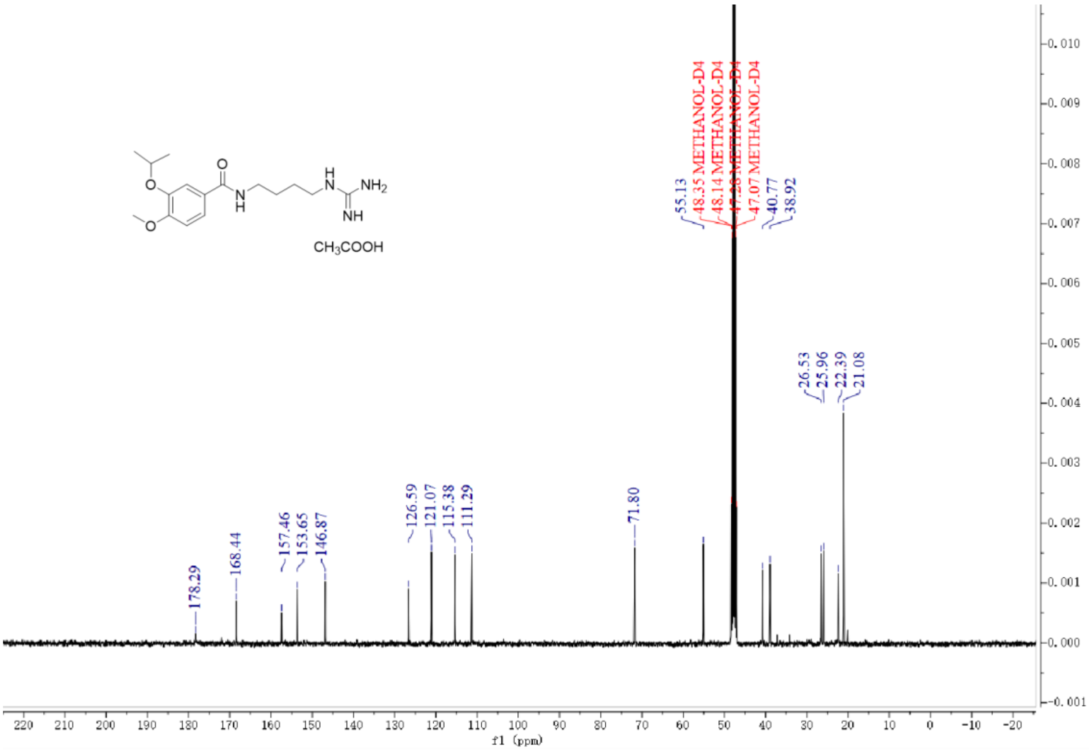

^{13}C NMR of compound A7(400MHz CD_3OD)

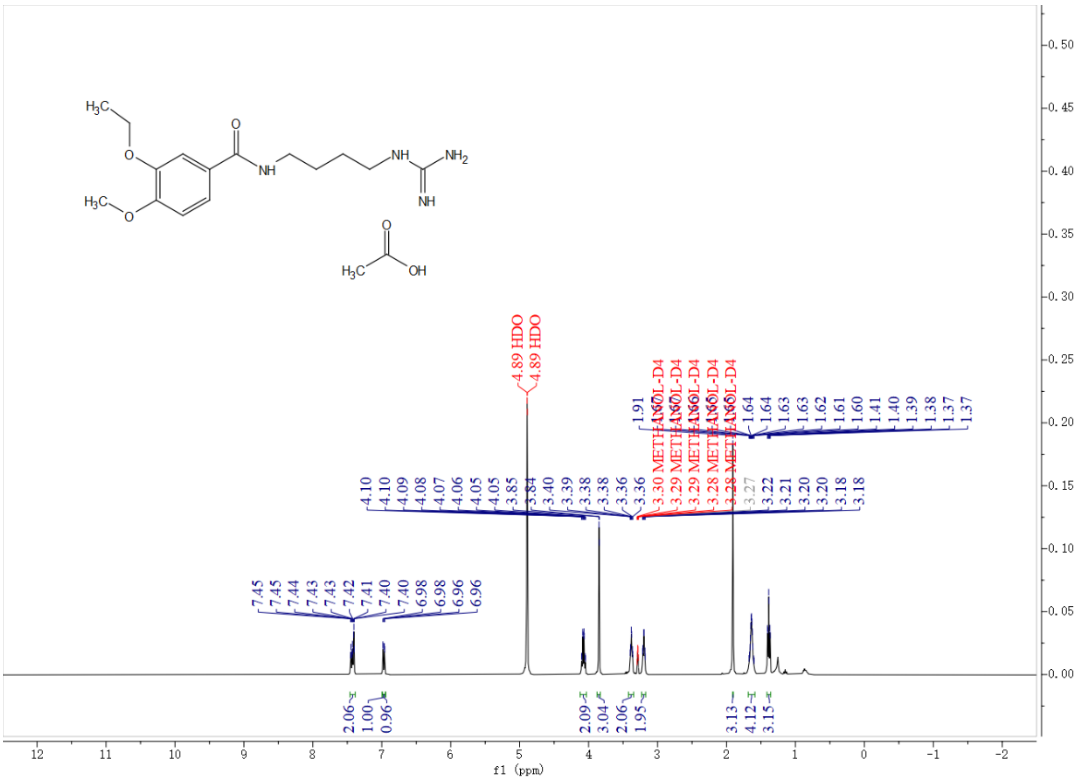

^{1}H NMR of compound A8(400MHz CD_3OD)

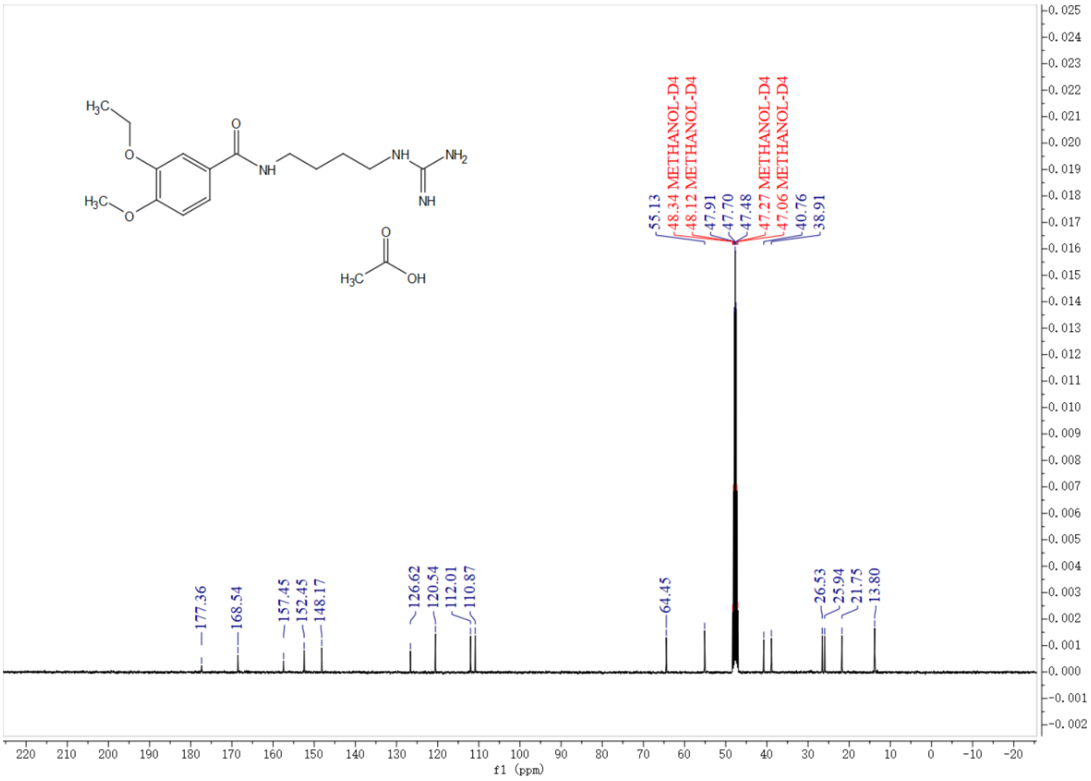

^{13}C NMR of compound A8(400MHz CD_3OD)

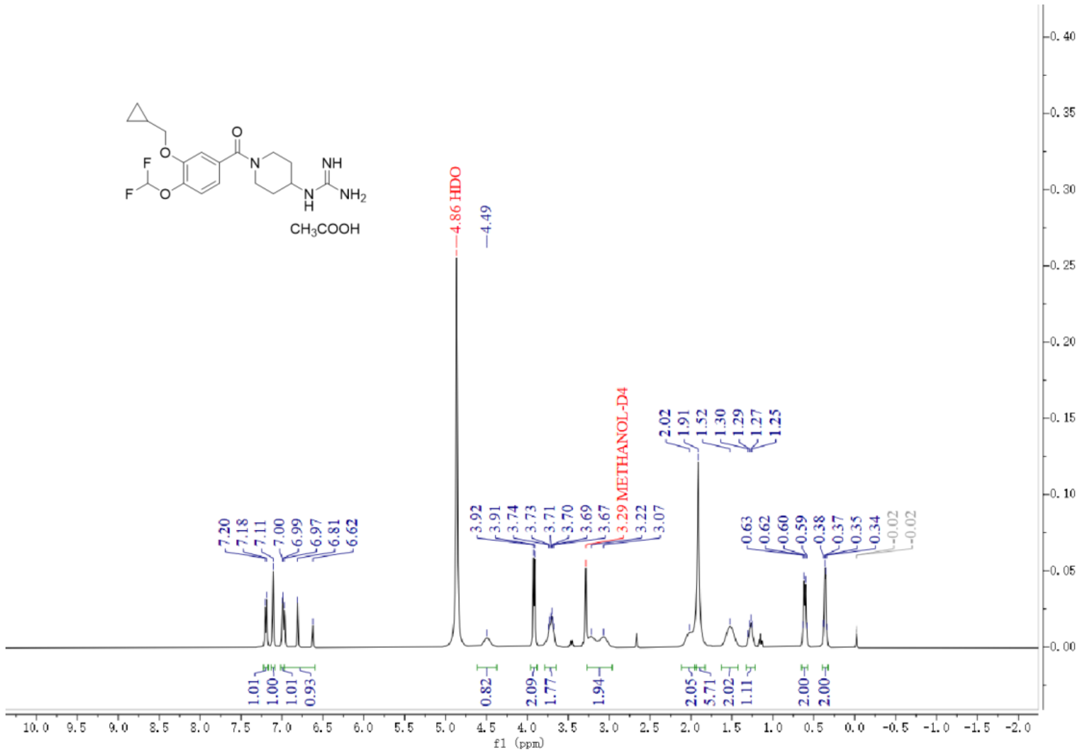

^{1}H NMR of compound A9(400MHz CD_3OD)

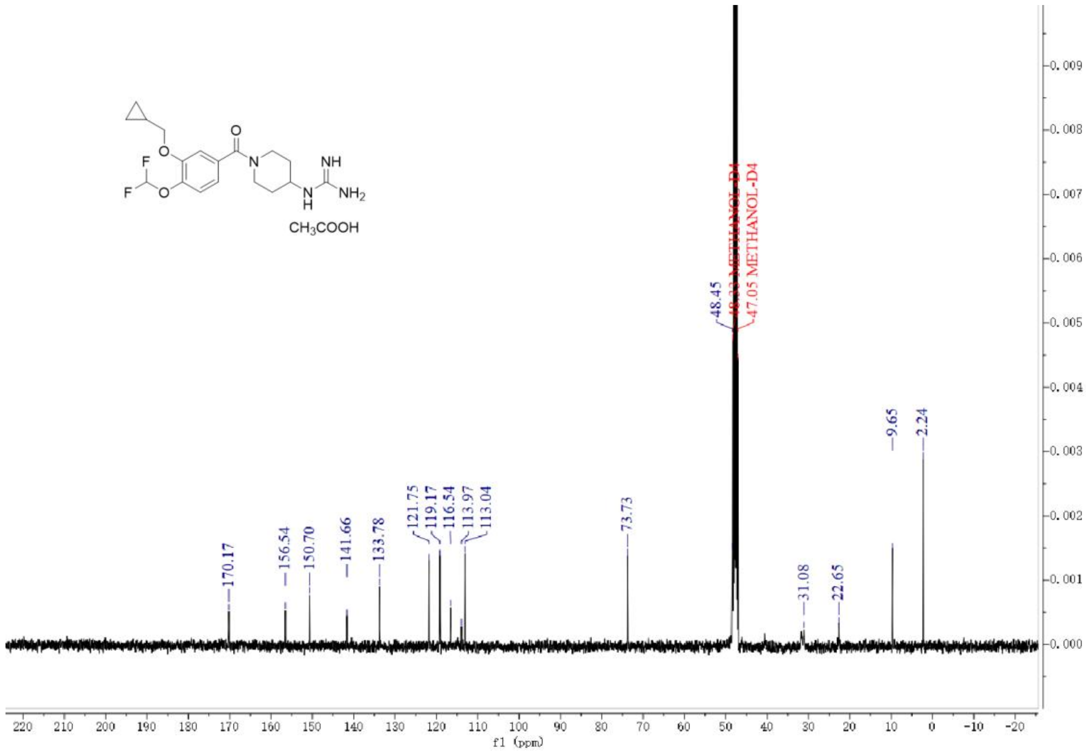

^{13}C NMR of compound A9(400MHz CD_3OD)

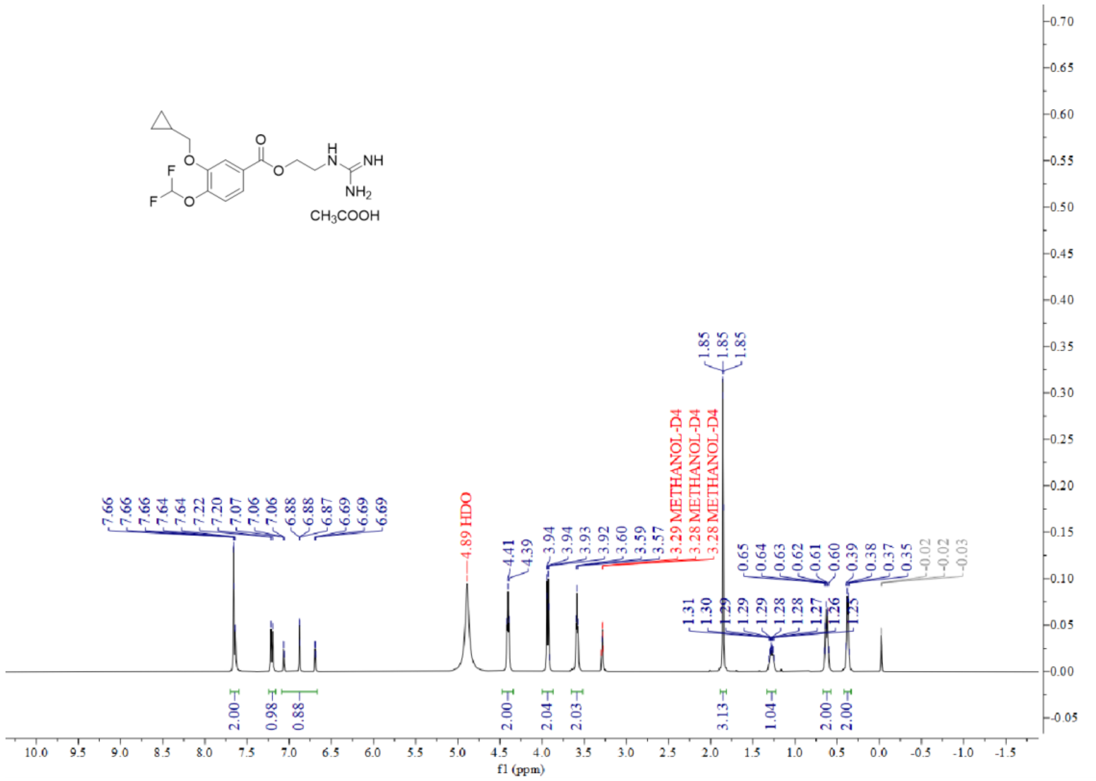

^{1}H NMR of compound B1(400MHz CD_3OD)

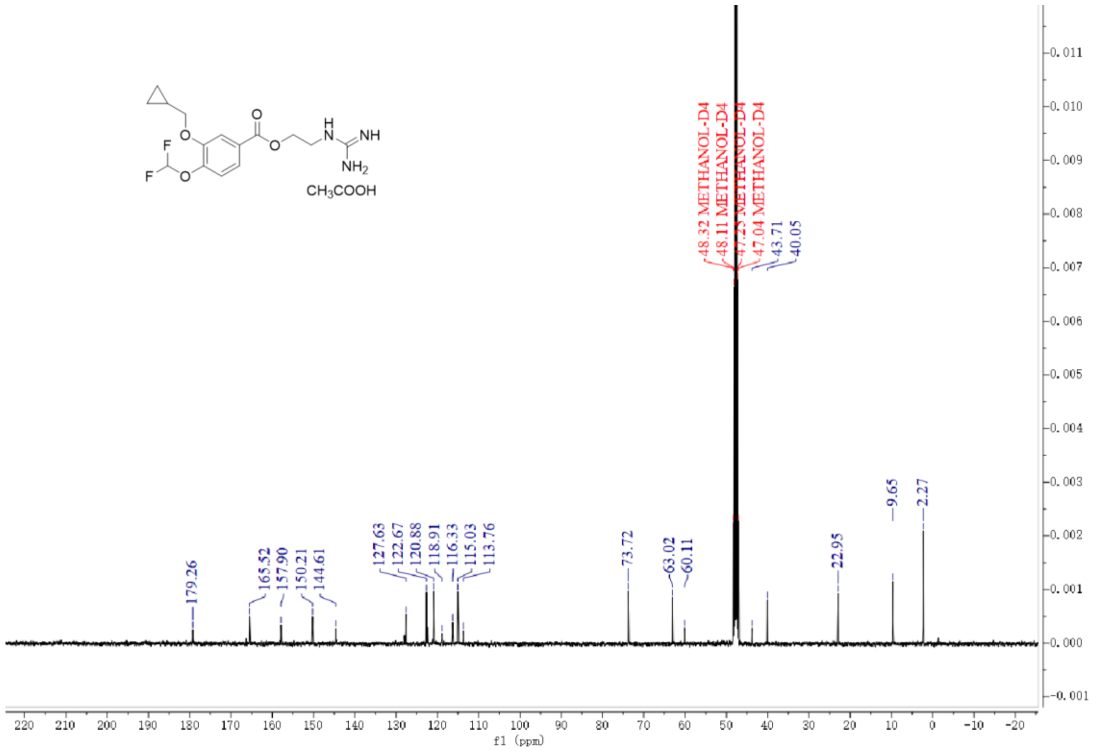

^{13}C NMR of compound B1(400MHz CD_3OD)

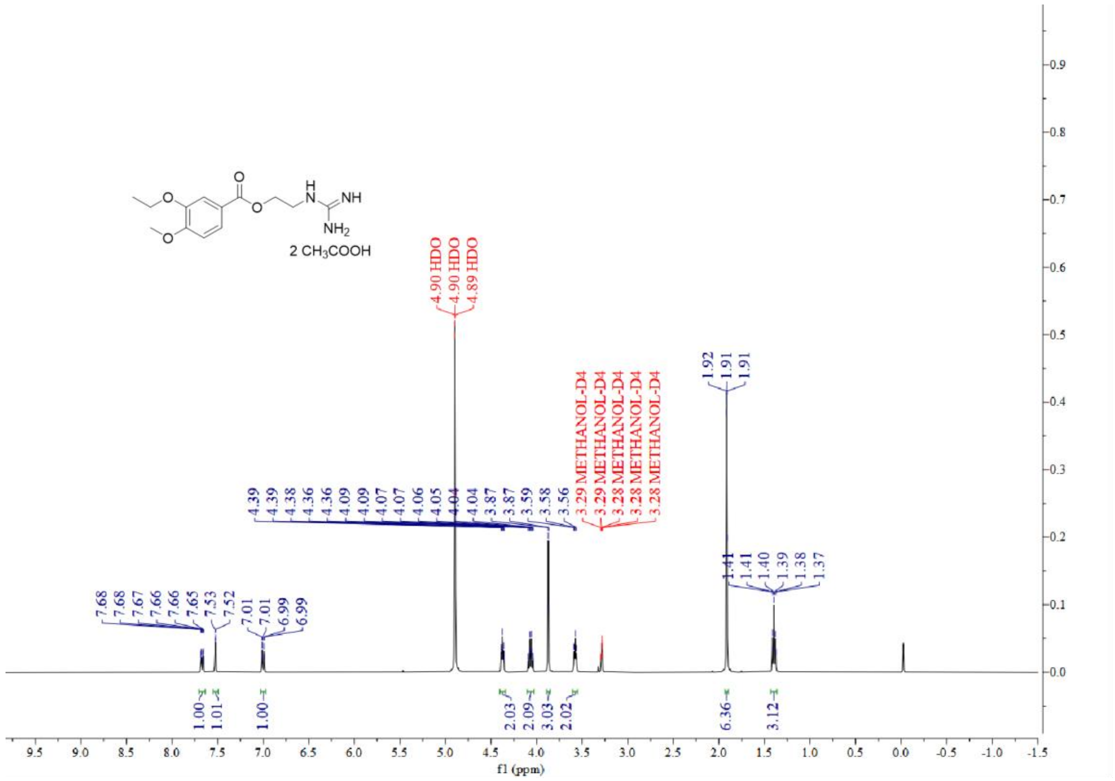

^{1}H NMR of compound B2(400MHz CD_3OD)

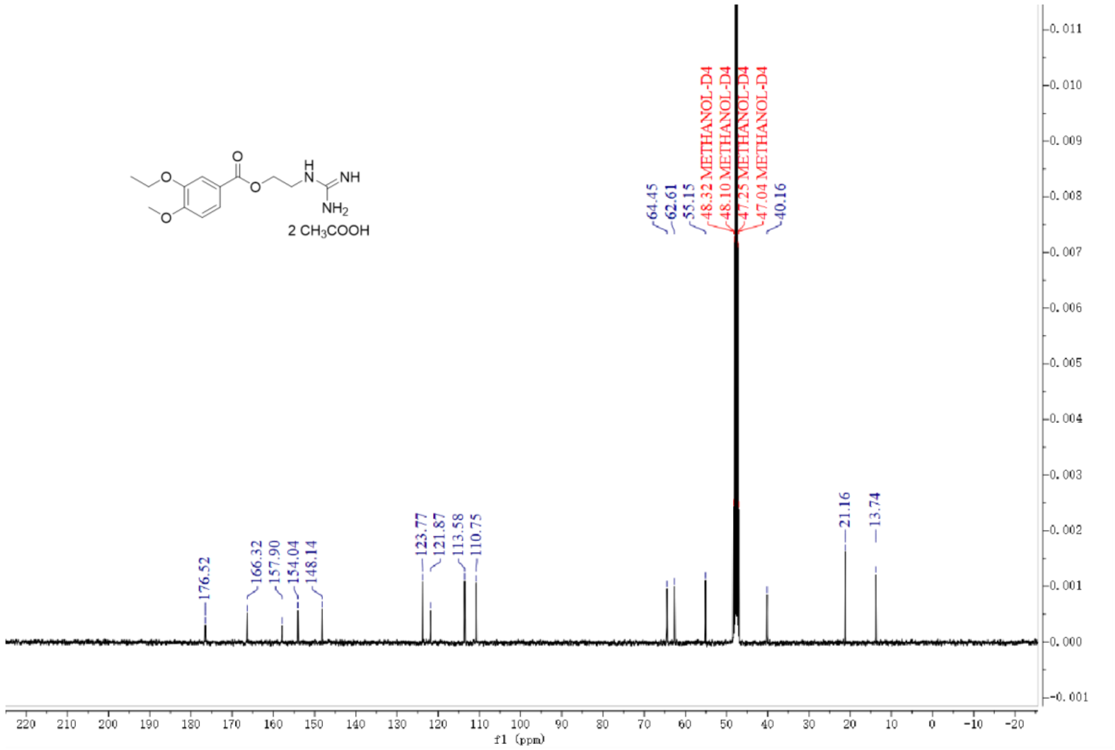

^{13}C NMR of compound B2(400MHz CD_3OD)

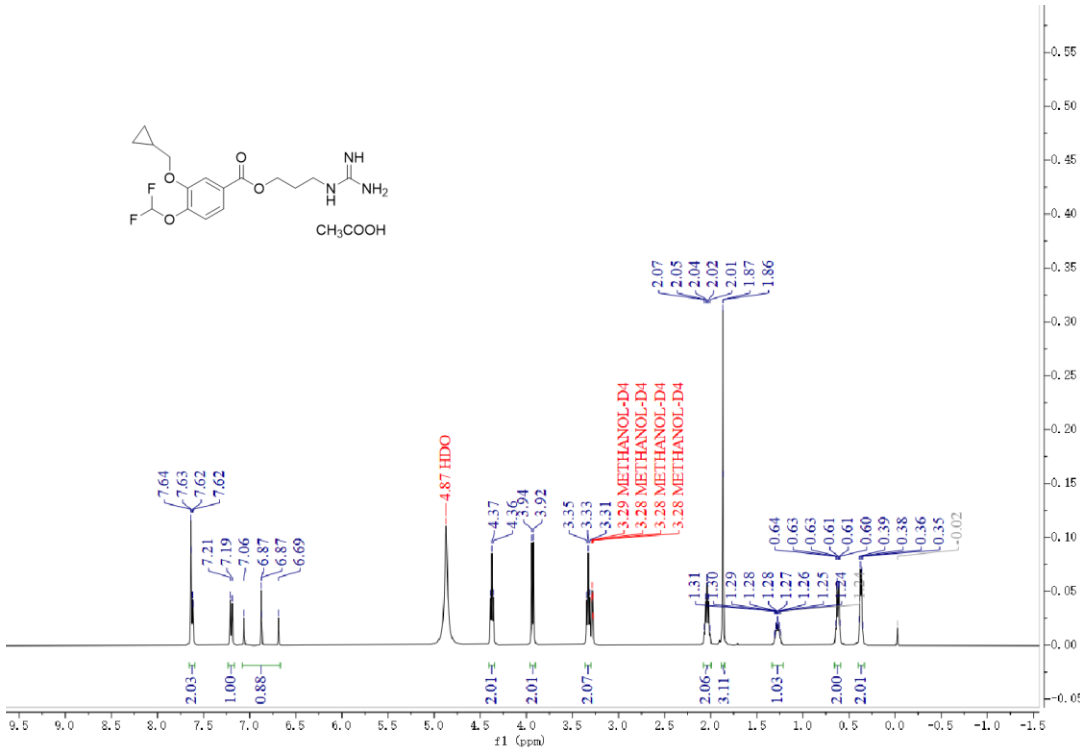

^{1}H NMR of compound B3(400MHz CD_3OD)

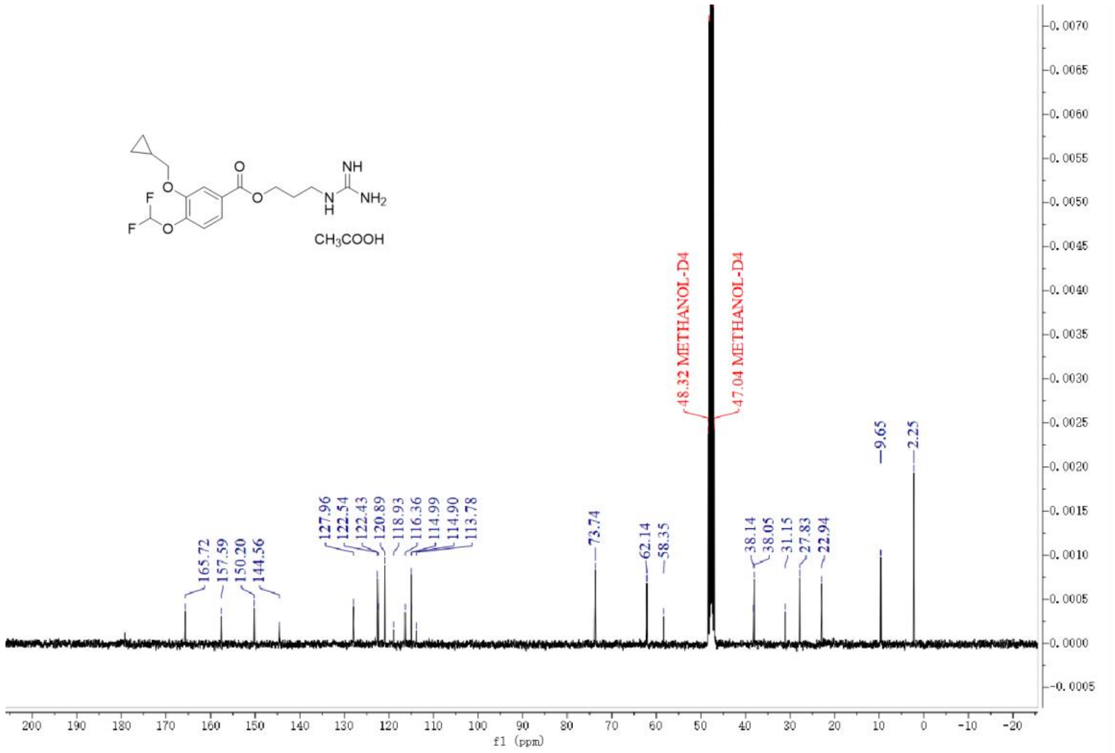

^{13}C NMR of compound B3(400MHz CD_3OD)

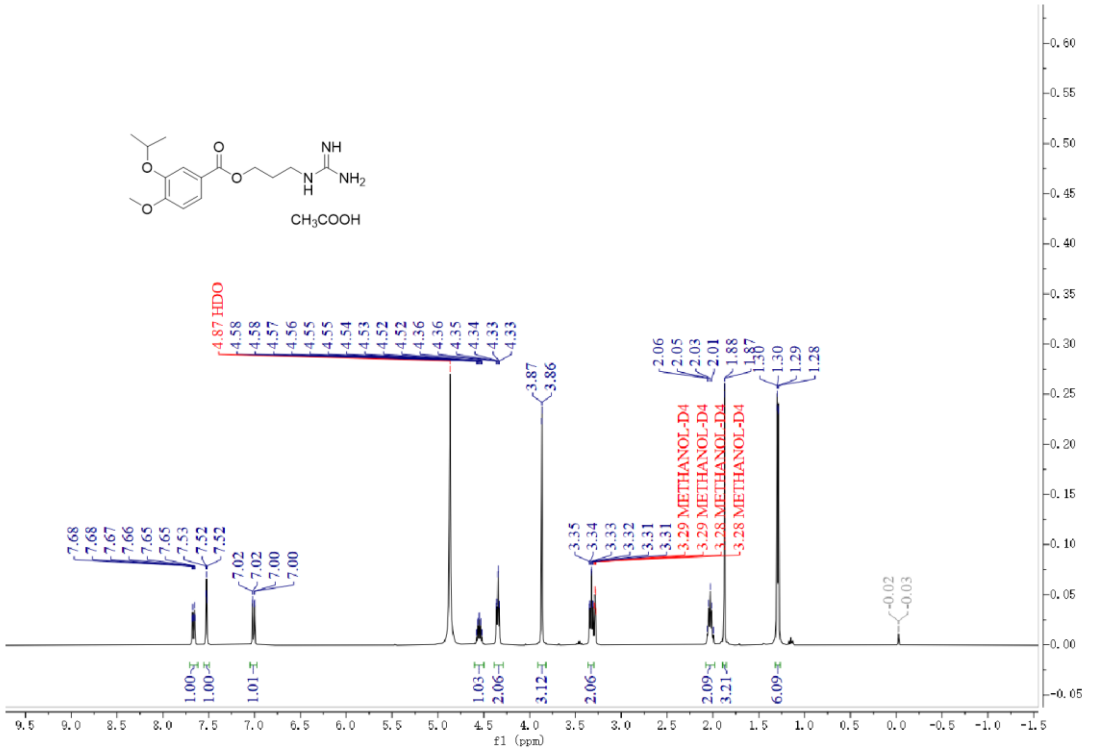

^{1}H NMR of compound B4(400MHz CD_3OD)

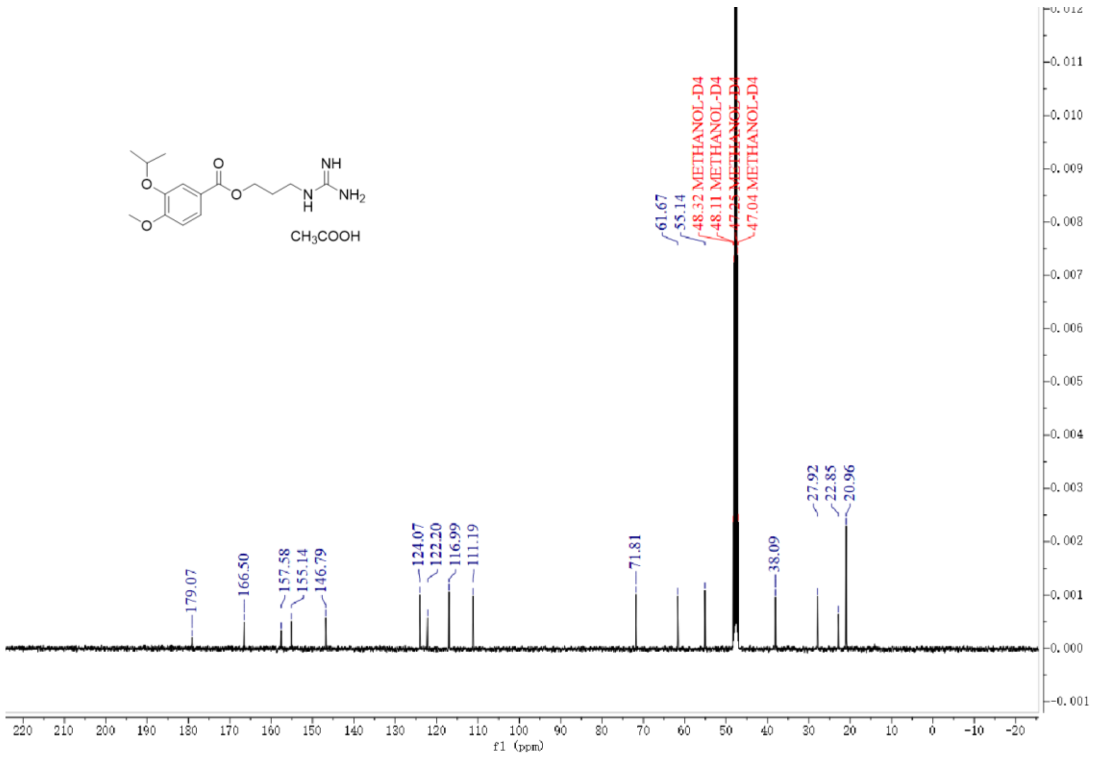

^{13}C NMR of compound B4(400MHz CD_3OD)

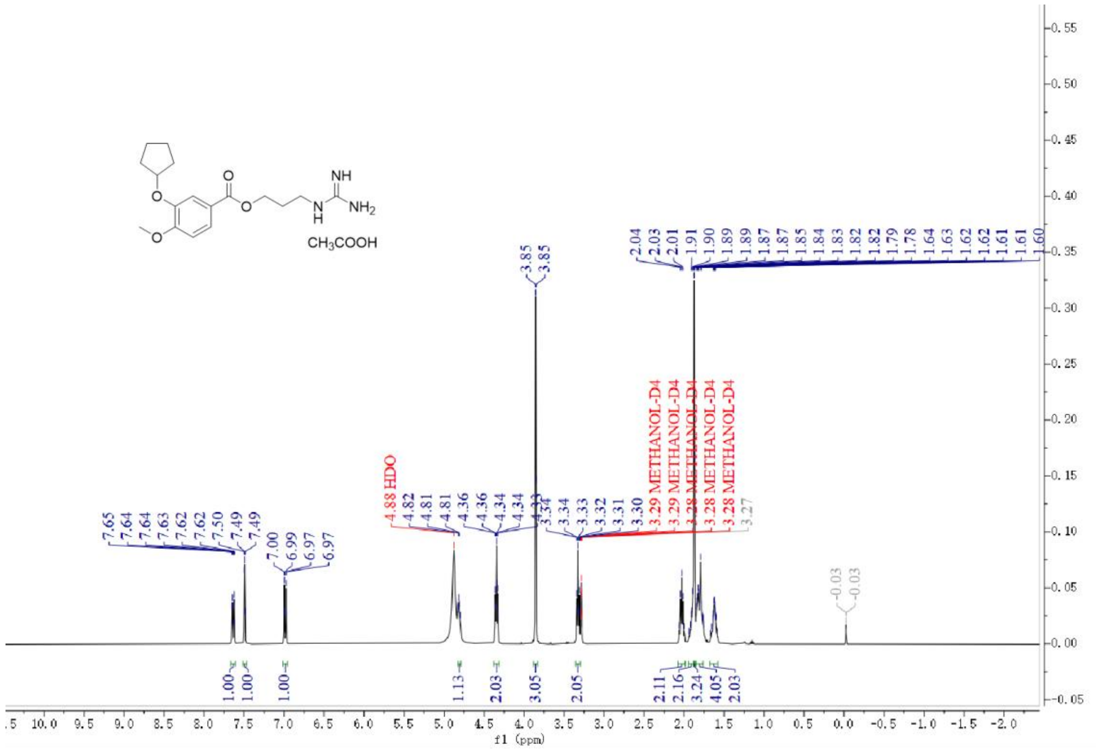

^{1}H NMR of compound B5(400MHz CD_3OD)

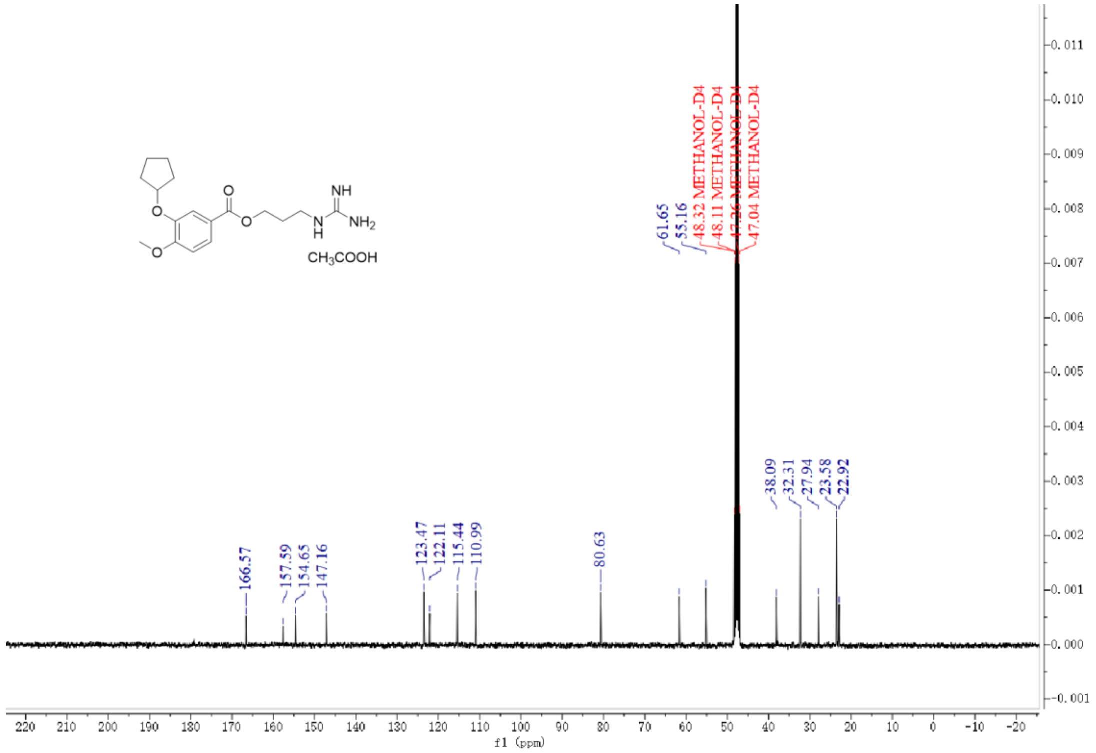

^{13}C NMR of compound B5(400MHz CD_3OD)

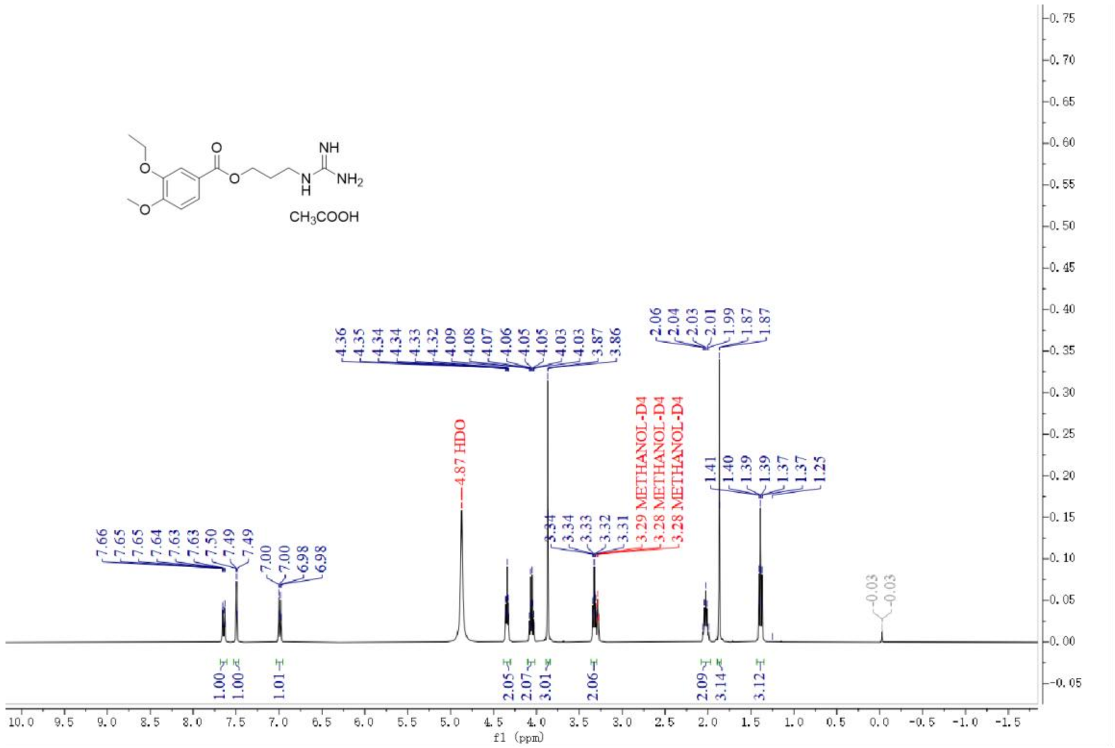

^{1}H NMR of compound B6(400MHz CD_3OD)

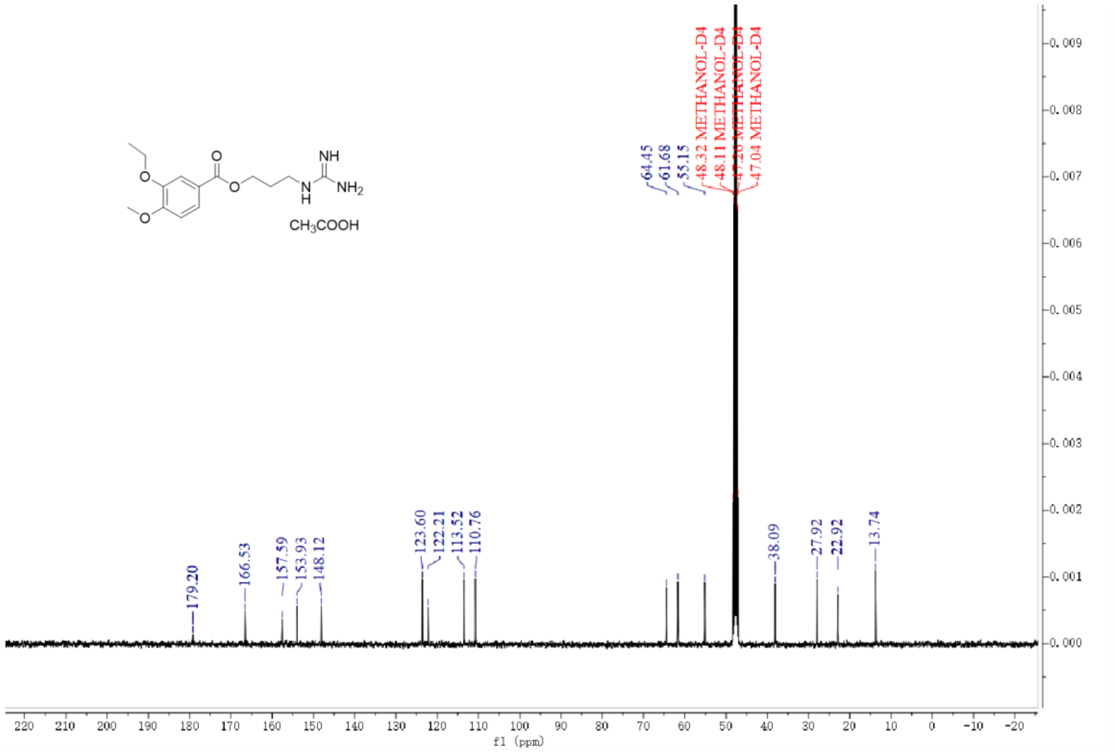

^{13}C NMR of compound B6(400MHz CD_3OD)

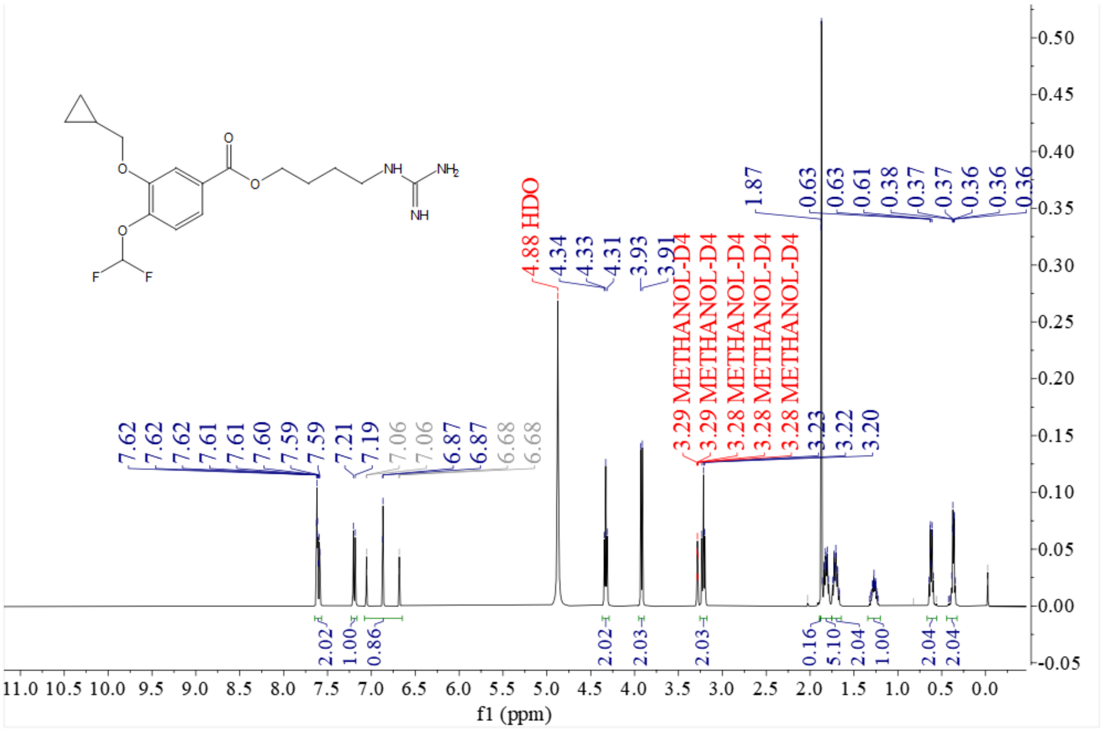

^{1}H NMR of compound B7(400MHz CD_3OD)

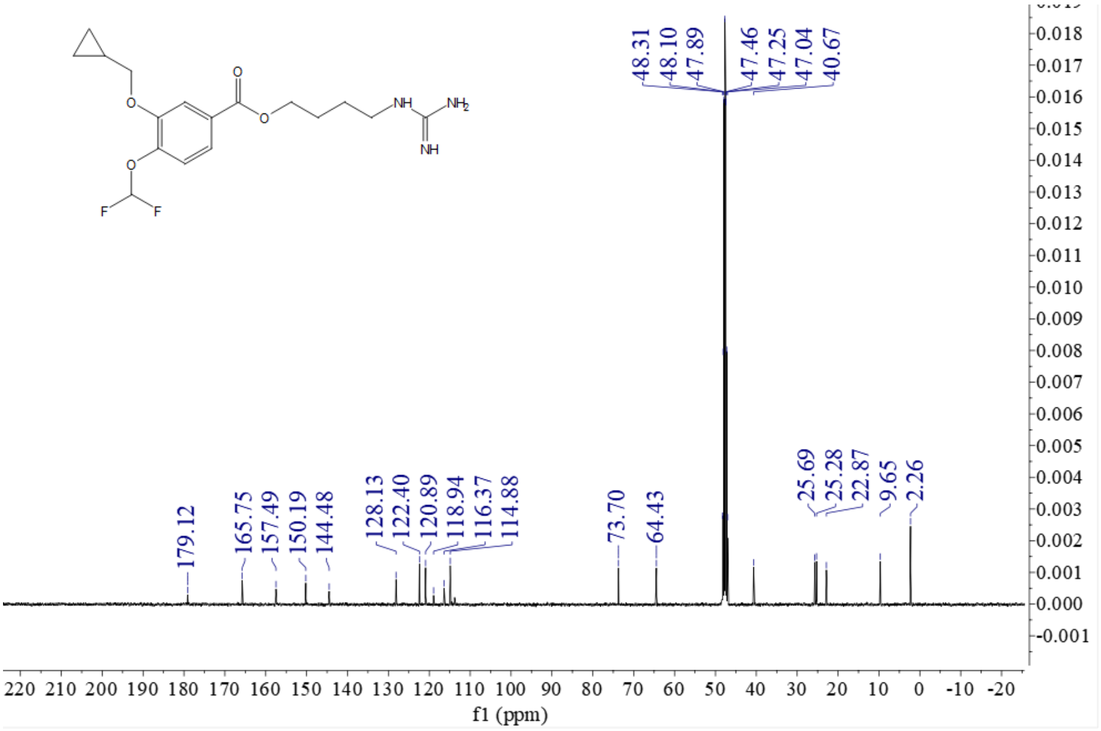

^{13}C NMR of compound B7(400MHz CD_3OD)

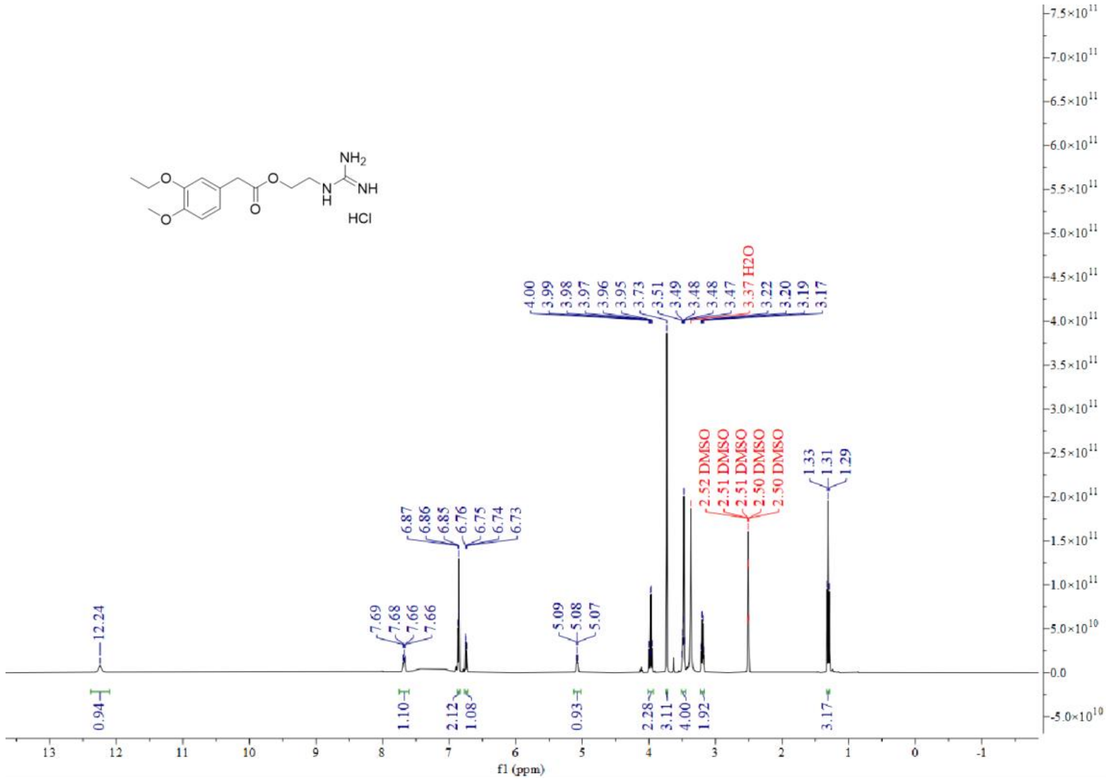

^{1}H NMR of compound C1(400MHz DMSO-*d*6)

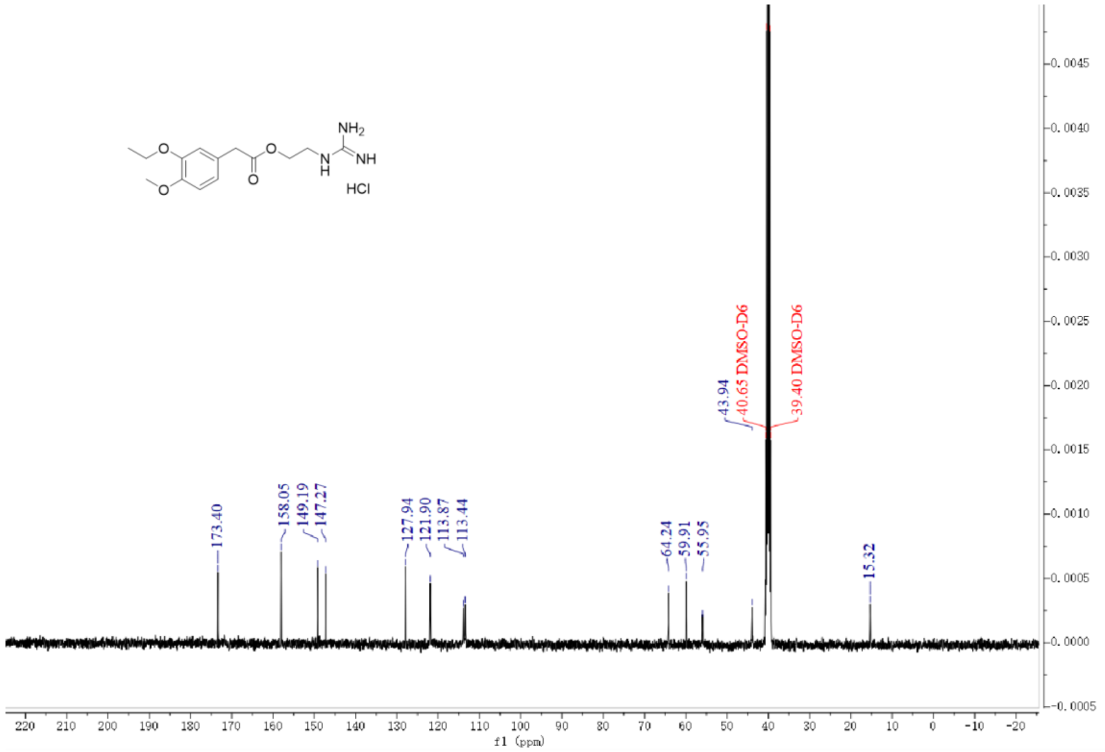

^{13}C NMR of compound C1(400MHz DMSO-*d*6)

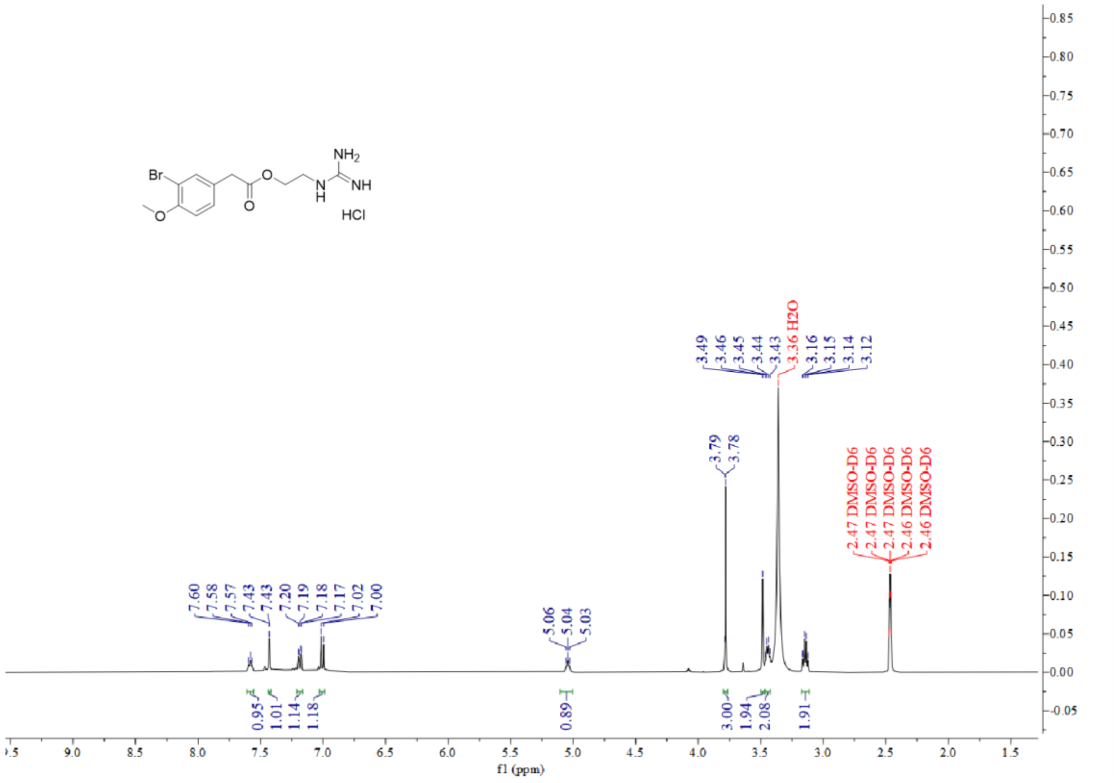

^{1}H NMR of compound C2(400MHz DMSO-*d*6)

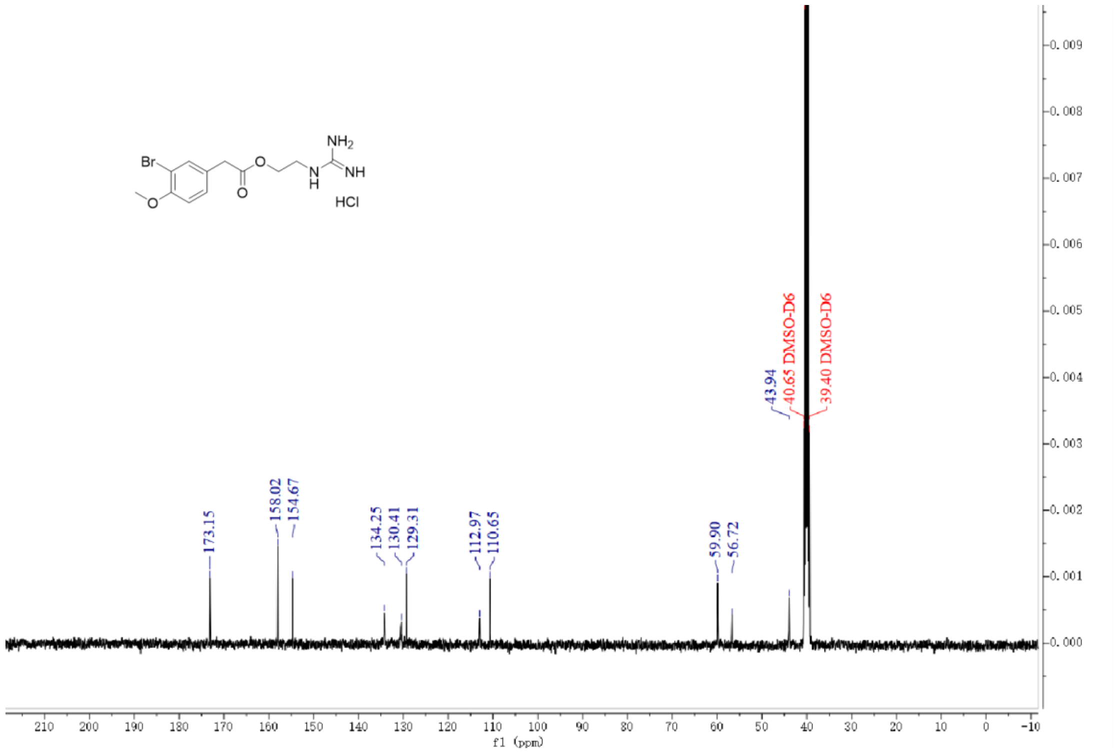

^{13}C NMR of compound C2(400MHz DMSO-*d*6)

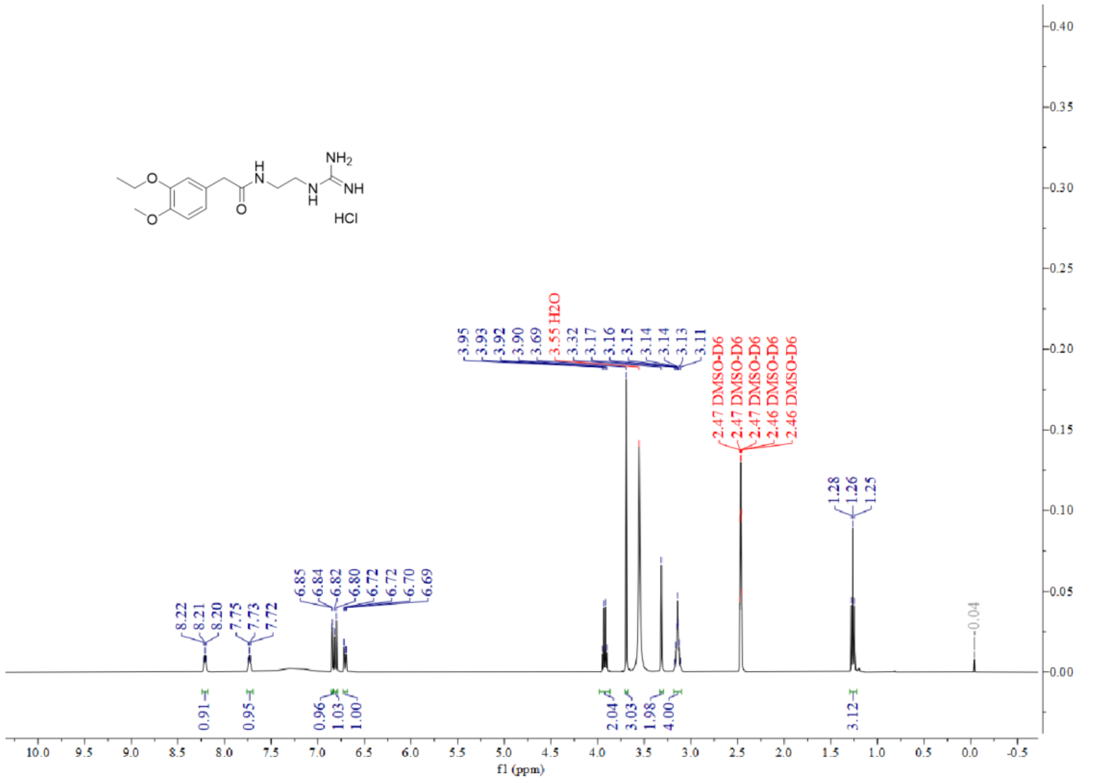

^{1}H NMR of compound C3(400MHz DMSO-*d*6)

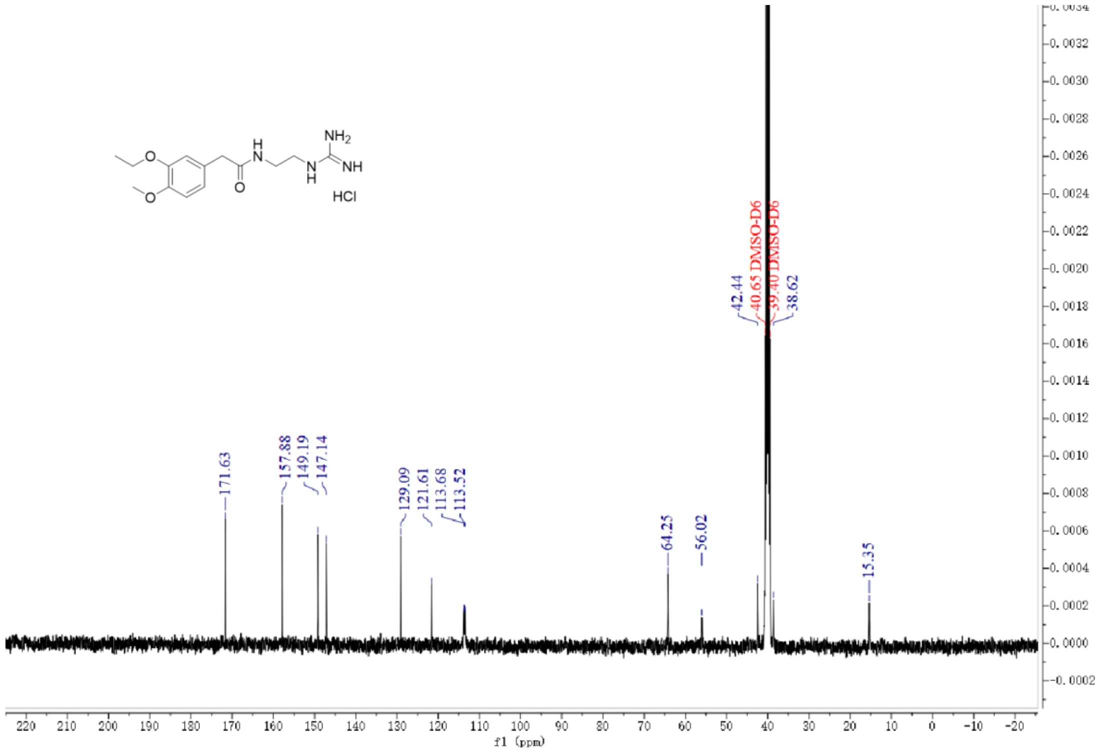

^{13}C NMR of compound C3(400MHz DMSO-*d*6)

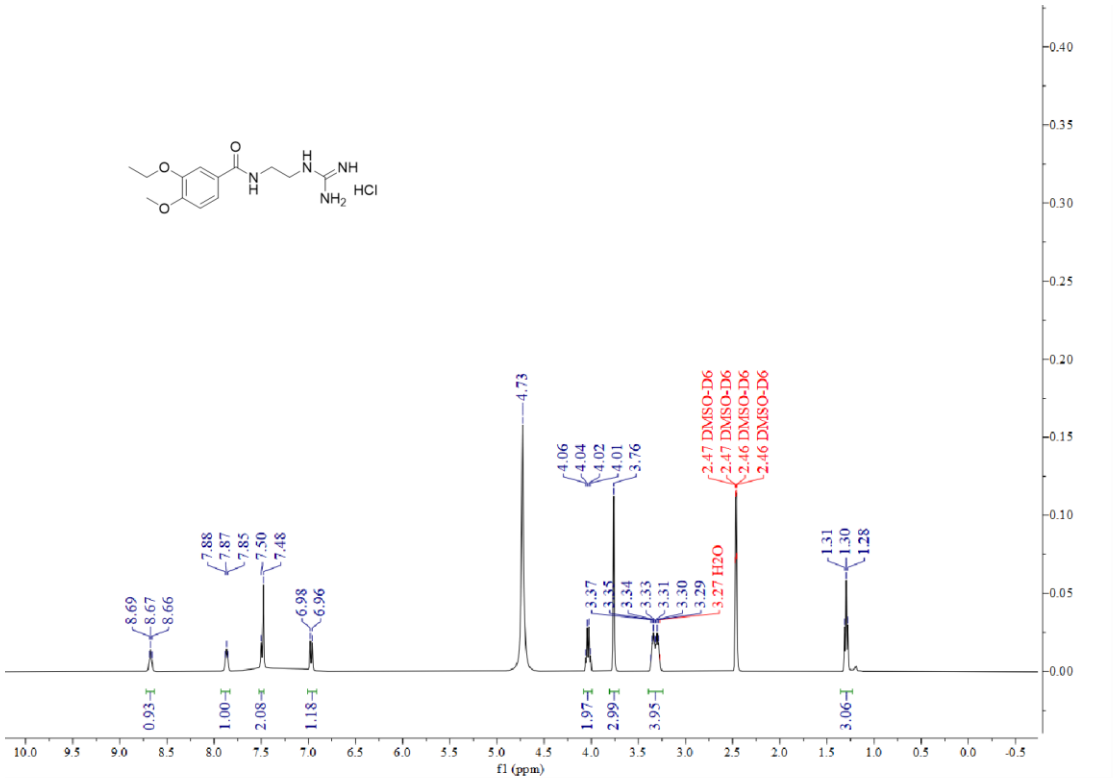

^{1}H NMR of compound C4(400MHz DMSO-*d*6)

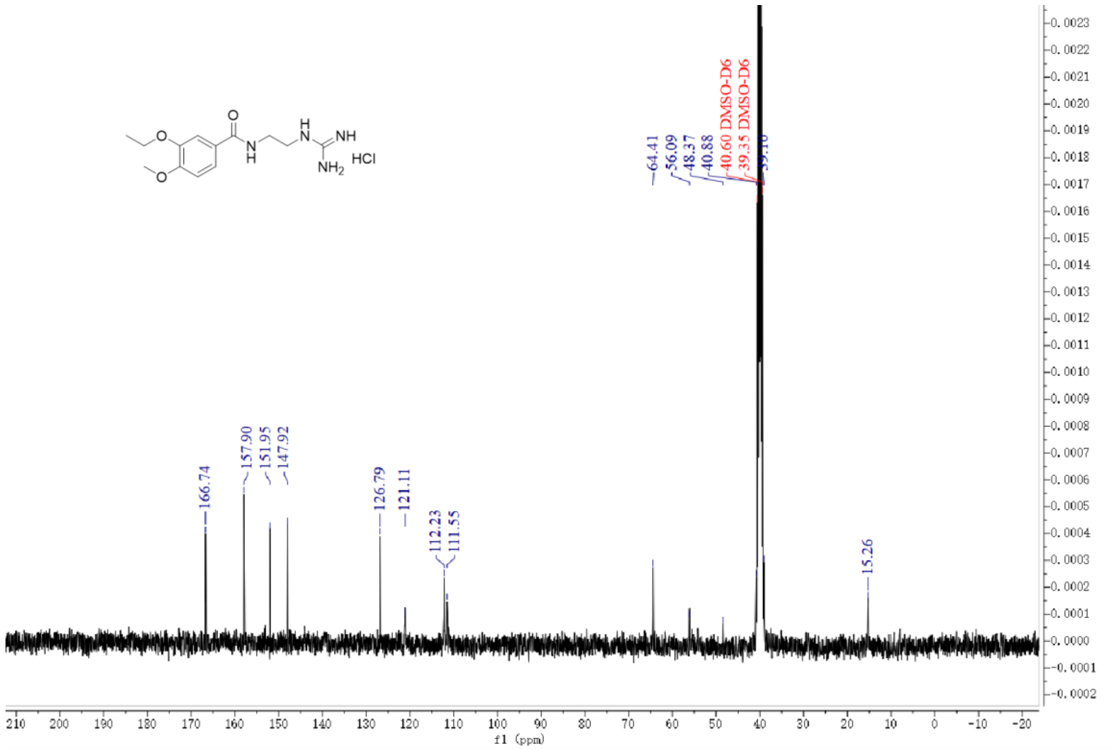

^{13}C NMR of compound C4(400MHz DMSO-*d*6)

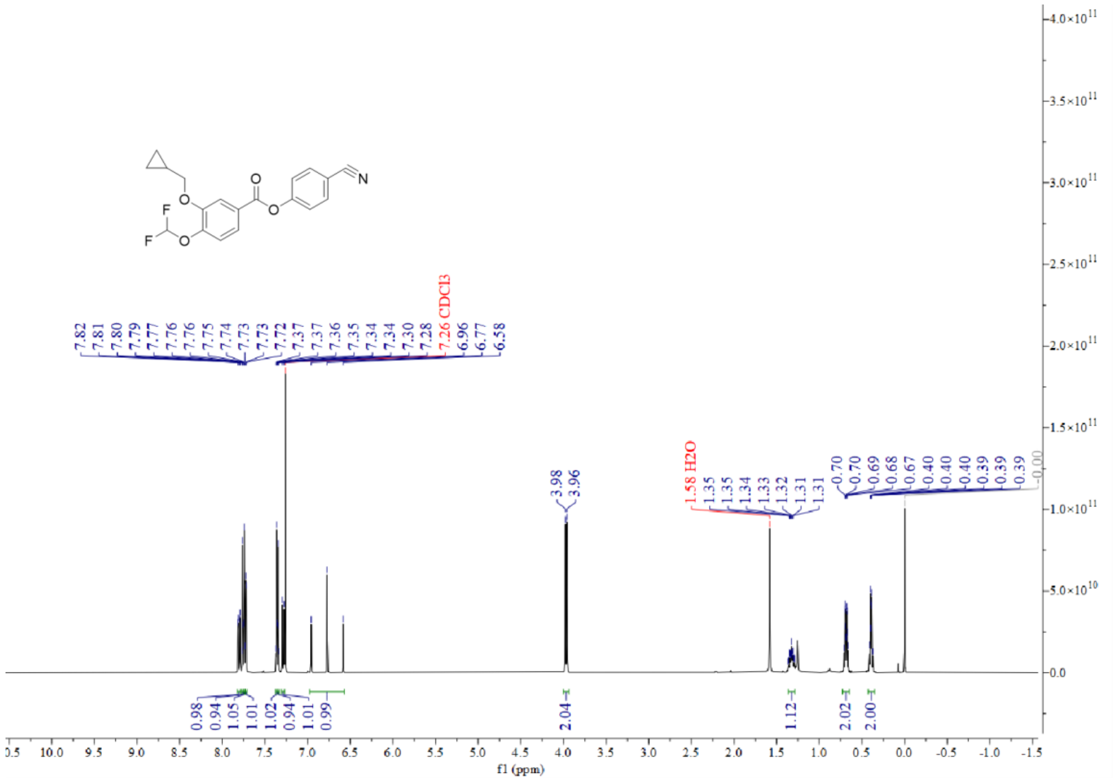

^{1}H NMR of compound D1(400MHz $CDCl_3$)

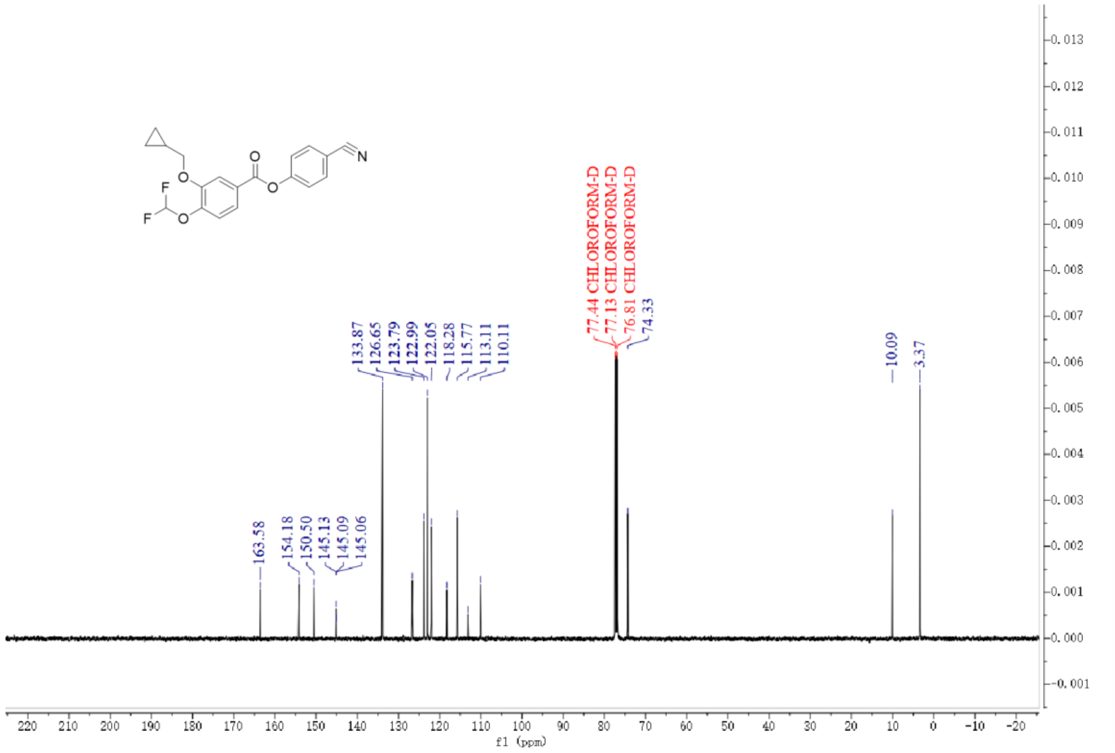

^{13}C NMR of compound D1(400MHz $CDCl_3$)

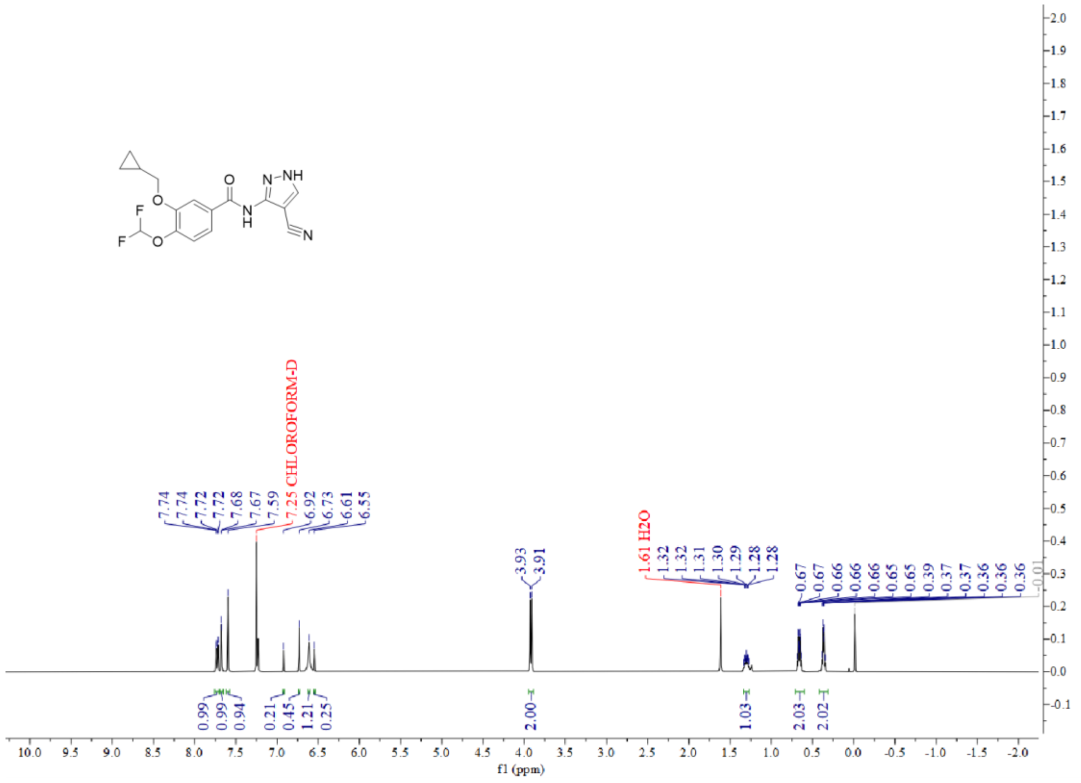

^{1}H NMR of compound D2(400MHz $CDCl_3$)

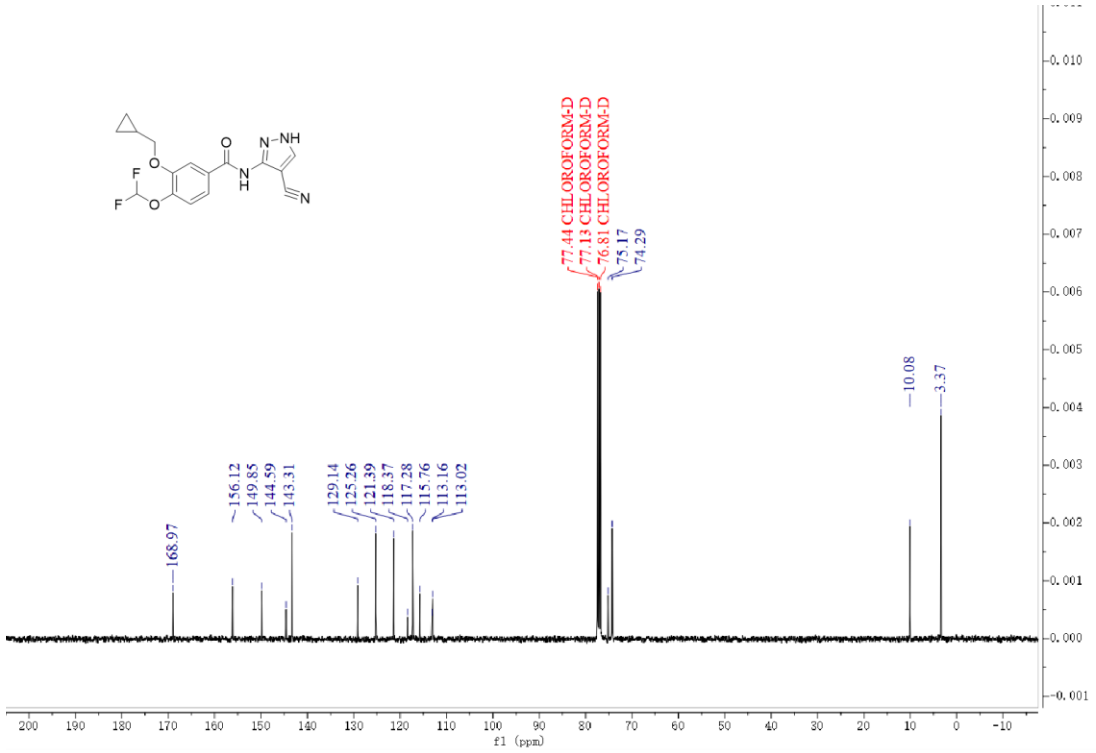

^{13}C NMR of compound D2(400MHz $CDCl_3$)

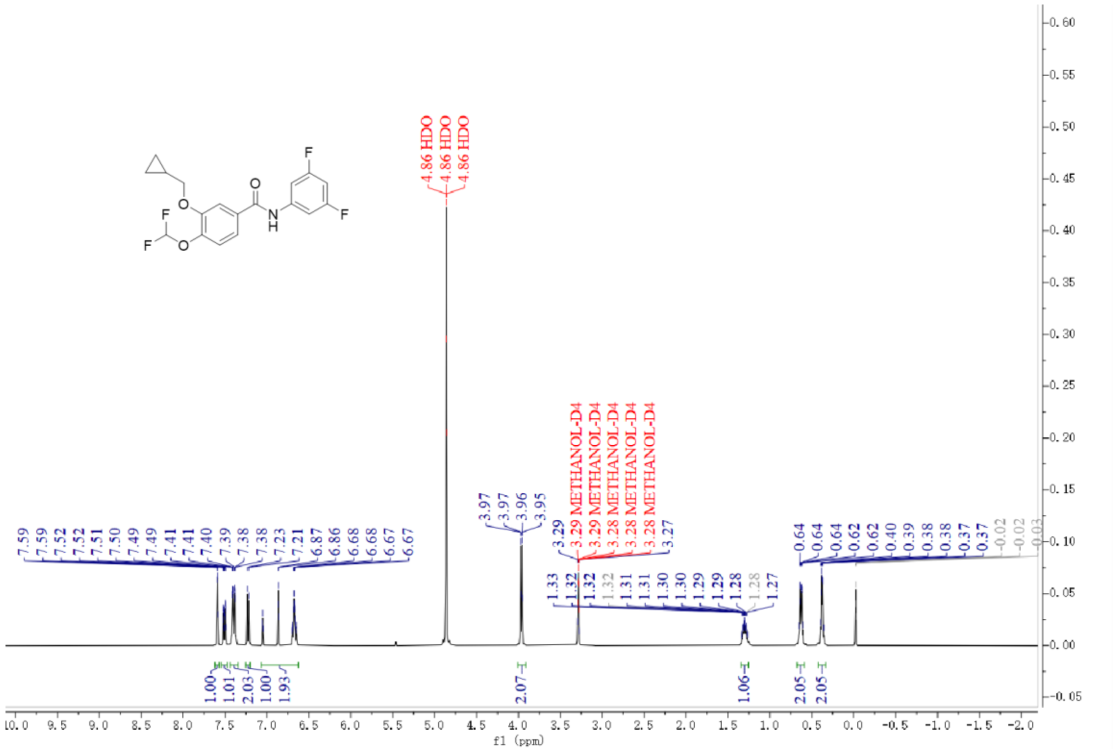

^{1}H NMR of compound D3(400MHz $CDCl_3$)

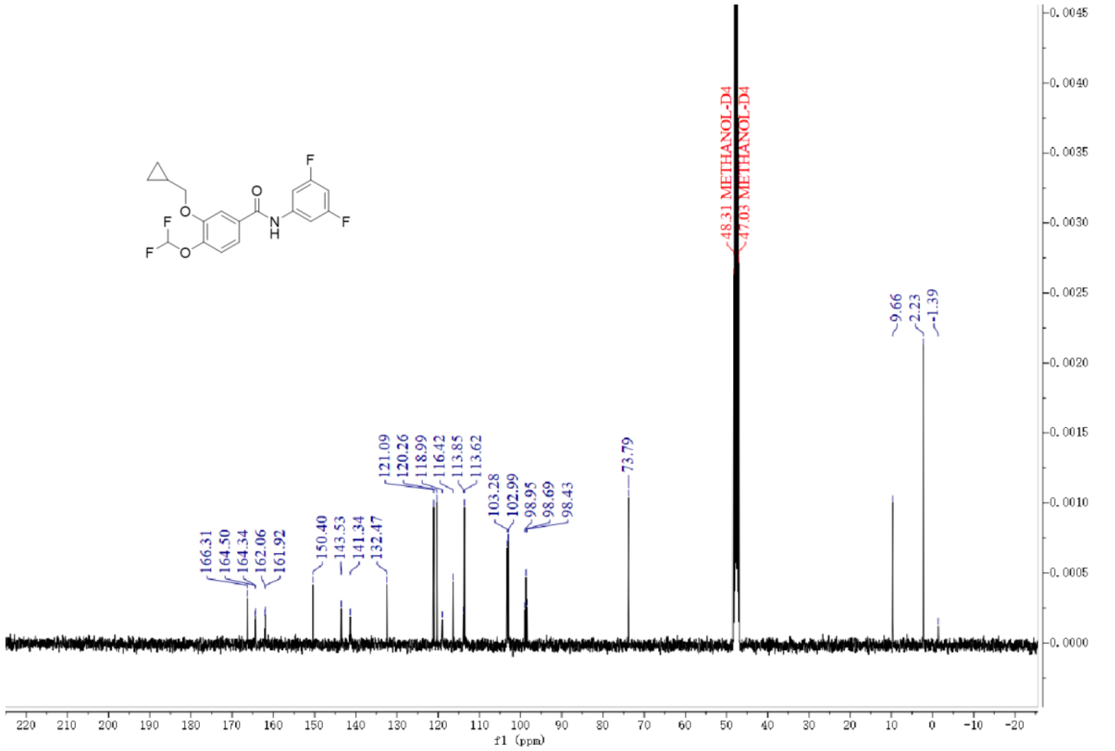

^{13}C NMR of compound D3(400MHz $CDCl_3$)

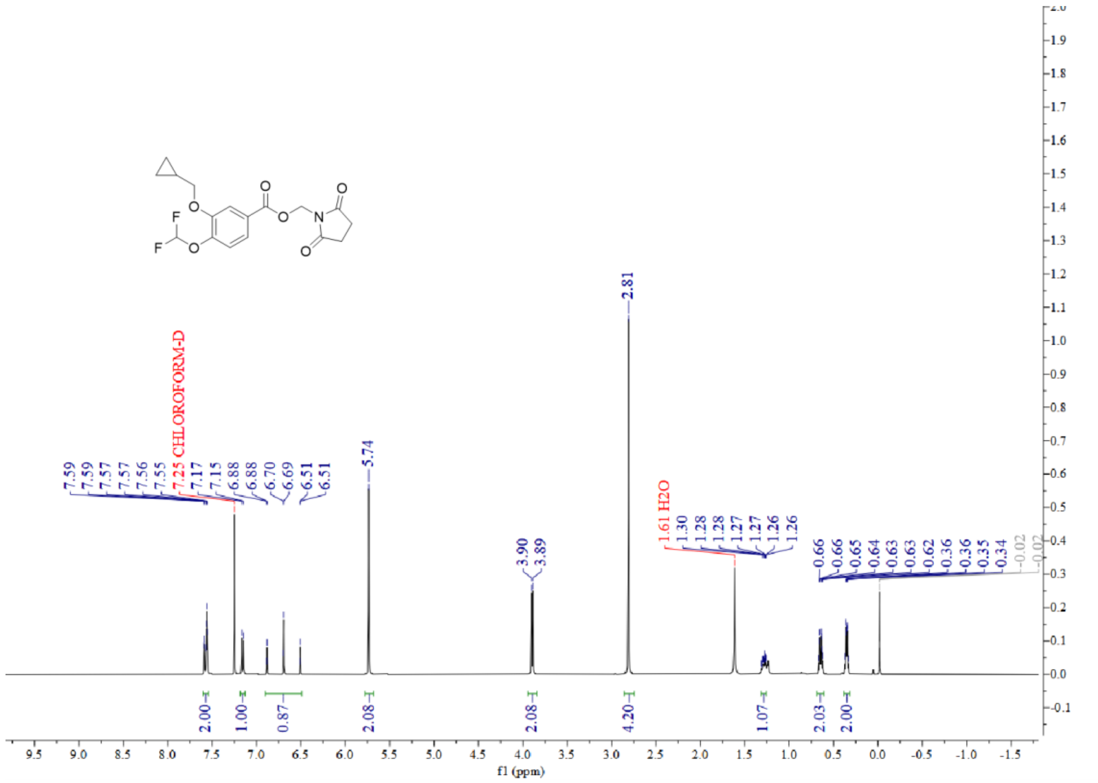

^{1}H NMR of compound D4(400MHz $CDCl_3$)

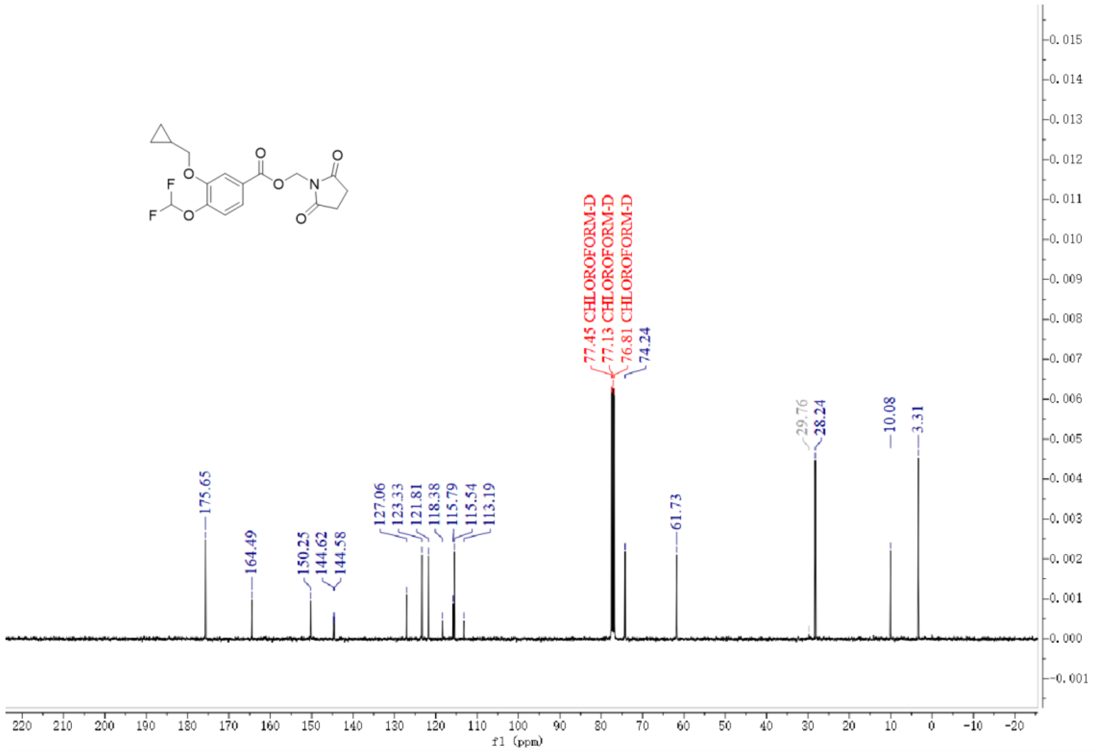

^{13}C NMR of compound D4(400MHz $CDCl_3$)

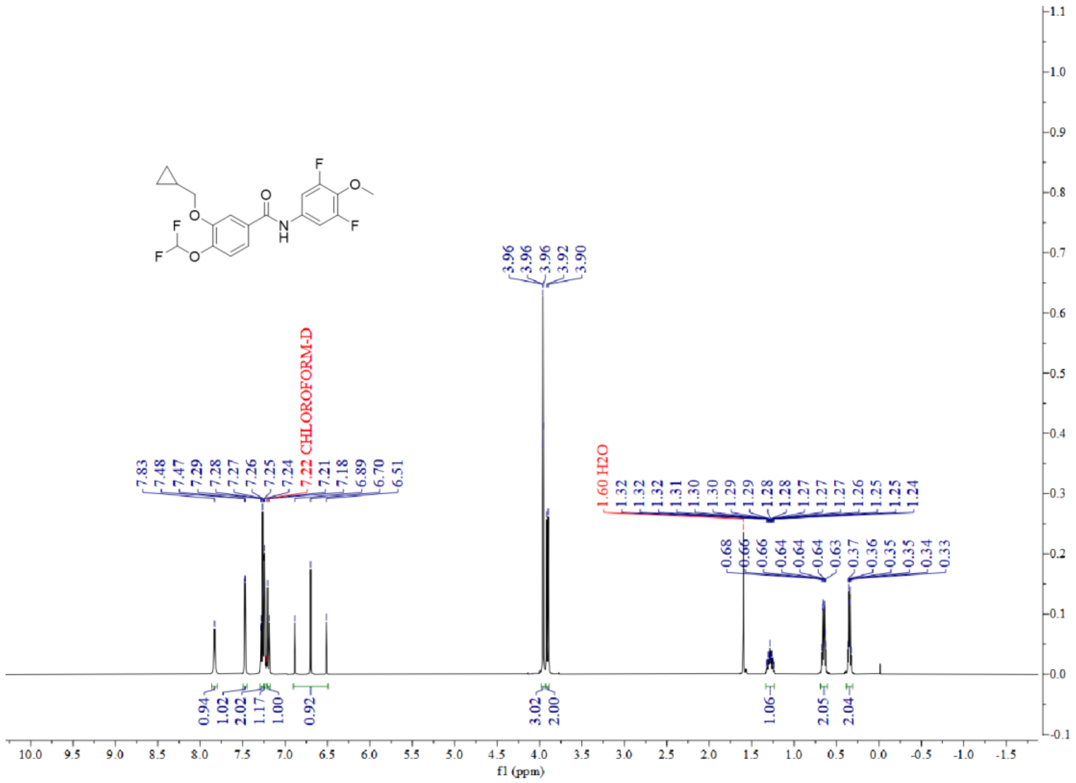

^{1}H NMR of compound D5(400MHz $CDCl_3$)

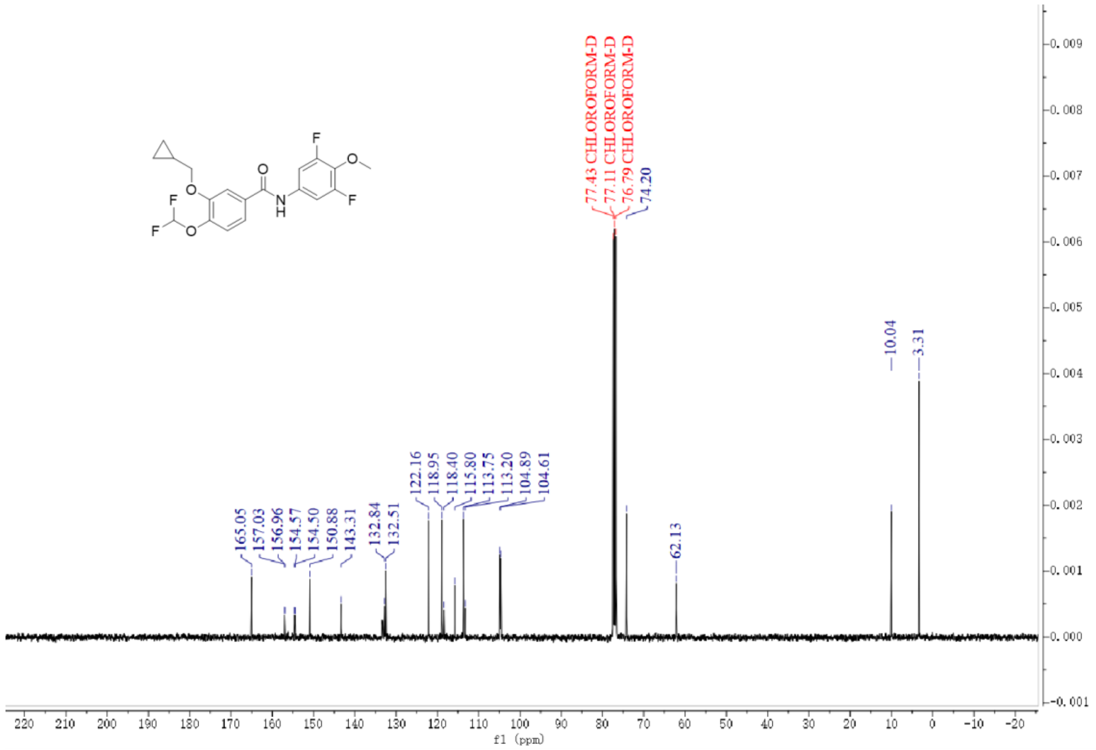

^{13}C NMR of compound D5(400MHz $CDCl_3$)

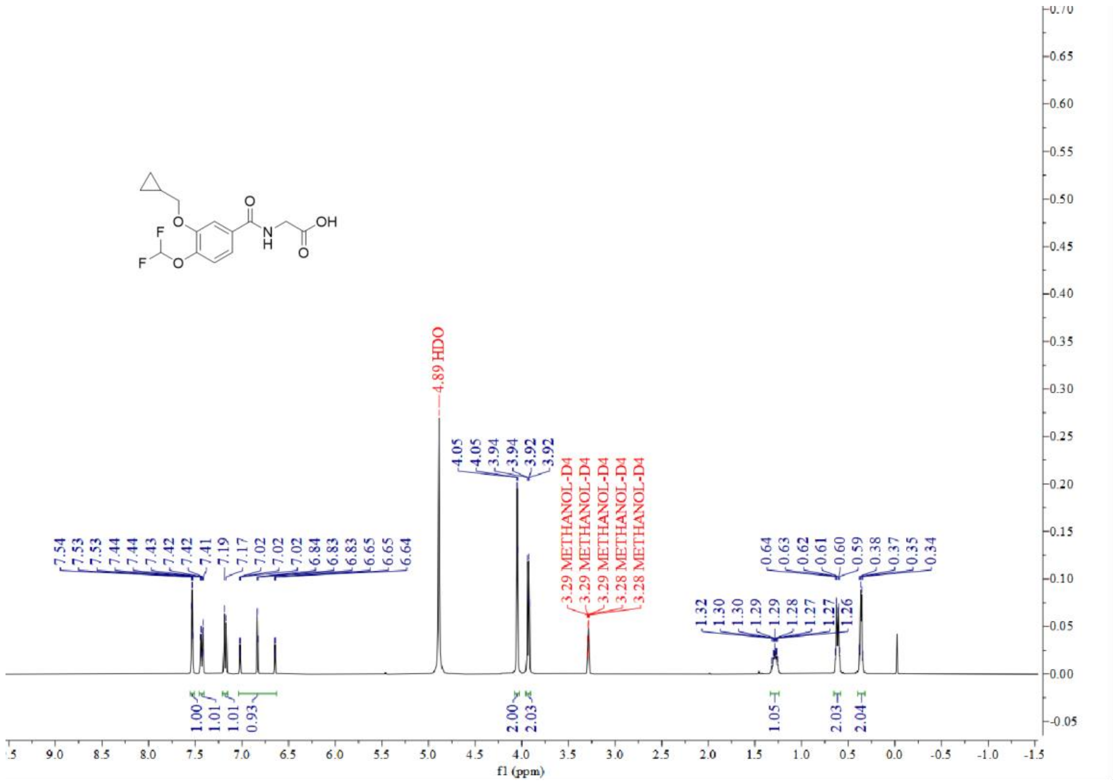

^{1}H NMR of compound D6(400MHz CD_3OD)

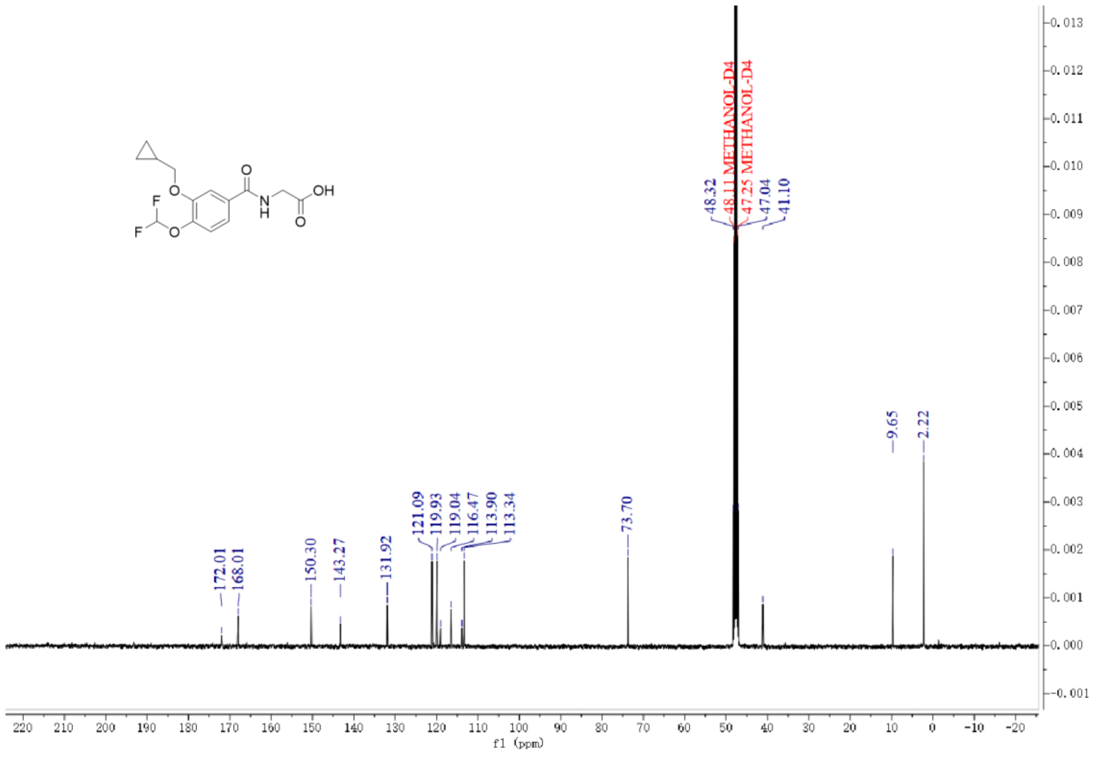

^{13}C NMR of compound D6(400MHz CD_3OD)

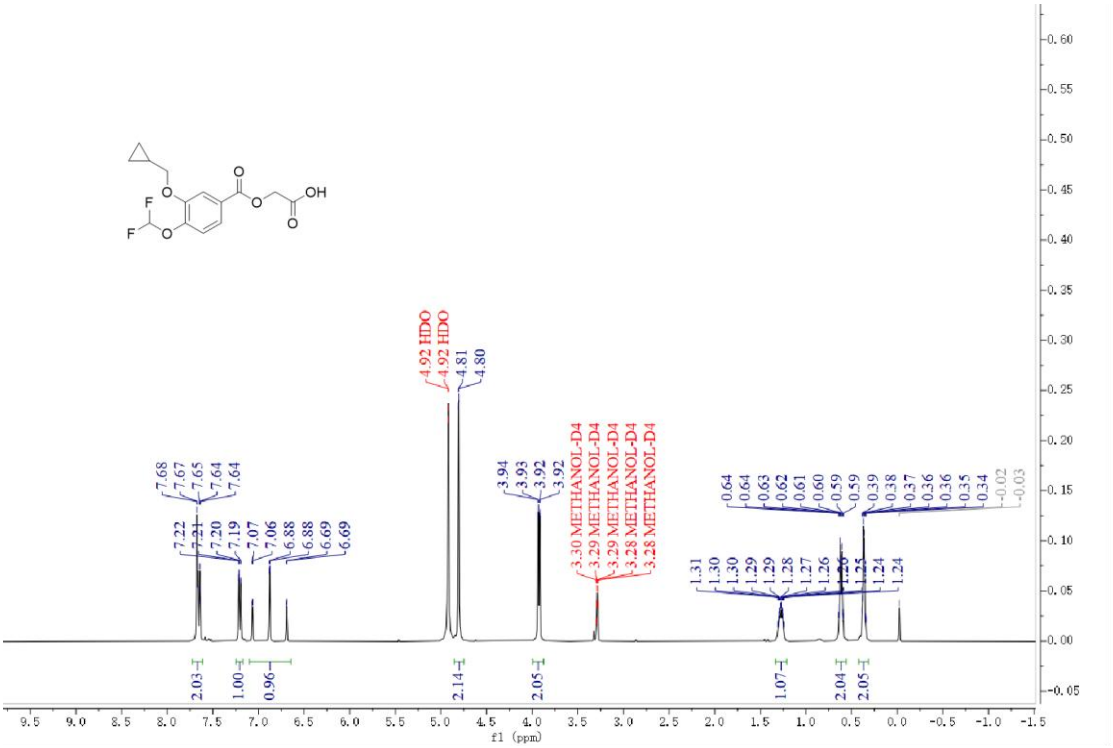

^{1}H NMR of compound D7(400MHz CD_3OD)

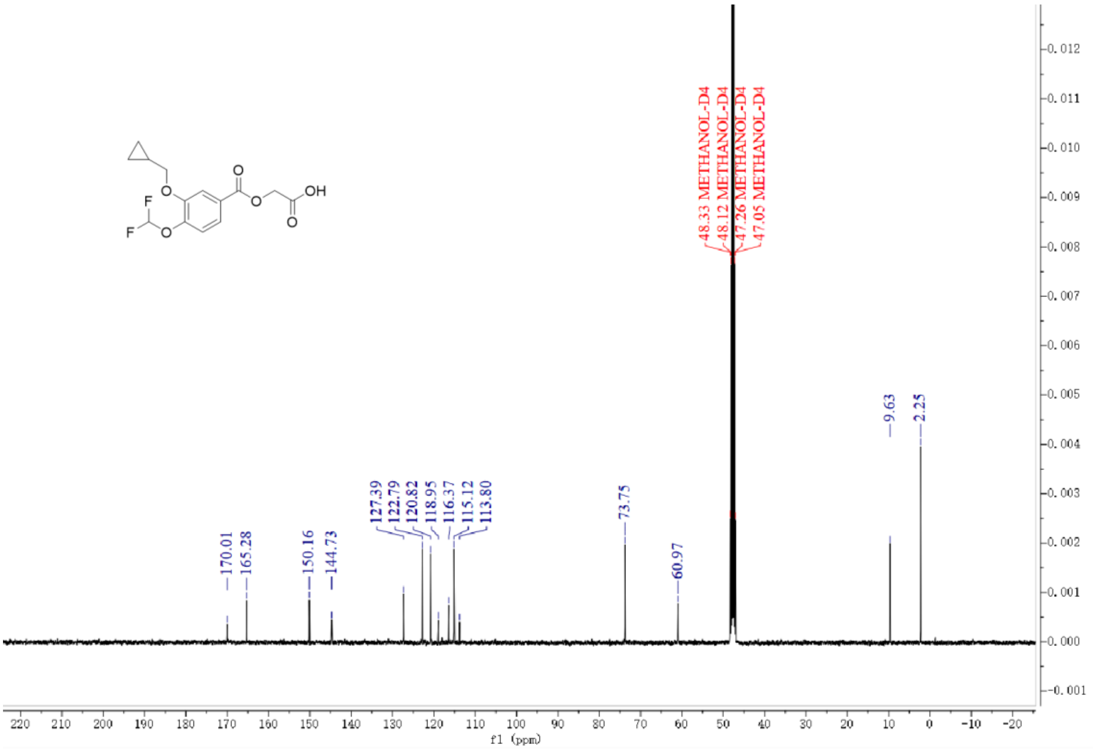

^{13}C NMR of compound D7(400MHz CD_3OD)

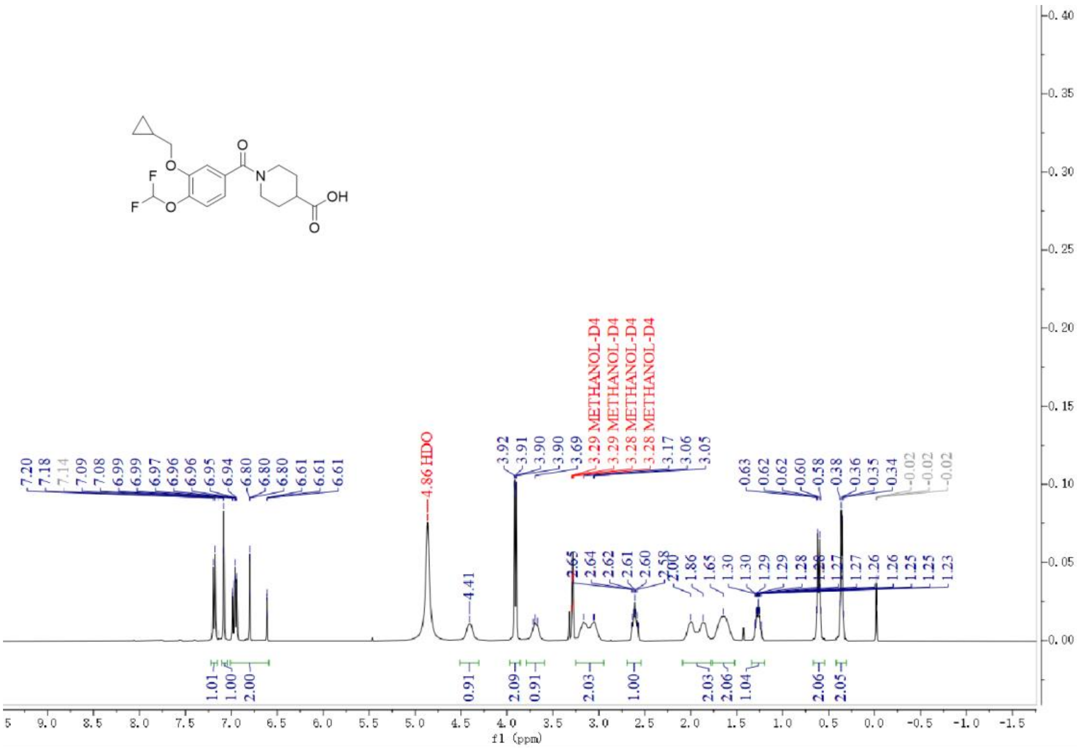

^{1}H NMR of compound D8(400MHz CD_3OD)

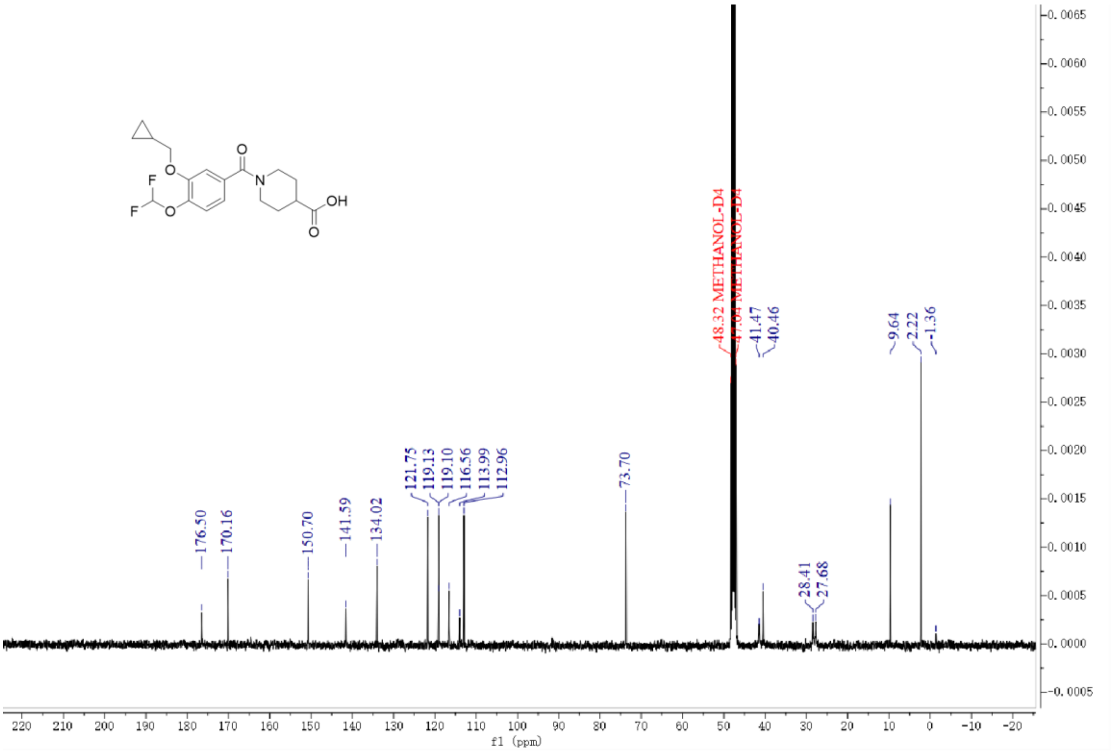

^{13}C NMR of compound D8(400MHz CD_3OD)

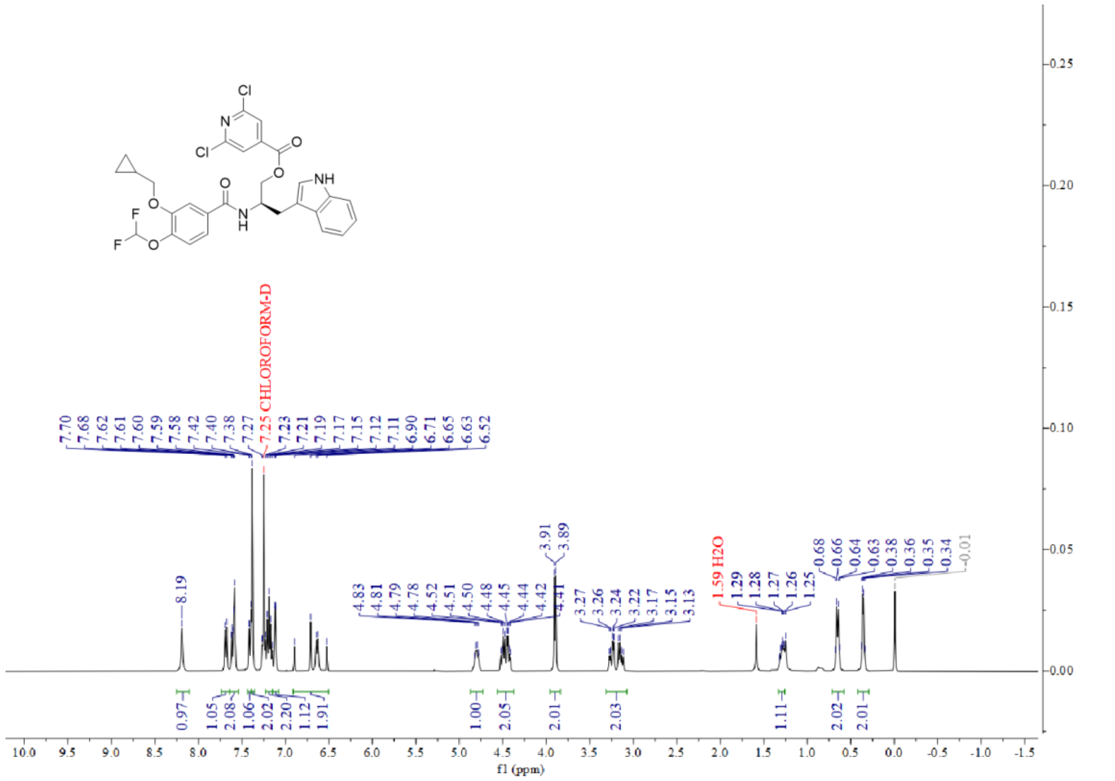

^{1}H NMR of compound E1(400MHz $CDCl_3$)

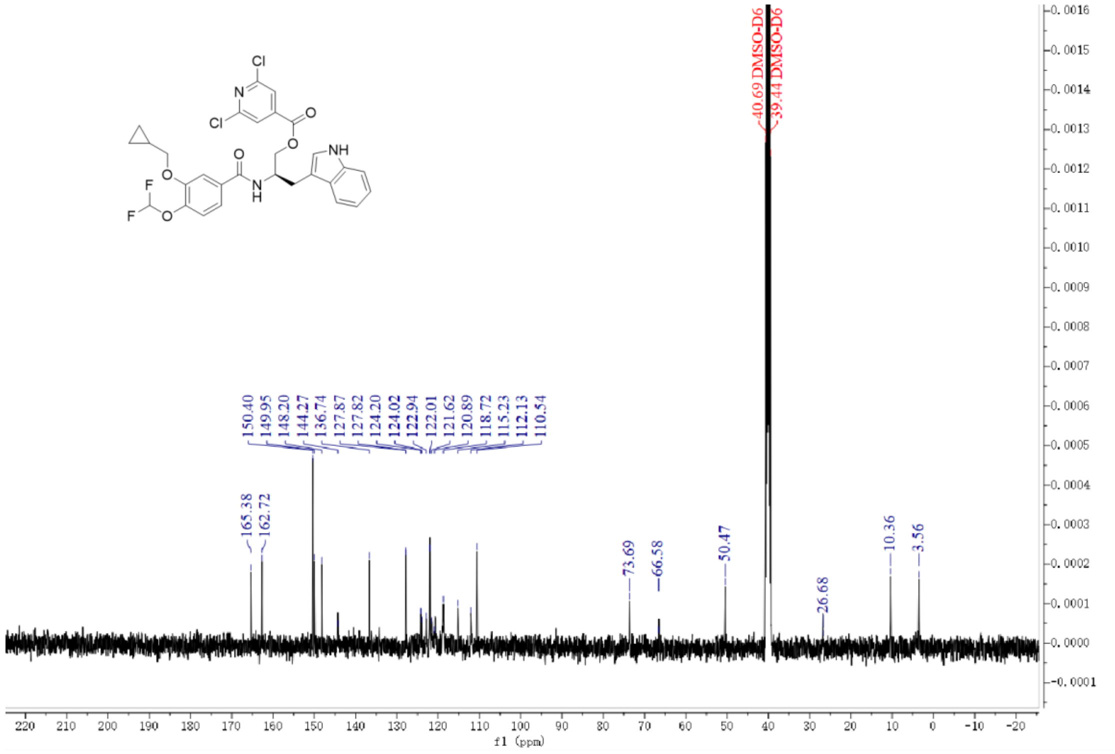

^{13}C NMR of compound E1(400MHz DMSO-*d*6)

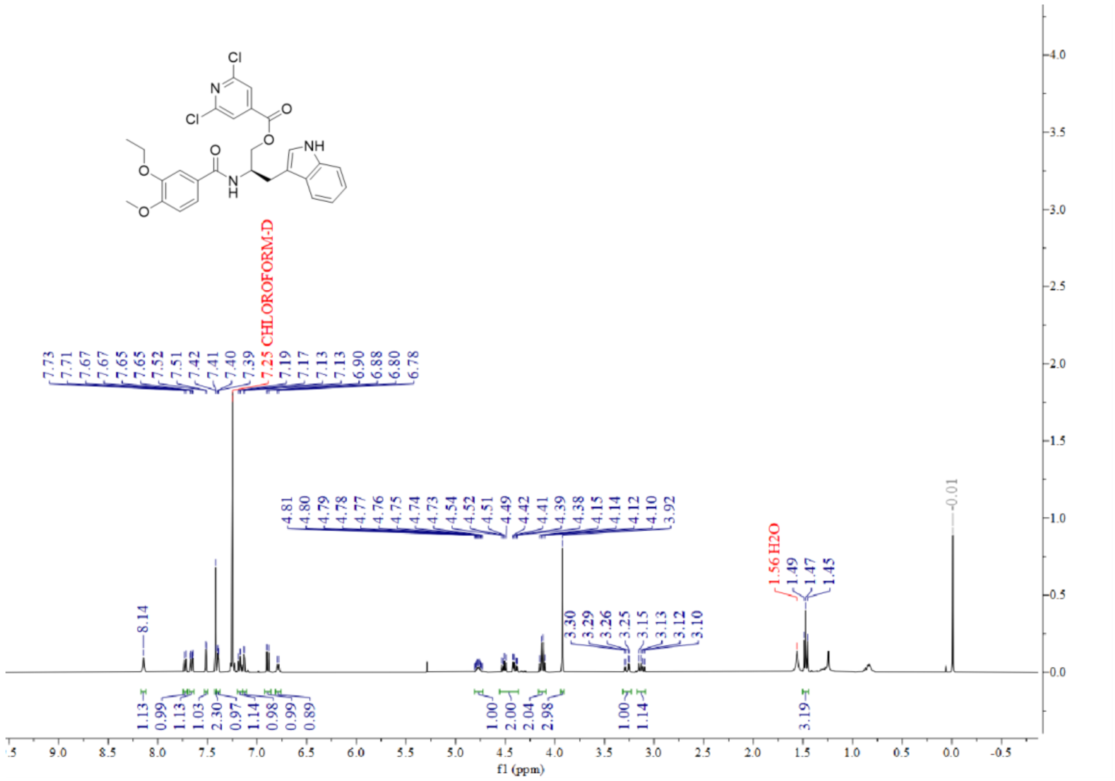

^{1}H NMR of compound E2(400MHz $CDCl_3$)

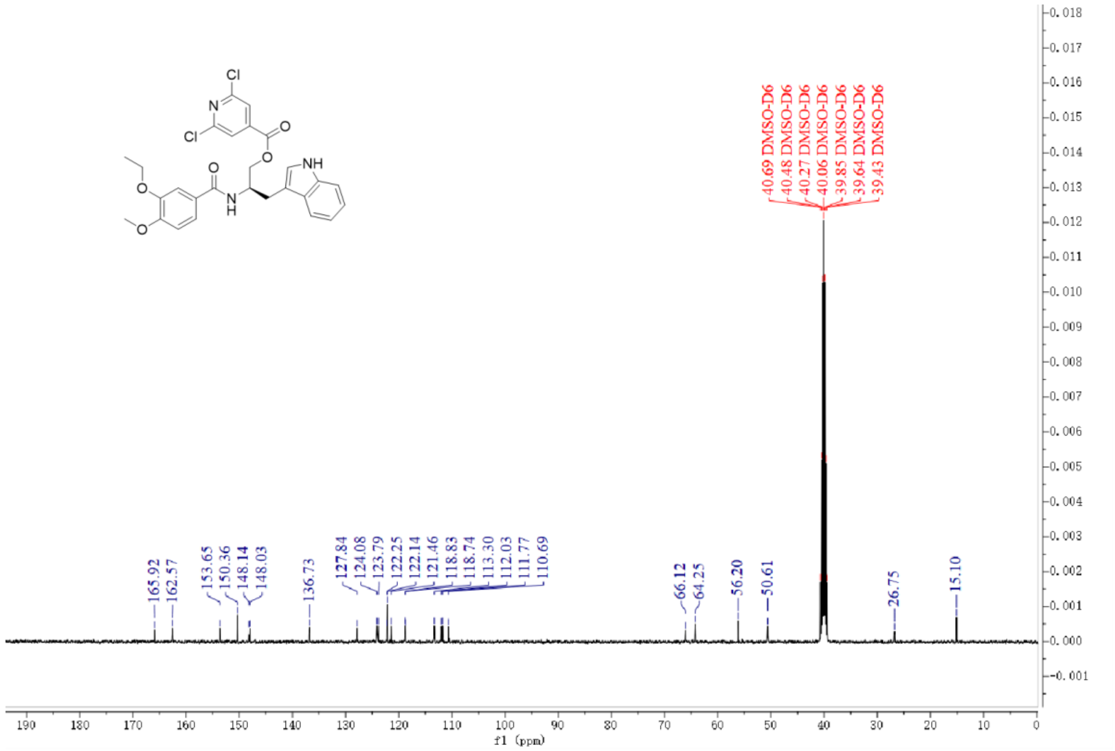

^{13}C NMR of compound E2(400MHz DMSO-*d*6)

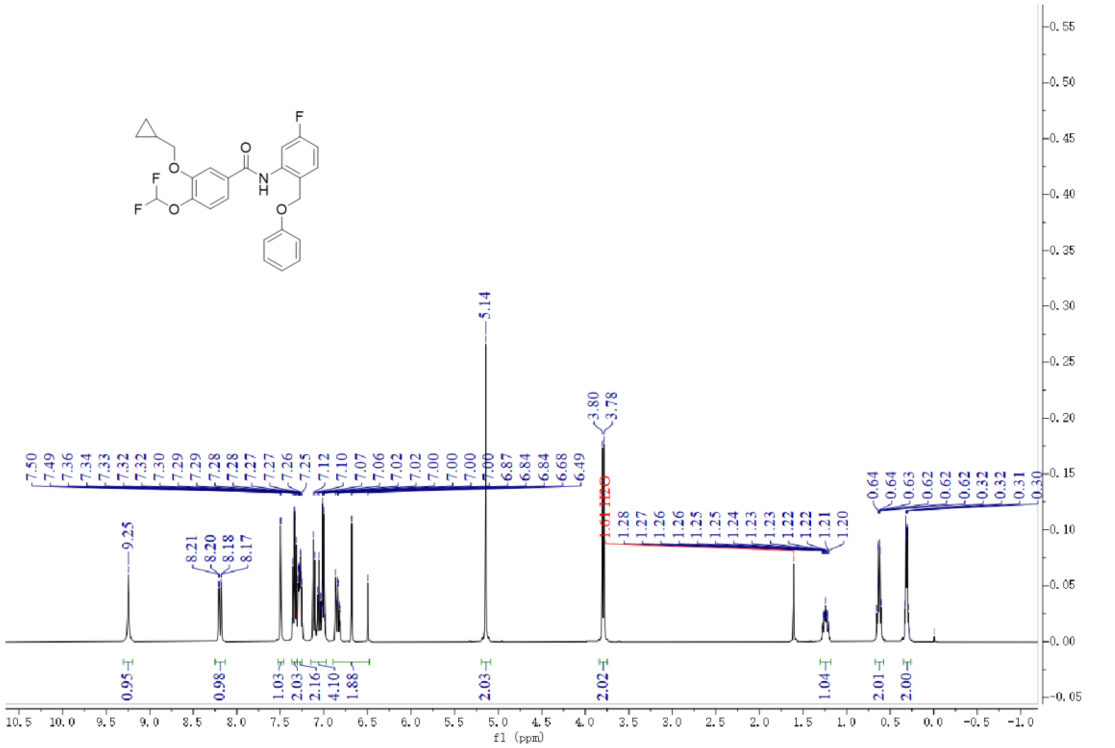

^{1}H NMR of compound E3(400MHz $CDCl_3$)

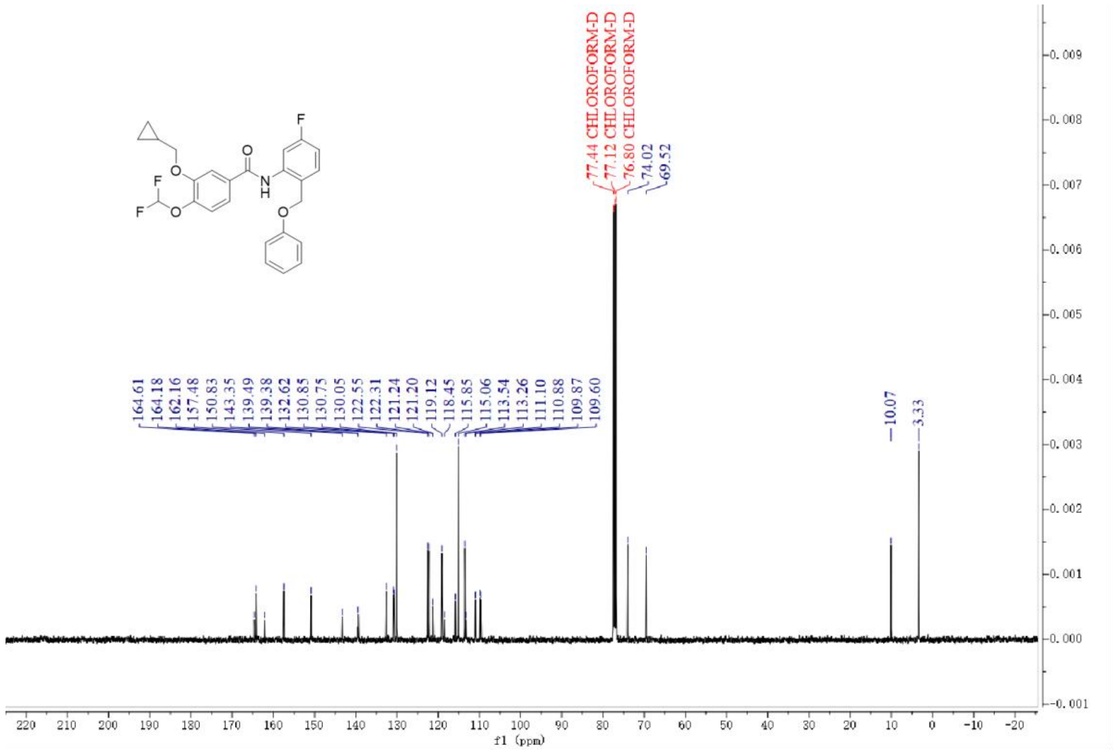

^{13}C NMR of compound E3(400MHz $CDCl_3$)

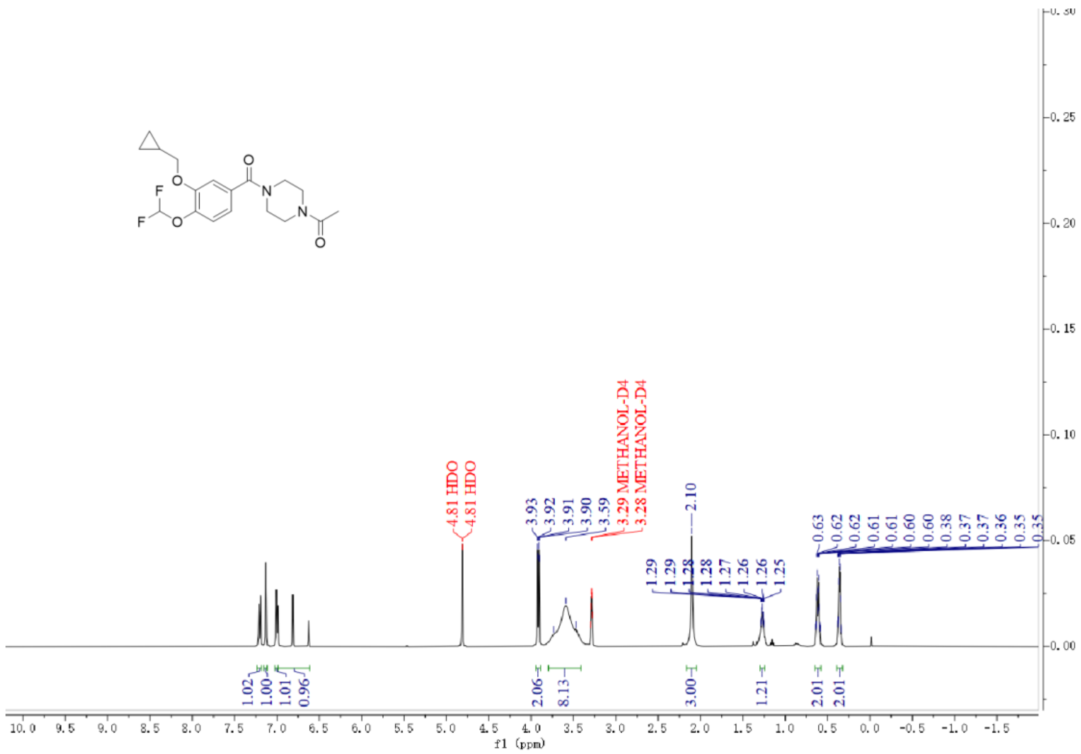

^{1}H NMR of compound E4(400MHz CD_3OD)

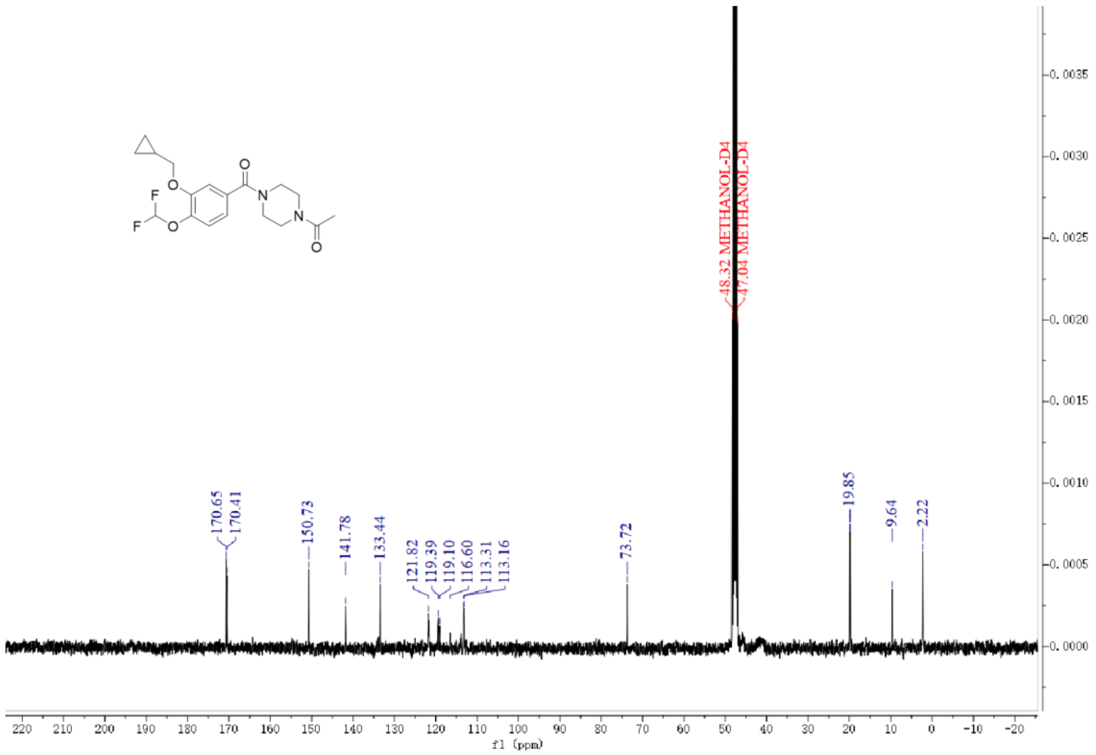

^{13}C NMR of compound E4(400MHz CD_3OD)
